# An mTOR Signaling Modulator Suppressed Heterotopic Ossification of Fibrodysplasia Ossificans Progressiva

**DOI:** 10.1016/j.stemcr.2018.10.007

**Published:** 2018-11-01

**Authors:** Kyosuke Hino, Chengzhu Zhao, Kazuhiko Horigome, Megumi Nishio, Yasue Okanishi, Sanae Nagata, Shingo Komura, Yasuhiro Yamada, Junya Toguchida, Akira Ohta, Makoto Ikeya

**Affiliations:** 1 iPS Cell-Based Drug Discovery, Sumitomo Dainippon Pharma Co., Ltd., Osaka 554-0022, Japan; 2 Department of Cell Growth and Differentiation, Center for iPS Cell Research and Application, Kyoto University, Kyoto 606-8507, Japan; 3 Department of Clinical Application, Center for iPS Cell Research and Application, Kyoto University, 53 Kawahara-cho, Shogoin, Sakyo-ku, Kyoto 606-8507, Japan; 4 Department of Regeneration Sciences and Engineering, Institute for Frontier Life and Medical Sciences, Kyoto University, Kyoto 606-8507, Japan; 5 Department of Fundamental Cell Technology, Center for iPS Cell Research and Application, Kyoto University, Kyoto 606-8507, Japan; 6 Department of Orthopaedic Surgery, Gifu University Graduate School of Medicine, Gifu 501-1194, Japan; 7 Department of Life Science Frontiers, Center for iPS Cell Research and Application, Kyoto University, Kyoto 606-8507, Japan; 8 Institute for Advancement of Clinical and Translational Science (iACT), Kyoto University Hospital, Kyoto 606-8507, Japan; 9 Department of Orthopaedic Surgery, Graduate School of Medicine, Kyoto University, Kyoto 606-8507, Japan

**Keywords:** mammalian target of rapamycin (mTOR), induced pluripotent stem cell (iPSC), fibrodysplasia ossificans progressiva (FOP), endochondral ossification, heterotopic ossification, bone morphogenetic protein (BMP), transforming growth factor β (TGF-β), activin A, high-throughput screening (HTS), ACVR1

## Abstract

Fibrodysplasia ossificans progressiva (FOP) is a rare and intractable disorder characterized by extraskeletal bone formation through endochondral ossification. FOP patients harbor gain-of-function mutations in ACVR1 (FOP-ACVR1), a type I receptor for bone morphogenetic proteins. Despite numerous studies, no drugs have been approved for FOP. Here, we developed a high-throughput screening (HTS) system focused on the constitutive activation of FOP-ACVR1 by utilizing a chondrogenic ATDC5 cell line that stably expresses FOP-ACVR1. After HTS of 5,000 small-molecule compounds, we identified two hit compounds that are effective at suppressing the enhanced chondrogenesis of FOP patient-derived induced pluripotent stem cells (FOP-iPSCs) and suppressed the heterotopic ossification (HO) of multiple model mice, including FOP-ACVR1 transgenic mice and HO model mice utilizing FOP-iPSCs. Furthermore, we revealed that one of the hit compounds is an mTOR signaling modulator that indirectly inhibits mTOR signaling. Our results demonstrate that these hit compounds could contribute to future drug repositioning and the mechanistic analysis of mTOR signaling.

## Highlights

• Established a screening system for fibrodysplasia ossificans progressiva (FOP)
• Identified two hit compounds that are effective in multiple FOP model mice
• An mTOR signaling modulator opens the door to a therapeutic strategy

## Graphical Abstract

**Figure:**
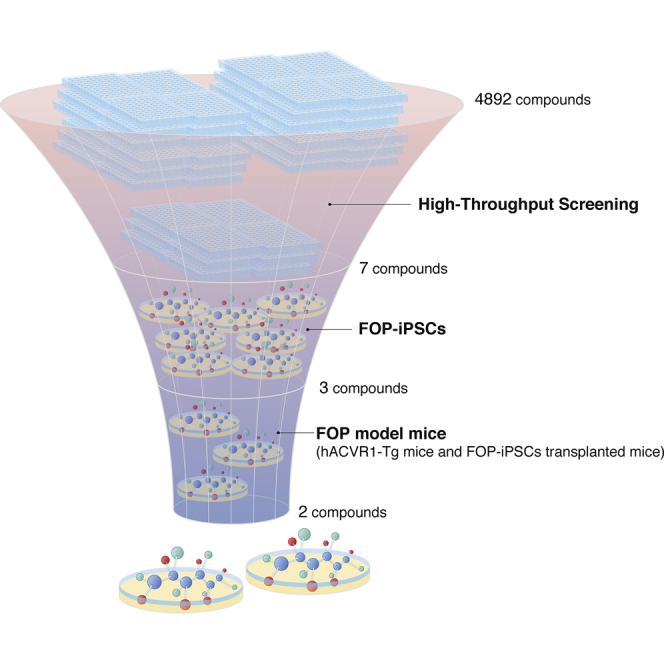

## Teaser

Focusing on the ligand-independent constitutive activation of mutated ACVR1 in fibrodysplasia ossificans progressiva (FOP) patients, Ikeya and colleagues have identified two hit compounds that were effective in multiple FOP model mice. One of the hit compounds, TAK 165, was an mTOR signaling modulator that indirectly regulated enhanced mTOR signaling. These findings shed light on a therapeutic strategy for FOP.

## Introduction

Fibrodysplasia ossificans progressiva (FOP) is a rare genetic disease characterized by extraskeletal bone formation in soft tissue, including skeletal muscle, ligament, and tendon, where bone is not normally observed. Such ectopic bones are formed through endochondral ossification, a process whereby bone tissue replaces mature cartilage (Kaplan et al., 2005, Kaplan et al., 2007, Kaplan et al., 2008, Kaplan et al., 2012a, Shore et al., 2005, Shore and Kaplan, 2010, Zuscik et al., 2008). Approximately 90% of FOP patients share an R206H (617G>A) point mutation in the intracellular glycine- and serine-rich domain of ACVR1 (Shore et al., 2006), a type I receptor for bone morphogenetic proteins (BMPs) (Canalis et al., 2003, Gu et al., 1999, Hogan, 1996, Massague et al., 2000, Mishina et al., 1999, Miyazono et al., 2010, Mueller and Nickel, 2012, Piek et al., 1999, Urist, 1965, Wozney et al., 1988). This mutated ACVR1 (FOP-ACVR1) has been shown to confer ligand-independent constitutive activity and ligand-dependent hyperactivity in BMP signaling (Billings et al., 2008, Chaikuad et al., 2012, Fukuda et al., 2008). Moreover, by utilizing FOP patient-derived induced pluripotent stem cells (FOP-iPSCs) and FOP-ACVR1 conditional-on knockin mice, it has been shown that as its neofunction FOP-ACVR1 abnormally transduces BMP signaling in response to activin A, a molecule that normally transduces transforming growth factor β (TGF-β) signaling but not BMP signaling (Hatsell et al., 2015, Hino et al., 2015).

A number of studies have revealed drug candidates for FOP, including direct kinase inhibitors of the catalytic domain of BMP type I receptors, which consequently suppress the phosphorylation of the downstream effectors SMAD1/5/8 (Engers et al., 2013, Hamasaki et al., 2012, Hao et al., 2010, Mohedas et al., 2013, Sanvitale et al., 2013, Yu et al., 2008); RARγ agonists, which reduce the expression of SMAD1/5/8 by protein degradation (Chakkalakal et al., 2016, Pavey et al., 2016, Shimono et al., 2011, Sinha et al., 2016); an inhibitor of activin A signaling by an activin A-specific neutralizing antibody (Hatsell et al., 2015, Hino et al., 2015); mechanistic target of rapamycin (mTOR) inhibitors, which target enhanced chondrogenesis, hypoxic signaling, and inflammatory signaling (Agarwal et al., 2016, Hino et al., 2017); and others (Brennan et al., 2017, Cappato et al., 2016, Convente et al., 2017, Kaplan et al., 2012b, Kitoh et al., 2013, Takahashi et al., 2012, Wang et al., 2016). Among these drug candidates, the RARγ agonist palovarotene, the anti-activin A antibody, and the mTOR inhibitor rapamycin are now under clinical trial. Although many attempts are ongoing, no drug is available for FOP, and a limited number of target molecules is reported.

For the identification of potential drug target molecules or pathways, phenotypic screenings that focus on the FOP pathology are an attractive approach but generally highly challenging to develop (Moffat et al., 2017). We previously reported phenotypic screening to modulate the enhanced chondrogenesis of FOP-iPSC-derived induced mesenchymal stromal cells (FOP-iMSCs) triggered by activin A (Hino et al., 2017). In that strategy, our concept was mainly based on the knowledge that trauma, surgery, inflammation, or viral infection often evoke episodic flare-ups that precede heterotopic ossification (HO) in FOP (Kaplan et al., 2005) and that one of the crucial initiators of HO is activin A activation (Hatsell et al., 2015, Hino et al., 2015). In contrast, another study reported a distinct feature of FOP pathology in that about half of FOP patients experienced the progression of HO without apparent flares, injury, or related events (Pignolo et al., 2016). Accordingly, we assumed that this pathology might be caused by ligand-independent constitutive activity such that FOP-ACVR1 transduces BMP signaling without ligand binding.

Featuring ligand-independent constitutive activity, here we established a phenotypic assay-based high-throughput screening (HTS) system focused on alkaline phosphatase (ALP), a well-established prehypertrophic chondrogenic marker (Zuscik et al., 2008), utilizing a chondrogenic ATDC5 cell line (Akiyama et al., 2000, Shukunami et al., 1997) that stably expresses FOP-ACVR1 (ATDC5/FOP-ACVR1). After HTS of approximately 5,000 small-molecule compounds, we identified three hit compounds: AZD0530 (also known as saracatinib), PD 161570, and TAK 165 (also known as mubritinib). These compounds suppressed the enhanced chondrogenesis in FOP-iMSCs, a critical step of HO in the FOP pathology. We subsequently showed their therapeutic effects on HO in three different *in vivo* models: a BMP-7-induced HO model, FOP model mice expressing FOP-ACVR1, and a FOP-iPSC-based HO model in which ectopic bones derived from FOP patient-derived cells are formed in mice. Mechanism-of-action studies indicated that AZD0530 and PD 161570 were inhibitors of both BMP and TGF-β signaling. On the other hand, TAK 165 was an mTOR signaling modulator that indirectly controlled mTOR signaling. These data extend the molecular basis of the HO induced in FOP patients.

## Results

### Development of an HTS System Focused on Constitutive Activity of FOP-ACVR1

**Figure 1:**
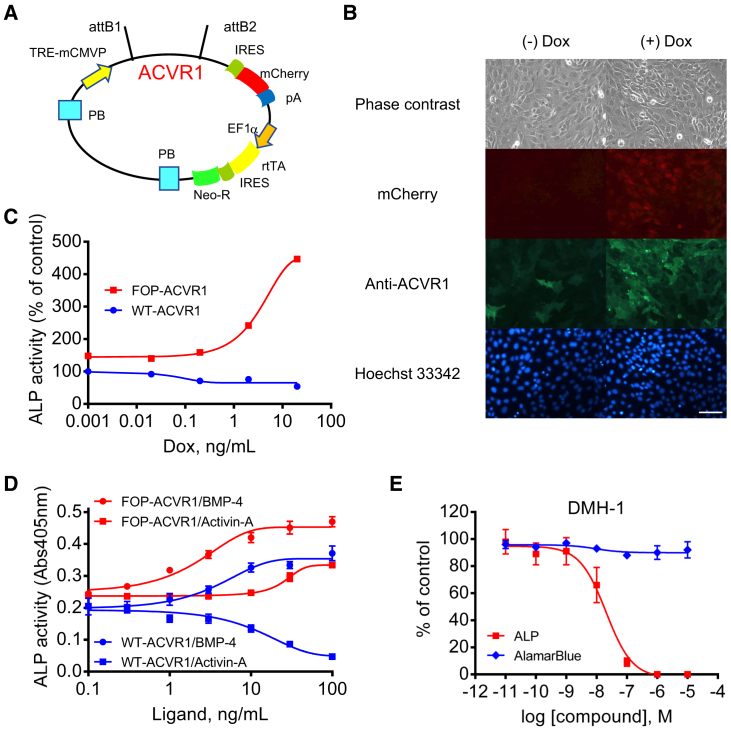
Construction and Validation of the Compound Screening System (A) Vector map of the Dox-inducible ACVR1 expression vector. (B) The expression of ACVR1 and mCherry in ATDC5/FOP-ACVR1 24 hr after 2 ng/mL Dox treatment. Scale bar, 100 μm. (C) ALP activity of ATDC5/WT-ACVR1 or FOP-ACVR1 72 hr after Dox treatment. (D) Concentration response curves of BMP-4 and activin A in ATDC5/WT-ACVR1 or FOP-ACVR1 72 hr after 3 ng/mL Dox and ligand treatment. (E) DMH-1 (ACVR1 kinase inhibitor) inhibited the ALP activity but not the viability (AlamarBlue) of ATDC5/FOP-ACVR1. ALP and AlamarBlue assays were performed 72 hr after Dox and DMH-1 treatment. Results are the mean ± SE, n = 1 (C) or biological triplicate in three independent experiments (D and E).

FOP-ACVR1 has been shown to render ligand-independent constitutive activity and ligand-dependent hyperactivity in BMP signaling (Billings et al., 2008, Chaikuad et al., 2012, Fukuda et al., 2008), and direct ACVR1 kinase inhibitors of the catalytic domain of BMP type I receptors are reported (Engers et al., 2013, Hamasaki et al., 2012, Hao et al., 2010, Mohedas et al., 2013, Sanvitale et al., 2013, Yu et al., 2008). Although these inhibitors are promising and effective on FOP model mice (Dey et al., 2016, Yu et al., 2008), new drug candidates that modulate FOP pathological conditions through undescribed mechanisms are also beneficial. Therefore, to screen direct BMP signaling inhibitors and FOP phenotype modulators at the same time, we focused on a chondrogenic cell line, ATDC5. ATDC5 cells are known to increase the expression of ALP by BMP stimulation in several days (Akiyama et al., 2000, Shukunami et al., 1997), and ALP activity can be detected by a chromogenic phosphatase substrate in an HTS format. Although ALP is also known to be a pluripotent marker, it is upregulated during chondrogenic induction consistently with other chondrogenic markers in ATDC5 cells (Shukunami et al., 1997), indicating that ALP is a chondrogenic marker at least in ATDC5 cells. We designed an ACVR1 expression vector utilizing the doxycycline (Dox)-inducible vector KW111 (Hayakawa et al., 2013, Woltjen et al., 2009) and generated ATDC5 cells stably expressing FOP-ACVR1 (R206H) or wild-type (WT)-ACVR1 (Figure 1A). After Dox treatment, ACVR1 expression was increased in a concentration-dependent manner (Figures 1B and S1). Expectedly, without BMP stimulation, ALP activity was increased in ATDC5 cells expressing FOP-ACVR1, but not in WT-ACVR1 (Figure 1C). This result indicates the constitutive activity of BMP signaling was triggered by FOP-ACVR1 expression. In addition to this constitutive activity, hyperactivity against BMP-4 and acquired responsiveness to activin A were observed in ATDC5-expressing FOP-ACVR1 (Figure 1D). These results indicated the validity of our assay system. DMH-1, a direct ACVR1 kinase inhibitor, suppressed the ALP activity of ATDC5 cells expressing FOP-ACVR1 without BMP stimulation in a concentration-dependent manner, also demonstrating that the constitutive activity of BMP signaling can be measured by ALP activity (Figure 1E). These results indicate that Dox-inducible ATDC5 cells enable us to screen inhibitors against the constitutive activity of FOP-ACVR1.

### HTS and Follow-Up Screens Identified Seven Hit Compounds

**Figure 2:**
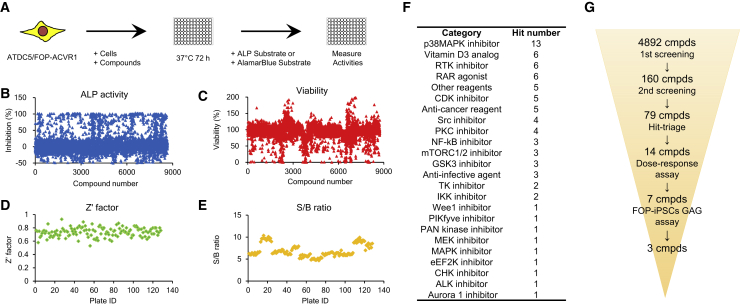
Schematic and Detailed Results of High-Throughput Screening (A) Schematic of the first screening. (B–E) Scatterplot distribution of ALP activity (B), viability (C), Z′ factor (D), and S/B ratio (E) from the first screening against 4,892 compounds. (F) Classification of 79 hit compounds through the second screening. (G) Results of the HTS campaign and follow-up screens. Biological duplicates (B–F).

**Figure 3:**
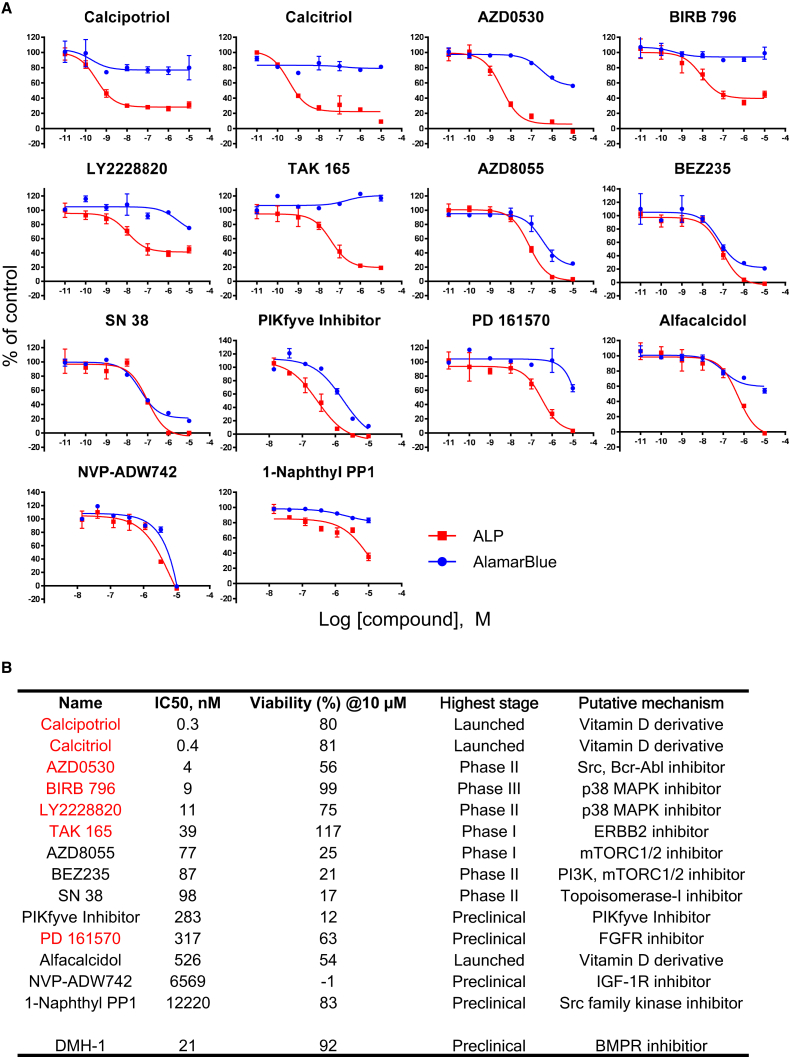
Detailed Dose-Response Assay Results of 14 Hit Compounds (A) Dose-response curves of 14 hit compounds. ALP assay and AlamarBlue assay were performed using the same protocol as the HTS. (B) IC_50_ values and viability (%) at 10 μM in the dose-response assay, highest stage, and putative mechanism of 14 hit compounds are shown. Seven compounds (red) satisfied the criteria (IC_50_ of ALP assay <500 nM and viability at 10 μM >50%). Results are the mean ± SE, biological triplicates.

Utilizing this HTS system, we performed a first screening (n = 2; test compounds = 1 μM, Figure 2A) against our HTS library, which contains approximately 5,000 small-molecule compounds, most of which are marketed or bioactive (see also Supplemental Experimental Procedures). The scatterplot distribution of ALP activity and cell viability (Figures 2B and 2C), and Z′ factor and S/B ratio (Figures 2D and 2E) confirmed the validity of the HTS campaign. From the first screening, we obtained 160 hit compounds that fulfilled the criteria that more than 40% inhibition of ALP activity against DMSO control cells, less than 40% inhibition of viability and more than 20% of margin (inhibition of ALP activity [%] minus inhibition of viability [%]). A second screening was performed against the above 160 compounds (n = 2; test compounds = 0.1, 0.3, 1, 3 μM), and we identified 79 hit compounds that showed 40% inhibition of ALP activity against DMSO control cells and more than 50% of margin at any dose (Figures 2F and S2). A summary of HTS is shown in Figure 2G. Among them, RARγ agonists suppressed ALP activity, indicating the accuracy of our HTS system. To explore compounds that have potential to identify new mechanisms or contribute to future drug repositioning, we selected 14 compounds and performed a detailed concentration-dependent assay (Figure 3A). As a result, we identified seven compounds that showed stronger IC_50_ (<500 nM) and less toxicity (viability at 10 μM >50%) through our HTS campaign focused on the constitutive activity of FOP-ACVR1 (Figure 3B, red).

### Further Validation of Seven Hit Compounds in FOP Patient-Derived iPSCs

**Figure 4:**
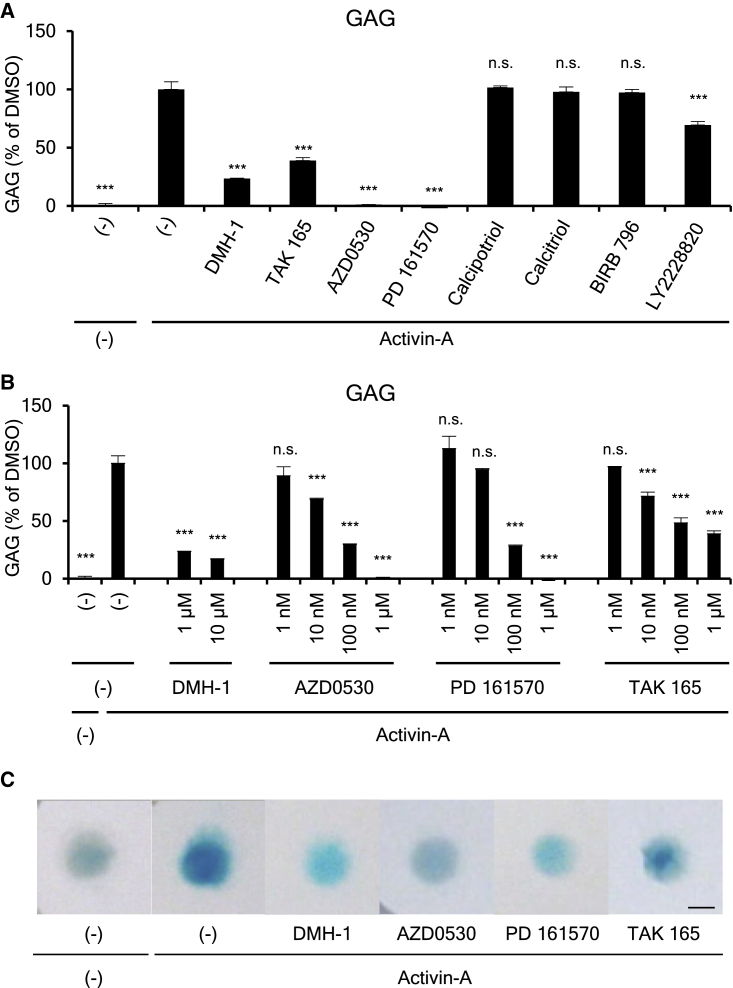
AZD0530, PD 161570, and TAK 165 Suppressed the Chondrogenic Induction of FOP-iMSCs (A) The inhibitory effect of seven hit compounds on the chondrogenic induction of FOP-iMSCs. The cells were harvested 7 days after chondrogenic induction, which was performed with or without activin A and inhibitors (1 μM). (B) AZD0530, PD 161570, and TAK 165 suppressed the chondrogenic induction of FOP-iMSCs in a dose-dependent manner. (C) Alcian blue staining of DMH-1, AZD0530, PD 161570, and TAK 165. Results are the mean ± SE, biological triplicates used FOP-iPSCs (vFOP4-1) (A and B). n.s., no significant difference; ^∗∗∗^p < 0.001 by Dunnett's multiple comparisons t test compared with the DMSO treatment control with activin A. Scale bar, 200 μm.

To predict these seven compounds' therapeutic effects on FOP patients, we performed a FOP-iPSC-based chondrogenic assay. In this assay system, FOP-iMSCs (Fukuta et al., 2014, Hino et al., 2015, Hino et al., 2017, Matsumoto et al., 2015), a putative cell of origin of ectopic chondrogenesis, were treated with activin A, and the inhibitory effect of seven hit compounds was assessed at 1 μM (Figure 4A). Among them, AZD0530, PD 161570, and TAK 165 showed potent inhibition on glycosaminoglycan (GAG) production, which represents the amount of extracellular matrix secreted by chondrocytes. A detailed analysis against these three compounds revealed a concentration-dependent inhibitory effect on GAG in the chondrogenic assay of FOP-iMSCs (Figure 4B). Alcian blue staining, which stains acidic polysaccharides such as GAG in chondrocytes, also confirmed drug activity (Figure 4C). These results indicate that AZD0530, PD 161570, and TAK 165 have the potential to suppress the ectopic chondrogenesis of FOP patients.

### *In Vivo* Therapeutic Effects of AZD0530 and TAK 165

**Figure 5:**
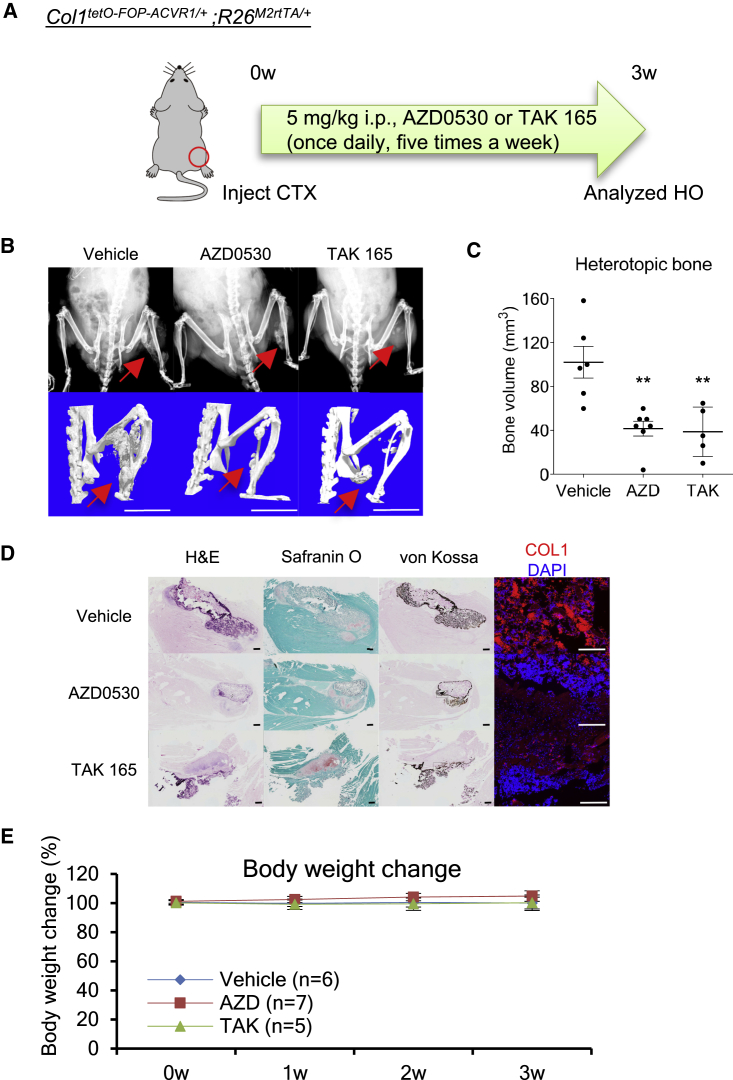
AZD0530 and TAK 165 Suppressed HO in FOP-ACVR1 Conditional Transgenic Mice (A) Schematic of the *in vivo* efficacy study utilized FOP-ACVR1 conditional transgenic mice. Intraperitoneal (i.p.) administration of 5 mg/kg AZD0530 and TAK 165 (once daily, five times a week) suppressed the HO in FOP-ACVR1 (R206H) conditional transgenic mice. HO was induced by muscle injury triggered by cardiotoxin (CTX) injection and oral administration of Dox. Three weeks after CTX injection and drug administration, mice were analyzed. (B) X-rays (upper panels) and μCT (lower panels) observations. Scale bars, 10 mm. (C) Average heterotopic bone volume. (D) Histological analysis of the CTX-injected region. H&E staining, safranin O staining (acidic proteoglycan), von Kossa staining (calcium), and anti-COL1 (bone) staining are shown. Scale bars, 100 μm (H&E, safranin O, and von Kossa) and 500 μm (COL1 and hVimentin). (E) Body weight change (%) of mice administered compounds. Results are the mean ± standard error (SE), n = 6 (vehicle), n = 7 (AZD0530), or n = 5 (TAK 165). ^∗∗^p < 0.01 by Dunnett's multiple comparisons t test compared with vehicle treatment group (C). No significant differences between the AZD- or TAK-administered group compared with the vehicle group in two-way repeated-measures ANOVA followed by Dunnett's multiple comparisons t test (E).

**Figure 6:**
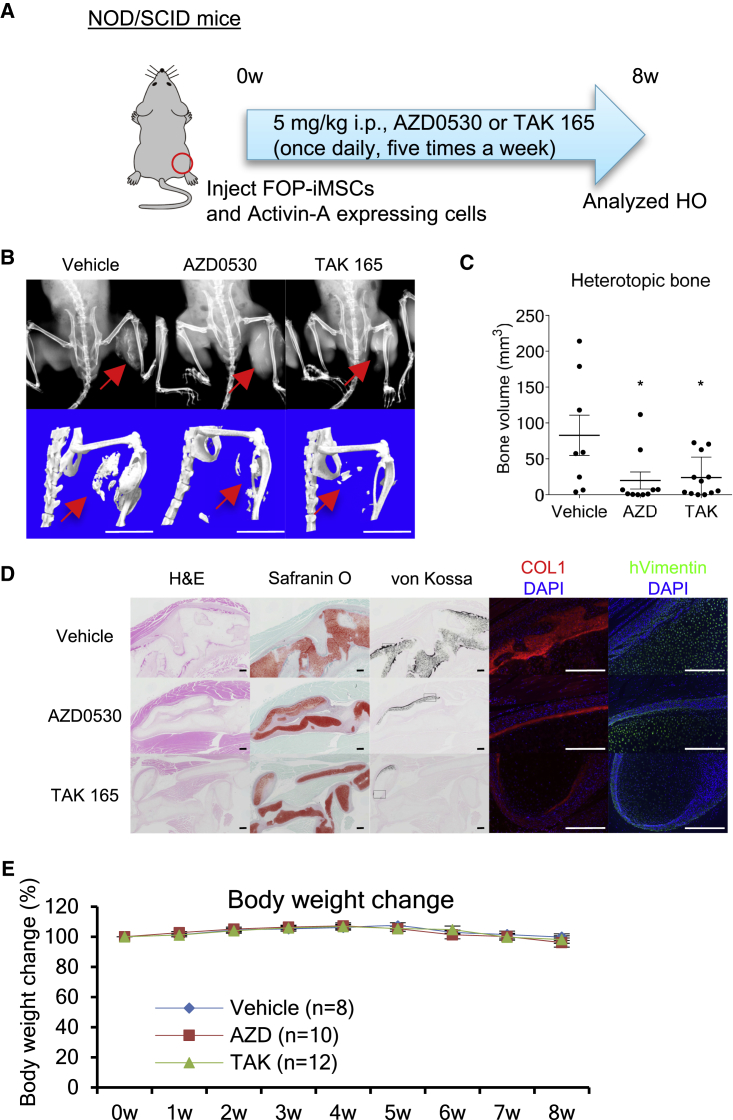
AZD0530 and TAK 165 Suppressed HO Derived from FOP-iMSCs *In Vivo* (A) Schematic of the *in vivo* efficacy study utilized a human FOP-iPSC-based *in vivo* model. Intraperitoneal (i.p.) administration of 5 mg/kg AZD0530 or TAK 165 (once daily, five times a week) suppressed the HO derived from FOP-iMSCs triggered by activin A. Eight weeks after transplantation and drug administration, mice were analyzed. (B) X-ray (upper panels) and μCT (lower panels) observations. Scale bars, 10 mm. (C) Average heterotopic bone volume. (D) Histological analysis of the cell-transplanted region. H&E, safranin O, von Kossa, anti-COL1, and anti-human vimentin staining are shown. Scale bars, 100 μm (H&E, safranin O, and von Kossa) and 500 μm (COL1 and hVimentin). (E) Body weight change (%) of mice administered compounds. Results are the mean ± SE, n = 8 (vehicle), n = 10 (AZD0530), or n = 12 (TAK165). ^∗^p < 0.05 by Dunnett's multiple comparisons t test compared with vehicle treatment group (C). No significant differences between the AZD- or TAK-administered group compared with the vehicle group in two-way repeated-measures ANOVA followed by Dunnett's multiple comparisons t test (E).

Next, the therapeutic effects of these drug candidates on FOP model mice were evaluated. We focused on AZD0530 and TAK 165 because they are applicable to *in vivo* experiments (Hennequin et al., 2006, Nagasawa et al., 2006). Previously, we generated FOP model mice that conditionally express hFOP-ACVR1 (R206H) by Dox administration and develop HO by muscle injury using cardiotoxin (CTX) (Hino et al., 2017). The intraperitoneal administration of AZD0530 or TAK 165 significantly suppressed the HO in these mice (Figures 5A–5C). In the CTX-injected site, we observed positive staining for safranin O (acidic proteoglycan, an extracellular matrix protein of chondrocytes), von Kossa (calcium deposition), and COL1 (bone marker) (Figure 5D, vehicle). On the other hand, mice administered AZD0530 or TAK 165 seemed to show less positive staining for von Kossa or COL1 (Figure 5D, AZD0530 and TAK 165). No apparent differences in body weight change was observed in mice administered AZD0530 or TAK 165 compared with vehicle (Figure 5E). These observations demonstrated that AZD0530 and TAK 165 are effective at suppressing HO in FOP model mice. These compounds' therapeutic effects were also confirmed in a BMP-7-induced HO model using WT mice (Figure S3). Furthermore, we validated whether AZD0530 and TAK 165 have the potential to suppress the HO of FOP patient-derived cells *in vivo*. We previously reported a human FOP-iPSC-based *in vivo* model (Hino et al., 2015, Hino et al., 2017). In this humanized FOP model, the transplantation of FOP-iMSCs and activin A-expressing cells into mice induces FOP patient-derived heterotopic bone *in vivo*. Notably, the administration of AZD0530 or TAK 165 significantly suppressed HO in these mice (Figures 6A–6C). Hypertrophic chondrocytes (based on safranin O and von Kossa staining) and von Kossa- and COL1-positive bone regions seemed to be fewer in mice administered AZD0530 or TAK 165 (Figure 6D). Because a large number of anti-human-specific vimentin-positive cells were observed in the AZD0530 and TAK 165-treated groups (Figure 6D), we could conclude that the therapeutic effect of these compounds was not due to the death of the human transplanted cells but rather the suppression of HO. In these experiments, neither AZD0530 nor TAK 165 administration decreased body weight (Figures 5E, 6E, and S3D), and the dosing used was comparable with that in previous studies (Hennequin et al., 2006, Nagasawa et al., 2006). TAK 165 in particular did not impair the chondrogenesis of normal chondrocytes (Figures S4A–S4C), normal skeletal development *in vivo* (Figures S4D and S4E), or wound healing *in vitro* (Figures S4F and S4G). Thus, we concluded the HO suppression was not primarily caused by toxicity, although further *in vivo* assessment might be preferable. Taken together, AZD0530 and TAK 165 are promising drug candidates since they suppressed the HO of FOP patient-derived cells *in vivo* in addition to the HO of FOP model mice.

### Mechanisms of Action of AZD0530, PD 161570, and TAK 165

**Figure 7:**
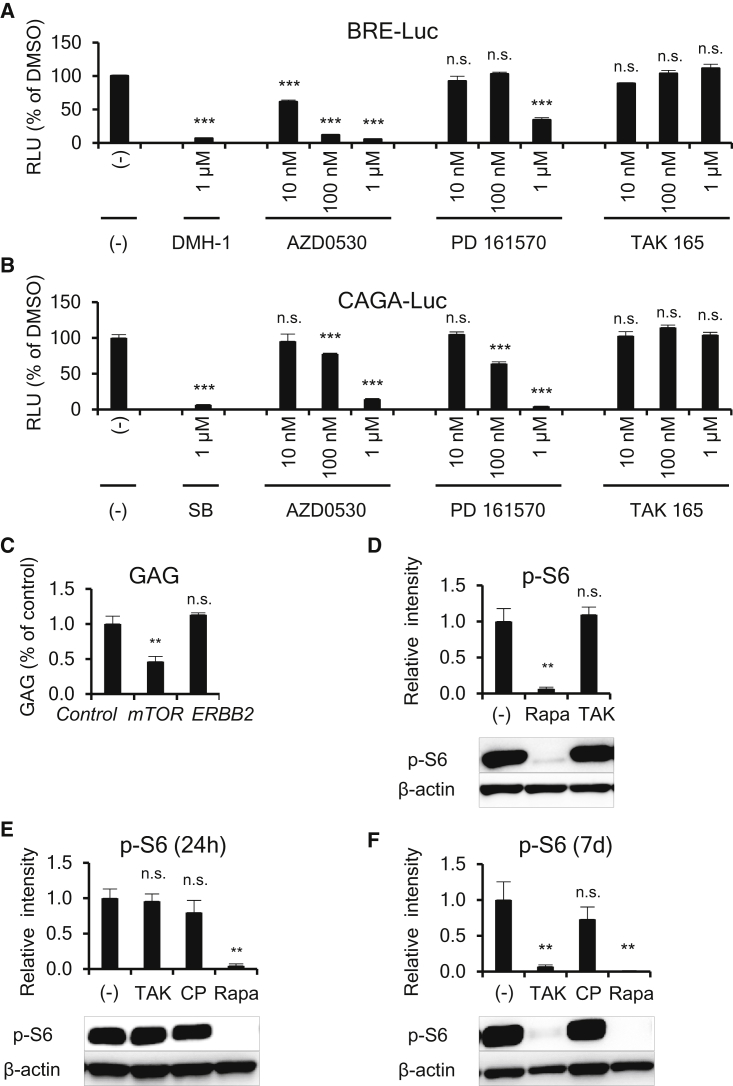
Mechanism of Action of AZD0530, PD 161570, and TAK 165 (A and B) AZD0530 and PD 161570, but not TAK 165, inhibited both BMP signaling (A) and TGF-β signaling (B). FOP-iMSCs transiently transfected with BRE-Luc (A) or CAGA-Luc (B) with CMV-*Renilla* were stimulated with activin A and compounds for 16 hr (A) or 3 hr (B). (C) *ERBB2* knockdown did not reduce GAG content in the chondrogenic assay of FOP-iMSCs. One day after siRNA transfection, chondrogenic induction with activin A was initiated, and after 7 days the cells were harvested. (D–F) TAK 165 indirectly inhibited mTOR signaling. (D) TAK 165 did not inhibit mTOR signaling directly, as assessed by western blotting of the phosphorylation of S6 (p-S6), a surrogate marker of mTORC1 activity. FOP-iMSCs cultured with 10% FBS were treated with 100 nM rapamycin (Rapa) or TAK 165 (TAK) for 2 hr, and the cells were harvested. (E and F) TAK 165 indirectly inhibited p-S6 during chondrogenic induction with activin A. After 24 hr or 7 days of chondrogenic induction of FOP-iMSCs with activin A and test compounds, the cells were harvested. 1 μM TAK, 1 μM CP (CP-724714, another selective ERBB2 inhibitor), or 10 nM Rapa were applied in the experiments. Results are the mean ± SE of biological quadruplicates (A and B) or triplicates (C–F) using FOP-iPSCs (vFOP4-1). n.s., no significant difference; ^∗∗^p < 0.01, ^∗∗∗^p < 0.001 by Dunnett's multiple comparisons t test compared with the siRNA-transfected negative control and activin A (C) or with the DMSO treatment control and activin A (A, B, D–F).

Finally, we analyzed the mechanisms of action of AZD0530, PD 161570, and TAK 165 on the chondrogenesis of FOP-iMSCs. Because it is known that BMP and TGF-β signaling are crucial in the chondrogenesis of FOP (Hino et al., 2015, Hino et al., 2017) and because our HTS system can detect BMP inhibitors, we assessed the direct effects of the three drugs on BMP and TGF-β signaling. AZD0530 and PD 161570 inhibited both BRE-Luc (BMP-specific luciferase reporter construct) and CAGA-Luc (TGF-β-responsive luciferase reporter construct) (Figures 7A and 7B). Therefore, we concluded AZD0530 and PD 161570 were BMP and TGF-β signaling dual inhibitors, and their mechanisms of action could contribute to the suppression of the chondrogenesis of FOP-iMSCs because they inhibited both pathways at similar drug concentration ranges during the chondrogenesis of FOP-iMSCs (Figure 4B). This result is in accordance with a previous study showing that AZD0530 inhibited BMP type I receptors (Lewis and Prywes, 2013). On the contrary, TAK 165 did not affect these signaling pathways. TAK 165 is an ERBB2 (also known as HER2)-selective kinase inhibitor (Anastassiadis et al., 2011, Nagasawa et al., 2006). To check the importance of ERBB2 inhibition in chondrogenesis, we performed a loss-of-function study using small interfering RNA (siRNA). Knockdown of *ERBB2* did not decrease GAG in the chondrogenesis of FOP-iMSCs (Figure 7C). Furthermore, another ERBB2-selective inhibitor (CP-724714), an ERBB1/2-selective inhibitor (lapatinib), or ERBB2-selective neutralizing antibodies (trastuzumab and pertuzumab) showed no effect on GAG in the chondrogenesis of FOP-iMSCs (Figures S5A and S5B). Given these results, the mechanism of action of TAK 165 was not through ERBB2 inhibition. We further investigated the effect of TAK 165 on TGF-β3-induced chondrogenesis in FOP-iMSCs and activin A-induced chondrogenesis in resFOP-iMSCs, in which the mutant ACVR1 was corrected to WT (Matsumoto et al., 2015) (Figures S5C and S5D). These results indicate that TAK 165 showed stronger effects on FOP cells than on normal cells. Recently, ourselves and Agarwal et al. have separately uncovered the impact of inhibiting mTOR signaling on the HO of FOP model mice and FOP-iMSCs (Agarwal et al., 2016, Hino et al., 2017). Therefore, we checked TAK 165's effect on mTOR signaling. First, to test whether TAK 165 is a direct inhibitor, we monitored the phosphorylation of S6 (p-S6), a well-known mTOR signaling surrogate marker, for 2 hr after treatment with TAK 165 in FOP-iMSCs cultured in 10% fetal bovine serum (FBS) (Figure 7D). In this condition, a strong p-S6 signal was detected. The mTOR inhibitor rapamycin decreased p-S6 levels, but TAK 165 did not. Next, we checked for indirect effects of TAK 165 on mTOR signaling in the chondrogenesis assay of FOP-iMSCs stimulated by activin A. After 24-hr stimulation with TAK 165 or CP-724714, no effects were observed on p-S6 (Figure 7E). Interestingly however, after 7 days of stimulation with TAK 165 but not CP-724714, p-S6 was dramatically decreased (Figure 7F). As expected, AZD0530 and PD 161570 significantly inhibited p-S6 levels from 2 hr after treatment (Figure S6), indicating that TAK 165 acts through a distinct mechanism. In addition, we performed an unbiased transcriptome analysis of FOP-iMSCs 7 days after inducing chondrogenesis by activin A (Figure S7). TAK 165, but not other ERBB2 inhibitors, affected genes that are involved in chondrogenesis or osteogenesis (“Role of Osteoblasts, Osteoclasts, and Chondrocytes in Rheumatoid Arthritis” in Figure S7B). These results indicate that TAK 165 indirectly modulated mTOR signaling and suppressed the chondrogenesis and HO of FOP.

## Discussion

In this report, we identified TAK 165 as a drug candidate for FOP. It is reported that TAK 165 is a selective inhibitor of ERBB2, a receptor tyrosine kinase often amplified or mutated in several cancers (Moasser, 2007). It is common that kinase inhibitors targeting catalytic domains show less selectivity, but interestingly TAK 165 is highly selective for ERBB2 against a panel of 300 recombinant protein kinases, presumably due to the fact that TAK 165 is an allosteric inhibitor of ERBB2 (Anastassiadis et al., 2011). Regardless of TAK 165's high selectivity to ERBB2, the suppression of chondrogenesis and HO by TAK 165 was not caused by ERBB2 inhibition but by indirect mTOR signaling inhibition (Figure 7). TAK 165 inhibited the ALP activity of constitutively activated FOP-ACVR1 in ATDC5 (Figure 3) and the enhanced chondrogenesis of FOP-iMSCs triggered by activin A (Figure 4). TAK 165 also modulated chondrogenesis-related pathways (Figure S7). These results suggested that TAK 165 affected chondrogenesis through indirect mTOR signaling modulation. Because TAK 165 showed obviously different effects on Alcian blue staining (Figure 4C), BMP and TGF-β signaling (Figures 7A and 7B), and mTOR signaling compared with other HTS hits (Figures 6D–6F and S6), TAK 165 might be useful for future concurrent treatment with other direct inhibitors. A detailed mechanism of action and the identification of direct targets of TAK 165 remain important issues awaiting future clarification.

To identify potential mechanisms that suppress the enhanced chondrogenesis of FOP, we developed an HTS system that focuses on the constitutive activity of FOP-ACVR1. Although we previously focused on activin A-triggered enhanced chondrogenesis, inspired by the recent report showing that a substantial number of FOP patients experience the progression of HO without apparent flares (Pignolo et al., 2016), we adopted the constitutive activity of FOP-ACVR1 for phenotypic screening system. We screened a library of about 5,000 small-molecule compounds and finally identified hit compounds that were effective in multiple HO model mice (Figures 5, 6, and S3). However, although effective, the effect of the hit compounds had high variability. Improving *in vivo* models will reduce variation caused by the incomplete purity of the mouse strain (Figure 5) or by the technical challenges of the transplantation assay (Figure 6). Another important issue is how to enhance the efficacy of our compounds *in vivo*. Since the hit compounds are prototypes or lead compounds, the solubility and pharmacokinetics might be not well studied, hampering assessment of the maximal dose that suppresses HO.

There are two types of approaches in FOP drug discovery. The first approach is target-based and focuses on FOP-ACVR1 itself, e.g., kinase inhibition of FOP-ACVR1 or downregulation of *Acvr1* expression (Cappato et al., 2016, Engers et al., 2013, Hamasaki et al., 2012, Hao et al., 2010, Mohedas et al., 2013, Sanvitale et al., 2013, Yu et al., 2008). The second approach is phenotypic screening and focuses on HO-related phenotypes, for example, enhanced chondrogenesis, osteogenesis, and so forth (Shore and Kaplan, 2010). The former approach is quite logical and promising because it suppresses causal genes in FOP, but in general highly selective kinase inhibition is extremely challenging (Anastassiadis et al., 2011). On the other hand, phenotypic screening could highlight the most effective and/or novel mechanism that underlies FOP pathology, although the challenge here is to develop a robust system that screens compounds or gives further validation of candidates (Hino et al., 2017, Moffat et al., 2017). In this study, we performed HTS of inhibitors for ALP activity triggered by the constitutive activity of FOP-ACVR1 in the chondrogenic cell line ATDC5. Since ALP is a well-validated prehypertrophic chondrogenic marker, our HTS platform is a successful example of phenotypic screening for FOP. Consequently, we identified TAK 165, an mTOR signaling modulator that indirectly inhibits mTOR signaling, in addition to two direct ACVR1 kinase inhibitors (AZD0530 and PD 161570). Thus, phenotypic screening could contribute to understanding FOP pathophysiology.

In FOP patients, two phases, inflammation and the destruction of connective tissues (phase 1) and bone formation (phase 2), were proposed in the progression of HO (Shore and Kaplan, 2010), and each phenotype is a potential target for intervention. The suppression of phase 1 by anti-inflammatory drugs such as oral corticosteroids shows limited effects on FOP patients, but other approaches such as mast cell inhibitors might become new drug candidates (Brennan et al., 2017, Convente et al., 2017), although a future clinical trial is needed to prove the efficacy and side effects in FOP patients. Phase 2 can be further subdivided into three stages: fibroproliferation and angiogenesis (2A), chondrogenesis (2B), and osteogenesis (2C). We have focused on stage 2B, chondrogenesis, in both this and a previous study (Hino et al., 2017), because we assumed that the inhibition of chondrogenesis might not cause serious side effects since little or no chondrogenesis occurs in adults (Falah et al., 2010). On the other hand, as bone remodeling is a lifelong process (Maggioli and Stagi, 2017), the inhibition of stage 2C (osteogenesis) might evoke adverse effects, such as fracture or osteoporosis, regardless of any HO suppression. Although stage 2A (enhanced fibroproliferation) is often observed in the HO of FOP patients, no defined molecules have been reported for this process. Thus, a phenotypic screening focused on fibroproliferation could shed light on novel mechanisms of FOP. Three studies have identified the cell of origin of HO in FOP model mice (Agarwal et al., 2017, Dey et al., 2016, Lees-Shepard et al., 2018), and iMSCs or paraxial mesoderm-derived MSC-like cells can be induced from FOP-iPSCs (Hino et al., 2015, Hino et al., 2017, Matsumoto et al., 2015, Nakajima et al., 2018); therefore, these cells could be applicable to future phenotypic screenings for the inhibition of stage 2A. Combination therapy targeting multiple phases could be the best strategy for controlling the HO of FOP.

## Experimental Procedures

Full experimental procedures and associated references are available in Supplemental Experimental Procedures.

### Study Approval

All experimental protocols dealing with human subjects were approved by the Ethics Committee of the Department of Medicine and Graduate School of Medicine, Kyoto University. Written informed consent was provided by each donor. All animal experiments were approved by the institutional animal committee of Kyoto University.

### Chemicals Libraries

All chemical libraries were purchased from the suppliers listed in Supplemental Experimental Procedures. Almost all compounds were bioactive and/or annotated.

### Cell Culture

ATDC5 cells were maintained in DMEM/F-12 (Thermo Fisher Scientific) supplemented with 5% (v/v) FBS (Nichirei). The FOP-iPSCs used in this study (previously described as vFOP4-1 [Matsumoto et al., 2013]) harbor the R206H heterozygous mutation in ACVR1, and gene-corrected resFOP-iPSCs were generated by BAC-based homologous recombination. These cells fulfilled several criteria for iPSCs including the expression of pluripotent markers, teratoma formation, normal karyotype, and morphology. Growth and gene expression profiles of the resFOP-iPSC clones were indistinguishable from the original FOP-iPSCs (Matsumoto et al., 2015); however, remarkably distinct responsiveness to activin A was observed (Hino et al., 2015).

### ALP Assay

ATDC5/FOP-ACVR1 cells were plated in 96-well white plates (2,000 cells/well/40 μL, Corning) in DMEM/F-12 supplemented with 5% (v/v) FBS. Two hours after incubation at 37°C under 5% CO_2_, 10 μL of test compounds (final 1 μM) was added, and the assay plates were incubated at 37°C under 5% CO_2_. After 3 days of incubation, ALP activity was measured using an Amplite Colorimetric Alkaline Phosphatase Assay Kit (AAT Bioquest) according to the manufacturer's protocol.

### 2D Chondrogenic Induction

Chondrogenic induction was performed, and differentiation properties were assayed as previously described (Hino et al., 2015, Nasu et al., 2013, Umeda et al., 2012).

### *In Vivo* Experiments

hFOP-ACVR1 conditional transgenic mice (Beard et al., 2006, Hino et al., 2017, Ohnishi et al., 2014, Yamada et al., 2013), BMP-7-induced HO model mice (Hino et al., 2017), and activin A-induced HO model mice transplanted with FOP-iMSCs (Hino et al., 2017) were intraperitoneally administered 5 mg/kg AZD0530, TAK 165, or rapamycin (once daily, five times a week) and analyzed as previously described (Hino et al., 2017).

### Statistics

The statistical significance of all experiments was calculated by Prism 6 (GraphPad Software). p values less than 0.05 were considered statistically significant.

## Author Contributions

Conceptualization and Project Administration, K. Hino and M.I.; Investigation, K. Hino, C.Z., K. Horigome, M.N., Y.O., S.N., S.K., and A.O.; Formal Analysis, K. Hino, C.Z., K. Horigome, M.N., and A.O.; Resources, K. Horigome and Y.Y.; Writing – Original Draft, K. Hino, J.T., and M.I. All authors read and approved the final manuscript.

## Declaration of Interests

K. Hino and K. Horigome are employees of Sumitomo Dainippon Pharma Co. Ltd. M.I. and J.T. are supported by a research fund from Sumitomo Dainippon Pharma Co. Ltd. The other authors declare no competing interests.

## Accession Numbers

Microarray data were deposited in the GEO of NCBI under the accession number GEO: GSE108771.

## Supplemental Information

Document S1. Supplemental Experimental Procedures and Figures S1–S7

Document S2. Article plus Supplemental Information
