# Supplementary material for: An mTOR Signaling Modulator Suppressed Heterotopic Ossification of Fibrodysplasia Ossificans Progressiva
Source: Stem Cell Reports. 2018 Nov 1;11(5):1106–19. doi: 10.1016/j.stemcr.2018.10.007 (PMC6235670; doi:10.1016/j.stemcr.2018.10.007)
Supplement: Document S1. Supplemental Experimental Procedures and Figures S1–S7 [file mmc1.pdf]

Stem Cell Reports, Volume *11*

Supplemental Information

An mTOR Signaling Modulator Suppressed Heterotopic Ossification of Fibrodysplasia Ossificans Progressiva

Kyosuke Hino, Chengzhu Zhao, Kazuhiko Horigome, Megumi Nishio, Yasue Okanishi, Sanae Nagata, Shingo Komura, Yasuhiro Yamada, Junya Toguchida, Akira Ohta, and Makoto Ikeya

SUPPLEMENTAL EXPERIMENTAL PROCEDURES

Study approval. All experimental protocols dealing with human subjects were approved by the Ethics Committee of the Department of Medicine and Graduate School of Medicine, Kyoto University. Written informed consent was provided by each donor. All animal experiments were approved by the institutional animal committee of Kyoto University.

Cell culture. ATDC5 cells were maintained in DMEM/F-12 (Thermo Fisher Scientific) supplemented with 5% (v/v) FBS (Nichirei). iPSCs were maintained in primate ES cell medium (ReproCELL) supplemented with 4 ng/mL recombinant human FGF2 (Wako Pure Chemical). To induce induced neural crest cells (iNCCs), mTeSR1 medium (STEMCELL Technology) was used for the feeder-free culture of iPSCs. The induction and maintenance of iNCCs and iMSCs derived from iPSCs were previously described (Fukuta et al., 2014; Matsumoto et al., 2015). Briefly, iNCCs were induced in chemically defined medium (CDM) supplemented with 10 µM SB-431542 and 1 µM CHIR99021 for 7 days. iNCCs were maintained in CDM supplemented with 10 µM SB-431542, 20 ng/mL FGF2 and 20 ng/mL recombinant human EGF (R&D Systems) for up to 20 passages. iMSCs were induced and maintained in αMEM (Thermo Fisher Scientific) supplemented with 10% (v/v) FBS (Nichirei), 5 ng/mL FGF2 and 0.5% penicillin and streptomycin (Thermo Fisher Scientific). The FOP-iPSCs used in this study (previously described as vFOP4-1 (Matsumoto et al., 2013)) harbor the R206H heterozygous mutation in ACVR1, and gene-corrected resFOP-iPSCs were generated by BAC-based homologous recombination. These cells fulfilled several criteria for iPSCs including the expression of pluripotent markers, teratoma formation, normal karyotype and morphology. Growth and gene expression profiles of the resFOP-iPSC clones were indistinguishable from the original FOP-iPSCs (Matsumoto et al., 2015), however, remarkably distinct responsiveness to Activin-A was observed (Hino et al., 2015). C3H10T1/2 (murine multipotent mesenchymal cells)-expressing Dox-inducible hINHBA (C3H-DoxOn-hINHBA) was maintained in DMEM（Nacalai Tesque) supplemented with 10% FBS and 1 mM Na-pyruvate (Thermo Fisher Scientific) and used for an Activin-A-induced HO model transplanted with FOP-iMSCs as previously reported (Hino et al., 2015). In the wound healing assay, healthy control iPSC (414C2)-derived iMSCs were used (Okita et al., 2011).

Reagents. Activin-A, BMP-4, BMP-7 and TGF-β3 were purchased from R&D Systems. Rapamycin was purchased from MedChemexpress. SB-431542 was purchased from Sigma-Aldrich. TAK 165, PD 161570, CP-724714 and Lapatinib were purchased from Selleck Chemicals. DMH-1 was purchased from Tocris Bioscience. AZD0530 was purchased from ANgene. Trastuzumab and Pertuzumab were purchased from BioVision or Creative-Biolabs, respectively. Activin-A, BMP-7 and TGF-β3 were dissolved according to the manufacturer's protocols and used at 100 ng/mL (Activin-A and BMP-7) or 10 ng/mL (BMP-4 and TGF-β3) unless otherwise noted.

Chemicals Libraries. All chemical libraries were purchased from the suppliers listed. Almost all compounds were bioactive and/or annotated.

Library Name	Supplier	Number
Microsource International Drugs	MicroSource Discovery Systems	238
Microsource US Drugs		1020
Enzo FDA	Enzo Life Sciences	636
Enzo ICCB		474
Enzo kinase inhibitors		76
LOPAC1280	Sigma-Aldrich	1280
Sigma Pfizer		74
Myriascreen	TimTec	72
EMD Kinase Inhibitors	Merck Millipore	244
Selleck kinase inhibitors	Selleck Chemicals	141
Tocrs Mini Selected for CiRA	Tocris Bioscience	637
Total		4892

Generation of ATDC5 stably expressing ACVR1. WT- or FOP-ACVR1 was inserted into doxycycline (Dox)-inducible vector KW111 (Woltjen et al., 2009), which enables us to easily produce stably expressing cell lines utilizing the *piggyBac* (PB) transposon system from the cabbage looper moth *Trichoplusia ni* (Ding et al., 2005) (KW111/WT-ACVR1 or KW111/FOP-ACVR1). KW111/WT-ACVR1 or KW111/FOP-ACVR1 and PBaseII plasmid (PB transposase expression vector) (Matsui et al., 2014) were co-transfected into ATDC5 cells by FuGeneHD® (Promega) according to the manufacturer's protocol, and the neomycin-resistant population (500 µg/mL) was selected. Further selection was performed by selecting a mCherry high positive population (< 5%) after six hours of Dox treatment, sorting it with fluorescent-activated cell sorting (FACS) using AriaII (BD) according to the manufacturer's protocol, and designating it as ATDC5/WT-ACVR1 or ATDC5/FOP-ACVR1. KW111 included PB, active transposable PB elements; TRE-mCMVP, Tetracycline Response Element and a minimal CMV promoter; rtTA, reverse tetracycline-controlled transactivator. PBaseII was an expression plasmid vector containing the PB transposase cDNA with optimized codon usage to human under the control of the CAG promoter (Matsui et al., 2014).

HTS campaign and follow-up screens. ATDC5/FOP-ACVR1 cells were plated in 96-well white plates (2,000 cells/well/40 µL, Corning) in DMEM/F-12 supplemented with 5% (v/v) FBS. Two hours after incubation at 37 °C under 5% CO_2, 10 µL of test compounds (final 1 µM) was added, and assay plates were incubated at 37 °C under 5% CO_2. After 3 days incubation, Alkaline Phosphatase activity (ALP) or AlamarBlue activity was measured using Amplite™ Colorimetric Alkaline Phosphatase Assay Kit (AAT Bioquest) or alamarBlue® Cell Viability Reagent (Thermo Fisher Scientific) according to the manufacturer's protocol, respectively. The absorbance at 400 nm (Abs for ALP) or Relative Fluorescent Unit of Ex560/Em590 nm (RFU for AlamarBlue) was measured on POWERSCAN4 (DS Pharma Biomedical) or EnVision® Multilabel Reader (PerkinElmer). Inhibitory effects of the screened compounds are given as percent inhibition, which was calculated using the following equation: (1-([Abs of compound] - [Abs of Min])/([Abs of Max] - [Abs of Min]))× 100, where [Abs of Max] and [Abs of Min] are the mean Abs of DMSO control and 1 µM DMH-1, respectively. The Z' factor, which is widely used as a measure of the assay quality of each plate, was calculated using the following equation: Z' factor = 1 - 3 × ([SD of Max] + [SD of Min])/([Abs of Max] - [Abs of Min]) (SI Appendix, Fig. S3A). The average Z' factor in the HTS assay was 0.74, indicating accuracy and reliability of the HTS campaign (general criteria of HTS > 0.50) (Zhang et al., 1999). The S/B (signal-to-background) ratio was calculated using the following equation: ([Abs of Max]/[Abs of Min]) (SI Appendix, Fig. S3B). The average S/B ratio in the HTS assay was 6.9, also indicating accuracy and reliability of the HTS campaign (general criteria of HTS > 3.0).

Using ATDC5/FOP-ACVR1 cells, we performed a first screening (n=2; test compounds = 1 µM, Fig. 2A and B) against 4,892 small molecule compounds. In the first screening, 160 hit compounds satisfied the criteria more than 40% inhibition of ALP activity against DMSO control cells, less than 40% inhibition of viability, and margin (Inhibition of ALP activity (%) - Inhibition of viability (%)) more than 20%. A second screening was performed against the above 160 compounds (n=2; test compounds = 0.1, 0.3, 1, 3 µM), and 79 hit compounds satisfied the criteria 40% inhibition of ALP activity against DMSO control cells and margin more than 50% at any dose (SI Appendix, Fig. S4 and S5). To explore compounds which have the potential to identify new mechanisms or contribute to future drug repositioning, we selected 14 compounds, and detailed concentration-dependent assays were performed (Fig. 2E and SI Appendix, Fig. S6). As a result, we identified 7 compounds which showed stronger IC50 (< 500 nM) and less toxicity (Viability @ 10 µM > 50%) through the HTS campaign that focused on the constitutive activity of FOP-ACVR1 (Fig. 2E, red).

2D chondrogenic induction. Chondrogenic induction was performed, and differentiation properties were assayed as previously described (Hino et al., 2015; Nasu et al., 2013; Umeda et al., 2012). Briefly, iMSCs (1.5 × 10^5) were cultured in fibronectin-coated 24-well plates (BD Biosciences) using the chondrogenic medium with 100 ng/mL Activin-A and inhibitors until day 7 unless otherwise noted. Gene-specific siRNAs were purchased from Thermo Fisher Scientific (Silencer® Select Pre-designed siRNA). For the transient expression of siRNA, Lipofectamine® RNAiMAX Reagent (Thermo Fisher Scientific) was used according to the manufacturer's instructions. Knockdown efficiencies (n=1) of MTOR and ERBB2 16 h after transfection in FOP-iMSCs were 23.8% and 21.1%, respectively.

siRNA sequences.

Gene	ID	Sense	Antisense
MTOR	s604	GGAGCCUUGUUGAUCCUUAtt	UAAGGAUCAACAAGGCUCCat
ERBB2	s611	GCUCAUCGCUCACAACCAAtt	UUGGUUGUGAGCGAUGAGCac

Cardiotoxin-induced HO model in human FOP-ACVR1 conditional transgenic mice. Female hFOP-ACVR1 conditional transgenic mice (Beard et al., 2006; Hino et al., 2017; Ohnishi et al., 2014; Yamada et al., 2013) (f5-6 offspring of chimeric mice, age- and body weight-matched between groups) were used between 16 and 20 weeks of age. Mice were administered 2 mg/mL Dox in their drinking water supplemented with 10 mg/mL sucrose to induce FOP-ACVR1. Cardiotoxin (9.1 µg/mouse, Latoxan) was injected into the right gastrocnemius muscle to initiate skeletal muscle injury and subsequent heterotopic bone formation (Chakkalakal et al., 2012). Test compounds (16% DMSO in 0.5 w/v% Methyl Cellulose 400) were intraperitoneally administered once a day, five times a week. Mice were analyzed three weeks after injection. For X-ray images, mice were anesthetized with isoflurane (5% for induction, 2-3% for maintenance, Abbvie), immobilized and X-rayed using µFX-1000 (Fujifilm) or DX-50 (Faxitron Bioptics). µCT images were scanned using X-ray CT systems (inspeXio SMX-100CT, Shimadzu) and analyzed with TRI/3D-BON software (Ratoc System Engineering) according to the manufacturer's instructions. Four weeks after injection, the injected sites were harvested, fixed with 4% paraformaldehyde for 24 h, embedded in paraffin, and sectioned and stained with HE, von Kossa, Safranin O and anti-Collagen I antibody as previously described (Hino et al., 2015; Umeda et al., 2012).

BMP-7-induced HO model mice. BMP-7 (2 µg/mouse) was injected into the right gastrocnemius muscle of C57BL/6NJcl mice (6-8 weeks, male, CLEA Japan), and compounds were administered once a day, five times a week (intraperitoneal administration). Mice were analyzed 2 weeks after injection.

Activin-A-induced HO model transplanted with FOP-iMSCs. FOP- (right leg) and resFOP-iMSCs (left leg) (4×10^6 cells, respectively) were transplanted into the gastrocnemius muscle of NOD/ShiJic-scid Jcl (NOD/SCID) mice (8 weeks, male, CLEA Japan) with C3H-DoxOn-hINHBA (5×10^5 cells), which can continuously expose Activin-A to the transplanted iMSCs in vivo (Hino et al., 2015). In the Dox-induced group, 1 mg/mL Dox (Sigma-Aldrich) was administered via drinking water with 10 mg/mL sucrose (Nacalai Tesque) for two weeks after transplantation. Compounds were intraperitoneally administered once a day, five times a week. Eight weeks after transplantation, the transplanted cells were harvested, fixed with 4% paraformaldehyde for 24 h, embedded in paraffin, and sectioned and stained with HE, von Kossa, Safranin O, human specific anti-Vimentin antibody and Collagen I antibody as previously described (Hino et al., 2015; Umeda et al., 2012; Yamashita et al., 2015).

Histological analysis of growth plates chondrocytes. Knee joints were obtained from BMP-7-injected and test compounds-administrated C57BL/6NJcl mice after 2 weeks treatment and fixed, paraffin-embedded, and sectioned and stained with Safranin O. The thickness of the proliferative and hypertrophic zones of the growth plate were measured using ImageJ software on images obtained using a Keyence microscope (Keyence America). The proliferative zone was defined as the region with flat chondrocytes stacked in longitudinal columns, and the hypertrophic zone as the region where chondrocytes are enlarged in size and form clusters.

Normal bone µCT. The left legs of mice were obtained from BMP-7-injected and test-compounds administrated C57BL/6NJcl mice after 2 weeks treatment. The region of interest (ROI) included the femur-tibia (entire femur in addition to the part of tibia above tibia/fibular junction).

Wound healing assay. Healthy control iPSC (414C2)-derived iMSCs (5×10^4) were cultured in 24-well plates using αMEM supplemented with 10% (v/v) FBS, 5 ng/mL FGF2 and 0.5% penicillin and streptomycin at 37 °C for 72 h to form a confluent monolayer. Gaps were created by scratching the plates with a sterile pipette tip (1000 µL). The cells were then washed with PBS to remove the detached cells and replaced with medium containing 1% (v/v) FBS in the presence of DMSO (control) or test compounds (10 nM Rapamycin or 1 µM TAK165). The cell culture plates were transferred to a Biostation CT (Nikon Corp., Tokyo, Japan) programmed to take photographs at t = 0 h and t = 24 h. The migrated area was determined by subtracting the wound area at

time point t = 24 h from t = 0 h, and relative cell migration was expressed as the ratio of the absolute cell migrations between the experimental and control groups by using CL-Quant software (Nikon Corp.).

Quantitative PCR analysis. Total RNA was purified with RNeasy Kit (Qiagen) and treated with DNase-one Kit (Qiagen) to remove genomic DNA. Total RNA (0.3 μg) was reverse transcribed for single-stranded cDNA using random primers and Superscript III reverse transcriptase (Thermo Fisher Scientific) according to the manufacturer's instructions. Quantitative PCR was performed with Thunderbird SYBR qPCR Mix (TOYOBO) and analyzed with the StepOne real-time PCR system (Applied Biosystems). All data (relative expression) were corrected by β-actin.

Primers for RT-qPCR.

Gene	Forward	Reverse
ACTB	CACCATTGGCAATGAGCGGTTC	AGGTCTTTGCGGATGTCCACGT
MTOR	GACGAGAGATCATCCGCCAG	ACAAGGGACCGCACCATAAG
ERBB2	GCTCCTCCTCGCCCTCTT	TGAGTTCCAGGTTTCCCTGC

Microarray experiments. 2D chondrogenic induction was performed in FOP-iMSCs stimulated with or without 100 ng/mL Activin-A, TAK 165, CP-724714 and Lapatinib. Seven days after incubation, mRNA was extracted. RNA was reverse transcribed, biotin-labeled and hybridized to GeneChip Human Gene 1.0 ST Expression Array, which was subsequently washed and scanned according to the manufacturer's instructions (Affymetrix). Raw CEL files were imported into GeneSpring GX 12.6.1 software (Agilent Technologies), and expression values were calculated with the RMA16 algorithm. Pathway analysis was performed by Ingenuity pathway analysis (Qiagen).

Western blotting. SDS-PAGE and blotting with whole-cell lysates were performed by standard procedures. Protein bands were detected with ECL Prime Western Blotting Detection Reagent (GE Healthcare) and visualized using BIO-RAD Molecular Imager® Chemi-Doc™ XRS+ with Image Lab™ software (Bio-Rad). All data (relative intensity) were corrected by β-actin.

Antibodies for western blotting and immunostaining.

	Name	Company	Cat. No	Concentration
1st antibody	Human Activin RIA/ALK-2 Antibody	R&D Systems)	AF637	1:100
	Phospho-S6 Ribosomal Protein (Ser235/236) Antibody	Cell Signaling Technology	#2211	1:1000
	Monoclonal Anti-b-Actin-Peroxidase clone AC-15	SIGMA-ALDRICH	A3854	1:200000
	Anti-human Vimentin antibody	Abcam	ab16700	1:40
	Collagen I Antibody	Novus Biologicals	NB600-408	1:100
2nd antibody	Anti-rabbit IgG, HRP-linked Antibody	Cell Signaling Technology	#7074	1:10000
	Goat anti-Rabbit IgG (H+L) Secondary Antibody, Alexa Fluor® 488 conjugate	Invitrogen	A-11008	1:100
	Goat anti-Rabbit IgG (H+L) Secondary Antibody, Alexa Fluor® 555 conjugate	Invitrogen	A-21428	1:500

GAG value. The GAG content was quantified in pellets with the Blyscan Glycosaminoglycan Assay Kit (Biocolor). The DNA content was quantified using PicoGreen dsDNA Quantitation Kit (Thermo Fisher Scientific).

Immunohistochemistry. Paraffin-embedded sections were deparaffinized. For human specific anti-Vimentin antibody, antigen retrieval was performed by autoclave (105 °C, 10 min). Samples were blocked with Blocking One (Nacalai Tesque) for 60 min and then incubated with human specific anti-Vimentin antibody (Abcam) or

anti-Collagen I antibody (Novus Biologicals) diluted in Can Get Signal® immunostain solution B (TOYOBO) for 16-18 h at 4 °C. Next, samples were washed several times in 0.2% tween20 (Sigma-Aldrich) in PBS and incubated with Goat anti-Rabbit IgG (H+L) secondary antibody, Alexa Fluor® 488 or 555 conjugate (Thermo Fisher Scientific) diluted in Can Get Signal® immunostain solution B for one hour at room temperature. DAPI (10 μg/mL) was used to counterstain nuclei. Samples were observed by BZ-9000E (KEYENCE).

Statistics. The statistical significance of all experiments was calculated by Prism 6 (GraphPad Software). P values less than 0.05 were considered statistically significant.

SUPPLEMENTAL REFERENCES

Beard, C., Hochedlinger, K., Plath, K., Wutz, A., and Jaenisch, R. (2006). Efficient method to generate single-copy transgenic mice by site-specific integration in embryonic stem cells. Genesis *44*, 23-28.

Chakkalakal, S.A., Zhang, D., Culbert, A.L., Convente, M.R., Caron, R.J., Wright, A.C., Maidment, A.D., Kaplan, F.S., and Shore, E.M. (2012). An Acvr1 R206H knock-in mouse has fibrodysplasia ossificans progressiva. J Bone Miner Res *27*, 1746-1756.

Ding, S., Wu, X., Li, G., Han, M., Zhuang, Y., and Xu, T. (2005). Efficient transposition of the piggyBac (PB) transposon in mammalian cells and mice. Cell *122*, 473-483.

Fukuta, M., Nakai, Y., Kirino, K., Nakagawa, M., Sekiguchi, K., Nagata, S., Matsumoto, Y., Yamamoto, T., Umeda, K., Heike, T., *et al.* (2014). Derivation of Mesenchymal Stromal Cells from Pluripotent Stem Cells through a Neural Crest Lineage using Small Molecule Compounds with Defined Media. PloS one *9*, e112291.

Hino, K., Horigome, K., Nishio, M., Komura, S., Nagata, S., Zhao, C., Jin, Y., Kawakami, K., Yamada, Y., Ohta, A., *et al.* (2017). Activin-A enhances mTOR signaling to promote aberrant chondrogenesis in fibrodysplasia ossificans progressiva. J Clin Invest *127*, 3339-3352.

Hino, K., Ikeya, M., Horigome, K., Matsumoto, Y., Ebise, H., Nishio, M., Sekiguchi, K., Shibata, M., Nagata, S., Matsuda, S., *et al.* (2015). Neofunction of ACVR1 in fibrodysplasia ossificans progressiva. Proceedings of the National Academy of Sciences of the United States of America *112*, 15438-15443.

Matsui, H., Fujimoto, N., Sasakawa, N., Ohinata, Y., Shima, M., Yamanaka, S., Sugimoto, M., and Hotta, A. (2014). Delivery of full-length factor VIII using a piggyBac transposon vector to correct a mouse model of hemophilia A. PloS one *9*, e104957.

Matsumoto, Y., Hayashi, Y., Schlieve, C.R., Ikeya, M., Kim, H., Nguyen, T.D., Sami, S., Baba, S., Barruet, E., Nasu, A., *et al.* (2013). Induced pluripotent stem cells from patients with human fibrodysplasia ossificans progressiva show increased mineralization and cartilage formation. Orphanet J Rare Dis *8*, 190.

Matsumoto, Y., Ikeya, M., Hino, K., Horigome, K., Fukuta, M., Watanabe, M., Nagata, S., Yamamoto, T., Otsuka, T., and Toguchida, J. (2015). New Protocol to Optimize iPS Cells for Genome Analysis of Fibrodysplasia Ossificans Progressiva. Stem Cells *33*, 1730-1742.

Nasu, A., Ikeya, M., Yamamoto, T., Watanabe, A., Jin, Y., Matsumoto, Y., Hayakawa, K., Amano, N., Sato, S., Osafune, K., *et al.* (2013). Genetically matched human iPS cells reveal that propensity for cartilage and bone differentiation differs with clones, not cell type of origin. PloS one *8*, e53771.

Ohnishi, K., Semi, K., Yamamoto, T., Shimizu, M., Tanaka, A., Mitsunaga, K., Okita, K., Osafune, K., Arioka, Y., Maeda, T., *et al.* (2014). Premature termination of reprogramming in vivo leads to cancer development through altered epigenetic regulation. Cell *156*, 663-677.

Okita, K., Matsumura, Y., Sato, Y., Okada, A., Morizane, A., Okamoto, S., Hong, H., Nakagawa, M., Tanabe, K., Tezuka, K., *et al.* (2011). A more efficient method to generate integration-free human iPS cells. Nature methods *8*, 409-412.

Umeda, K., Zhao, J., Simmons, P., Stanley, E., Elefanty, A., and Nakayama, N. (2012). Human chondrogenic paraxial mesoderm, directed specification and prospective isolation from pluripotent stem cells. Scientific reports *2*, 455.

Woltjen, K., Michael, I.P., Mohseni, P., Desai, R., Mileikovsky, M., Hamalainen, R., Cowling, R., Wang, W., Liu, P., Gertsenstein, M., *et al.* (2009). piggyBac transposition reprograms fibroblasts to induced pluripotent stem cells. Nature *458*, 766-770.
Yamada, K., Ohno, T., Aoki, H., Semi, K., Watanabe, A., Moritake, H., Shiozawa, S., Kunisada, T., Kobayashi, Y., Toguchida, J., *et al.* (2013). EWS/ATF1 expression induces sarcomas from neural crest-derived cells in mice. J Clin Invest *123*, 600-610.
Yamashita, A., Morioka, M., Yahara, Y., Okada, M., Kobayashi, T., Kuriyama, S., Matsuda, S., and Tsumaki, N. (2015). Generation of scaffoldless hyaline cartilaginous tissue from human iPSCs. Stem Cell Reports *4*, 404-418.
Zhang, J.H., Chung, T.D., and Oldenburg, K.R. (1999). A Simple Statistical Parameter for Use in Evaluation and Validation of High Throughput Screening Assays. Journal of biomolecular screening *4*, 67-73.

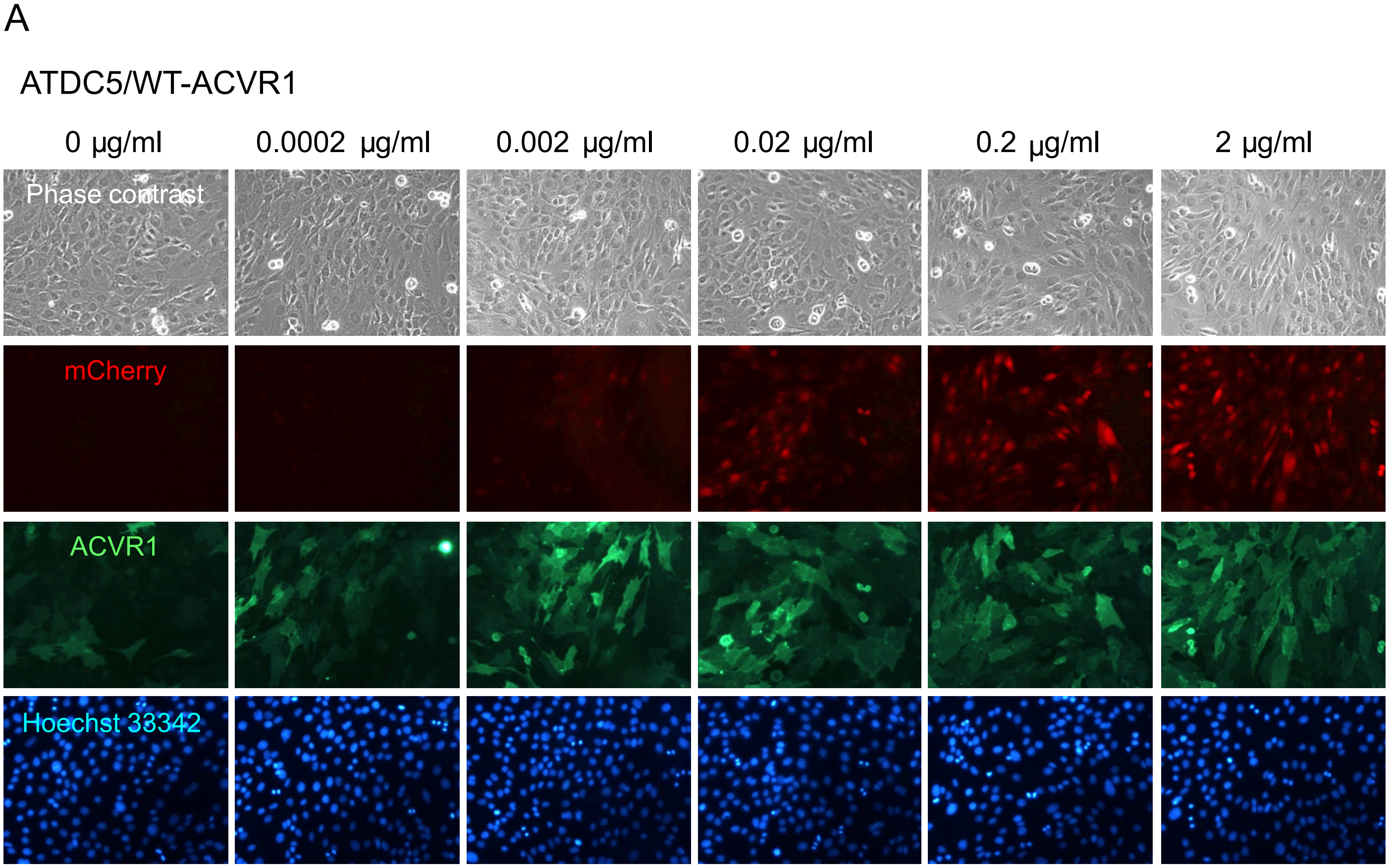

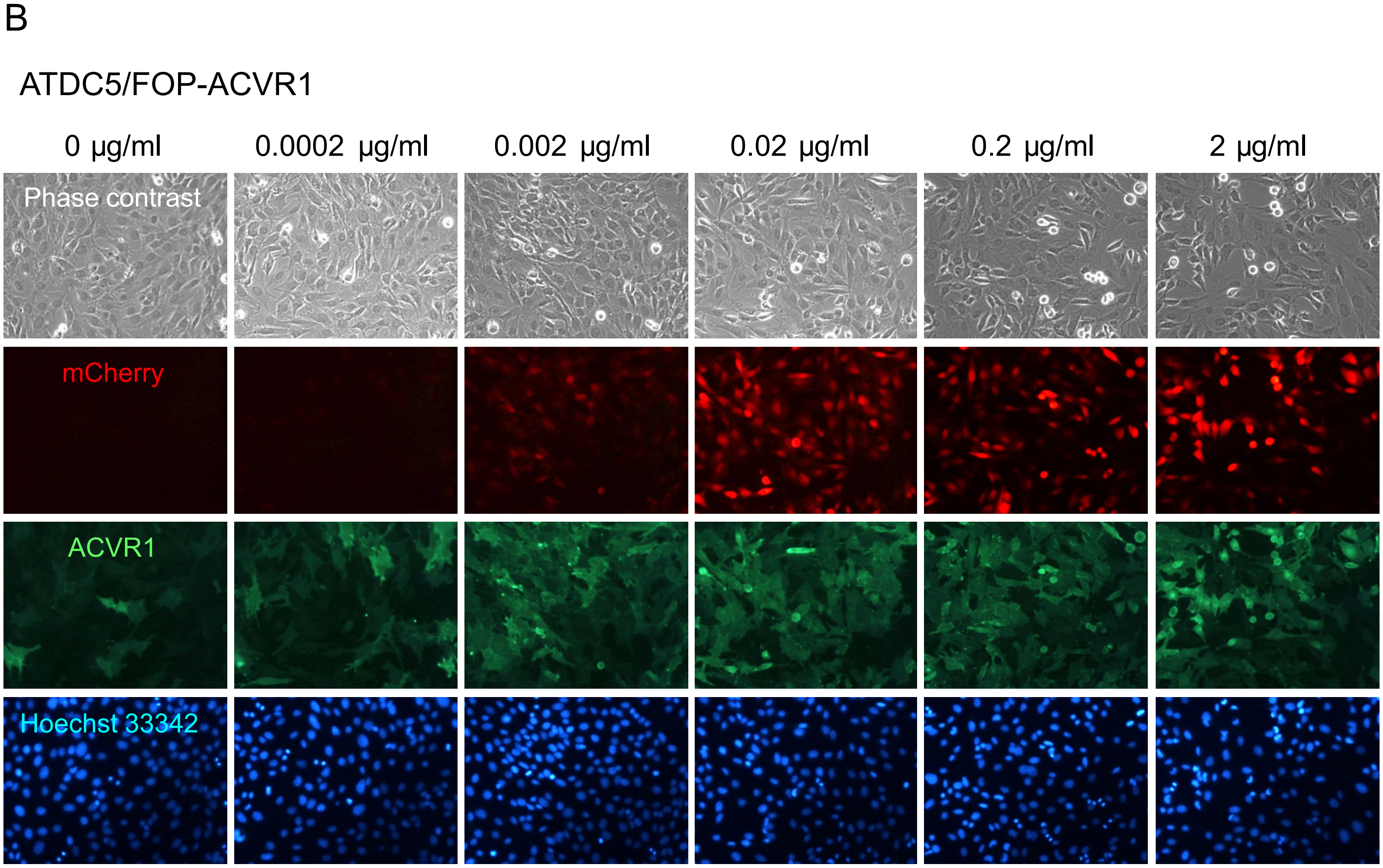

Figure S1. Expression of ACVR1 and mCherry in ATDC5/WT-ACVR1 or ATDC5/FOP-ACVR1. Related to Figure 1. 24 h after Dox treatment (0-2 µg/mL), cells were fixed and stained. Panels of DOX 0 and 0.002 µg/mL condition are same as Figure 1B (B). Scale bars, 100 µm.

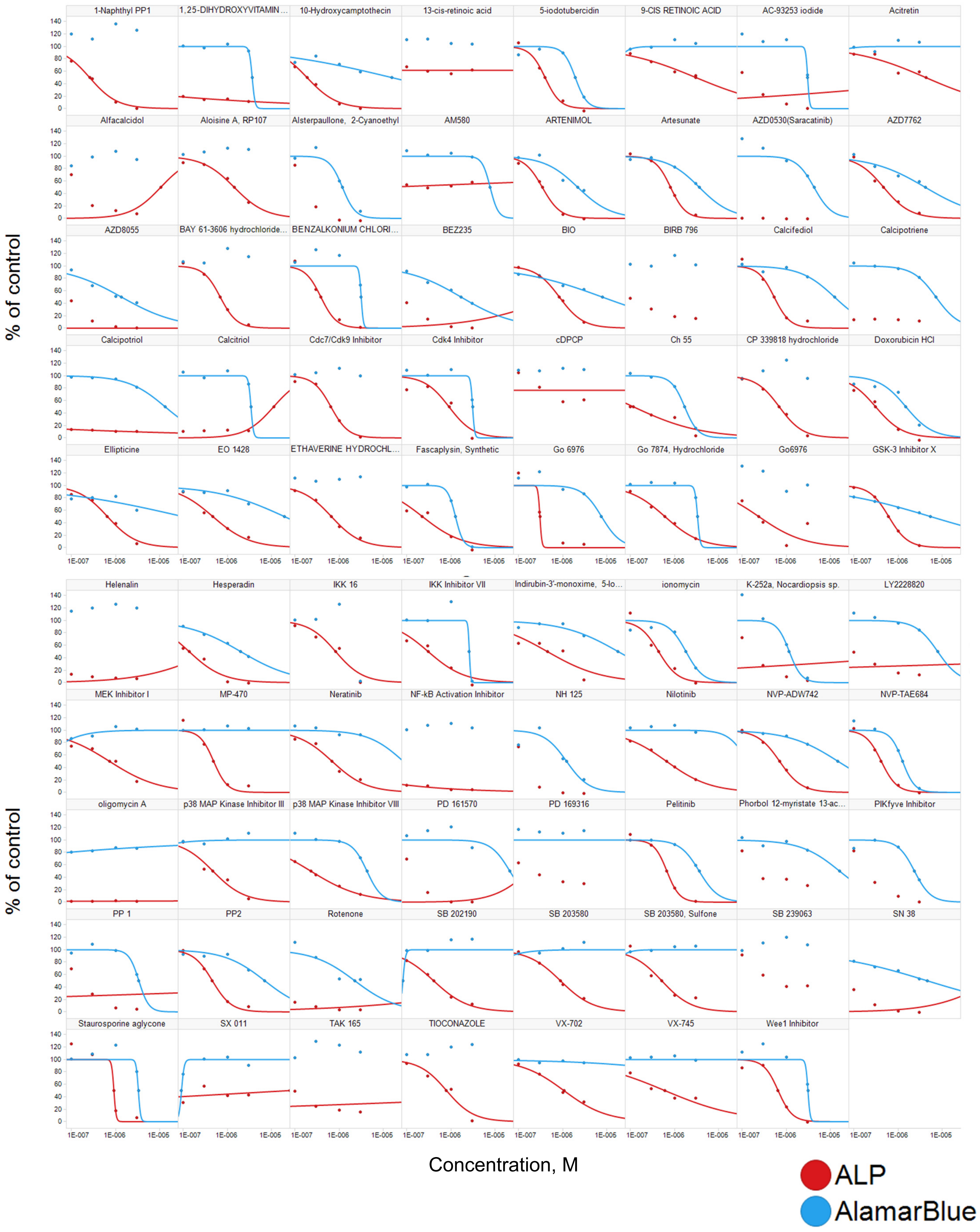

Figure S2. Dose-dependent assay results of 79 hit compounds. Related to Figure 2. ALP assay and AlamarBlue assay were performed using the same protocol as the HTS. Results are the mean of biological duplicate. 0.1, 0.3, 1, 3 μM of test compounds were assessed. Listed compounds showed more than 40% inhibition of ALP activity against DMSO control cells and margin more than 50% (inhibition of ALP activity (%) - inhibition of viability (%)) at any dose.

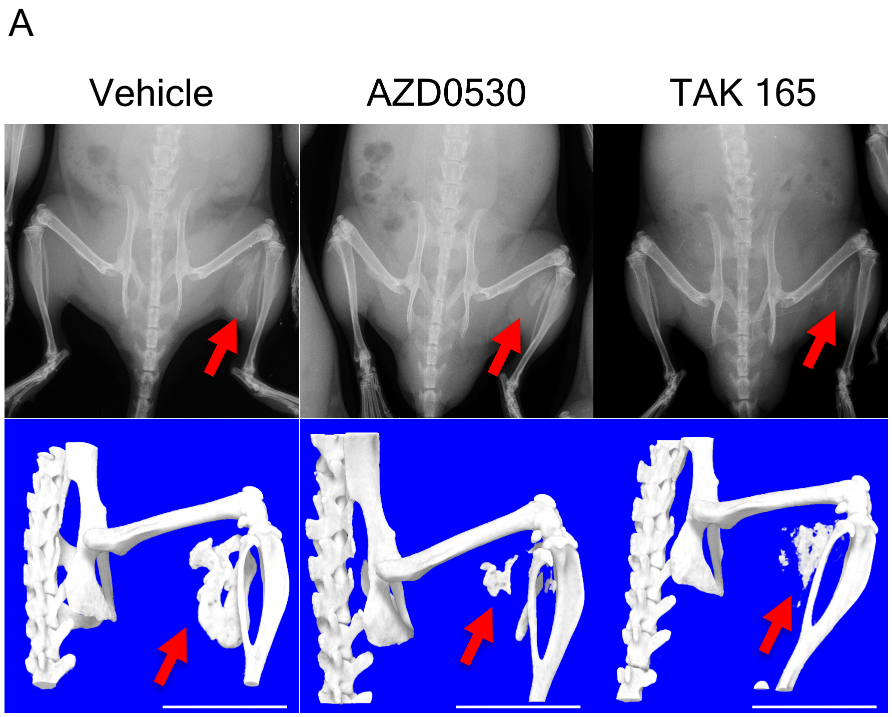

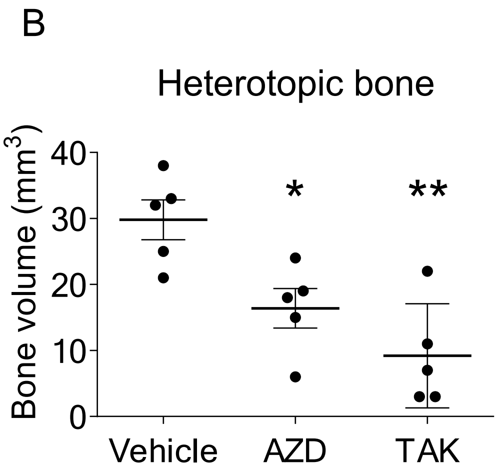

C

Compound	# of HO (> 20 mm^3)
Vehicle	5/5
AZD0530	1/5
TAK 165	1/5

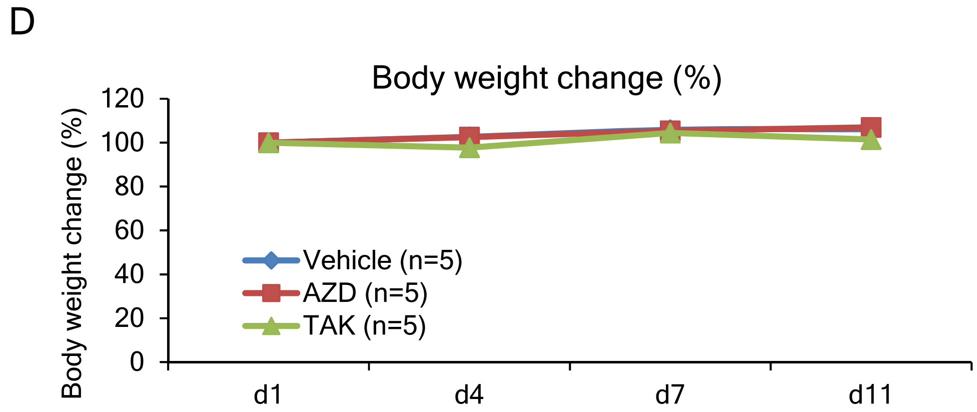

Figure S3. AZD0530 and TAK 165 suppressed BMP-7-induced HO. Related to Figure 5. BMP-7 was injected into the right gastrocnemius muscle of male C57BL/6 mice (6-8 weeks), who were administered drugs once daily 5 times a week intraperitoneally for 2 weeks thereafter. (A) X-rays (upper panels) and μCT (lower panels) observations. (B) Average heterotopic bone volume. (C) The number of mice harboring HO (> 20 mm^3 bone volume). (D) Body weight change of BMP-7-injected male C57BL/6 mice. Scale bars, 10 mm (A). Results are the mean ± standard error (SE). N = 5. *, $P < 0.05$; **, $P < 0.01$ by Dunnett's multiple comparisons *t*-test compared to the vehicle treatment group (B). No significant differences between the AZD- or TAK- administered group compared to the vehicle group in two-way repeated measures ANOVA followed by Dunnett's multiple comparisons *t*-test (D).

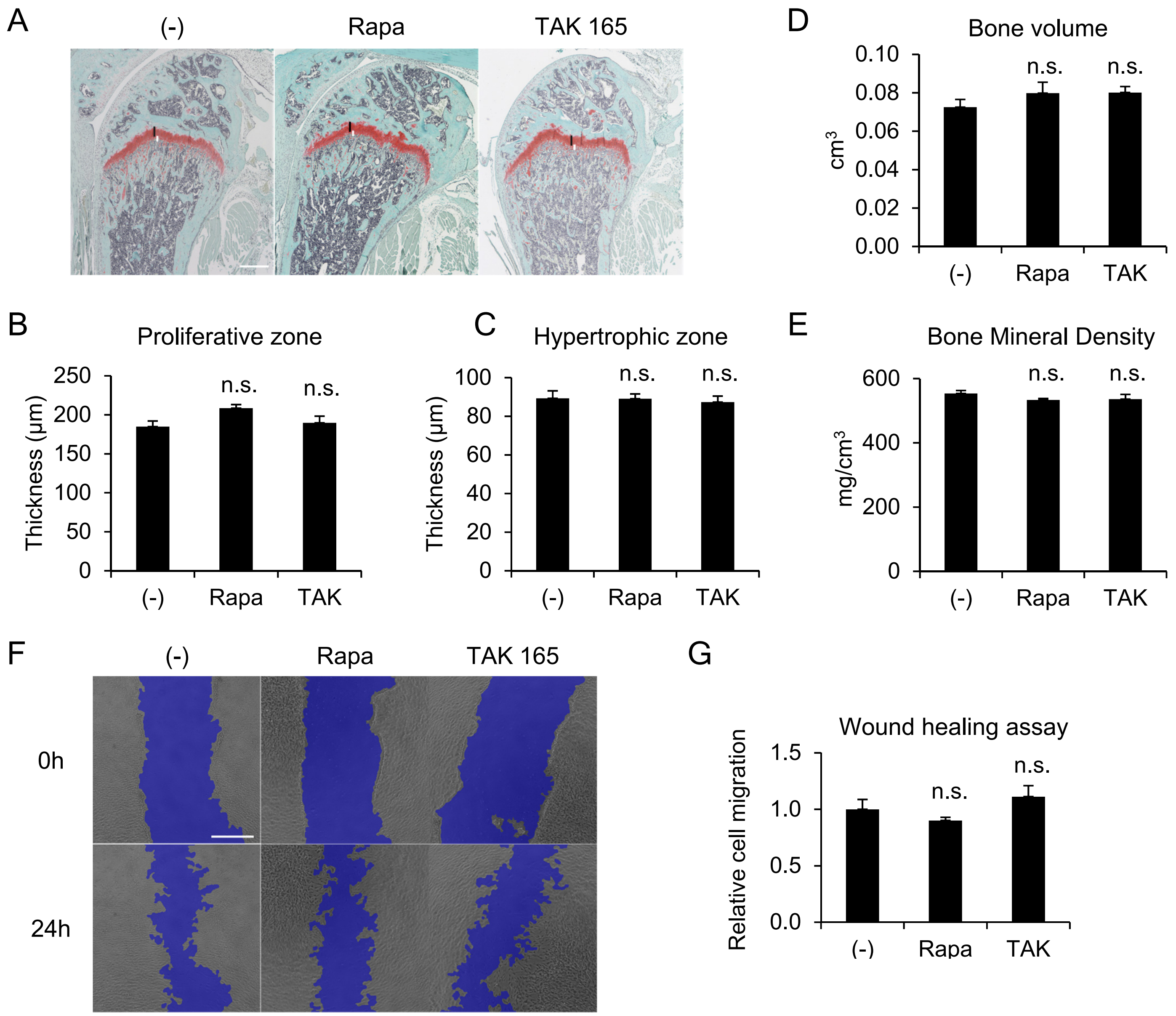

Figure S4. Tests of side effects of direct and indirect mTOR inhibitors. Related to Figure 5 and 6. (A-E) BMP-7 was injected into the right gastrocnemius muscle of male C57BL/6 mice (6-8 weeks), who were administered 5 mg/kg of compounds once daily 5 times a week intraperitoneally for 2 weeks thereafter. (A-C) Histological analysis of growth plate chondrocytes. Representative histological sections of the growth plate of tibiae from compounds-administered mice (A). Quantification of the thickness of the proliferative (B) and hypertrophic (C) zones is shown. Vertical lines indicate proliferative (black) and hypertrophic (white) zones. (C and D) Normal bone μCT of the femur-tibia of left legs from compounds-administered mice (F and G) Wound healing assay using iMSCs from healthy donor-derived iPSCs (414C2). iMSCs migrated to cover the scratched cell-free area. (E) Representative pictures and (F) relative migration values of iMSCs cultured in the medium with DMSO or test compounds (10 nM rapamycin or 1 μM TAK165). Scale bars, 0.5 mm (A and F). Results are the mean ± standard error (SE), n = 5 (Vehicle and TAK 165) or n = 4 (rapamycin) (A-E), or biological triplicate in 3 independent experiments (F and G). n.s., no significant difference by Dunnett's multiple comparisons *t*-test compared to the vehicle treatment group (B-E) or DMSO treatment control group (G).

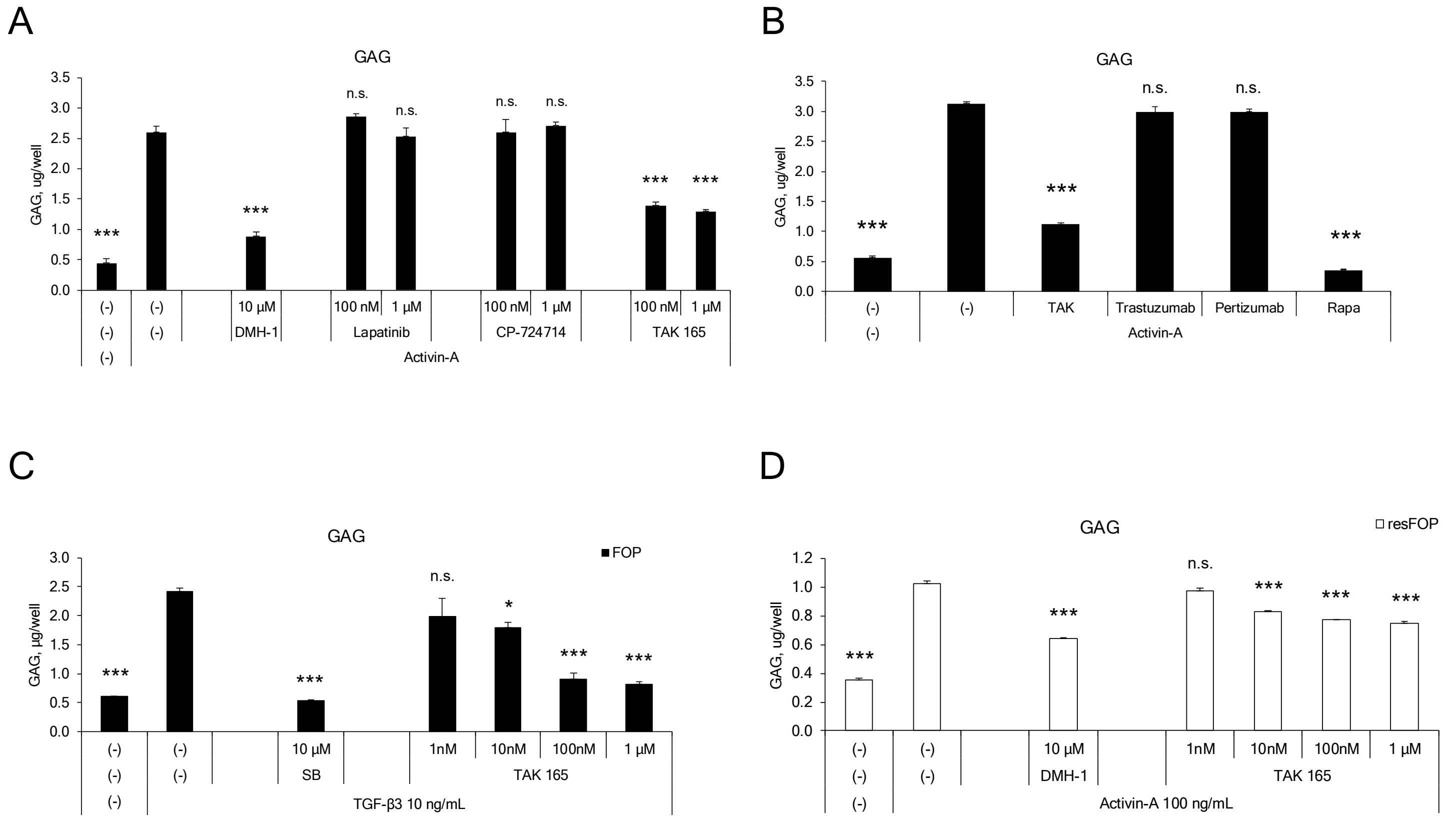

Figure S5. Analysis of the mechanism of TAK 165 action. Related to Figure 7. (A and B) ERBB2 inhibition did not suppress the chondrogenic induction of FOP-iMSCs. (A) Lapatinib (selective EGFR/ERBB2 inhibitor) and CP-724714 (selective ERBB2 inhibitor) or (B) 1 µg/mL Trastuzumab and 1 µg/mL Pertizumab (neutralizing antibodies against ERBB2) did not suppress the chondrogenesis of FOP-iMSCs. FOP-iMSCs were harvested 7 days after chondrogenic induction, which was performed with or without Activin-A, ERBB2 inhibitors or antibodies. TAK, 1 µM TAK 165; Rapa, 20 nM rapamycin. (C) TAK 165 suppressed the chondrogenesis of FOP-iMSCs stimulated with TGF-β3. (D) TAK 165 suppressed the chondrogenesis of resFOP-iMSCs stimulated with Activin-A. resFOP- or FOP-iMSCs were harvested 7 days after chondrogenic induction, which was performed with or without Activin-A or TGF-β3. Results are the mean ± standard error (SE) of biological triplicate using FOP-iPSCs (vFOP4-1) or its isogenic control iPSCs (resFOP-iPSCs). n.s., no significant difference; *, $P < 0.05$; ***, $P < 0.001$ by Dunnett's multiple comparisons *t*-test compared to the DMSO treatment control with Activin-A (A-C) or TGF-β3 (D).

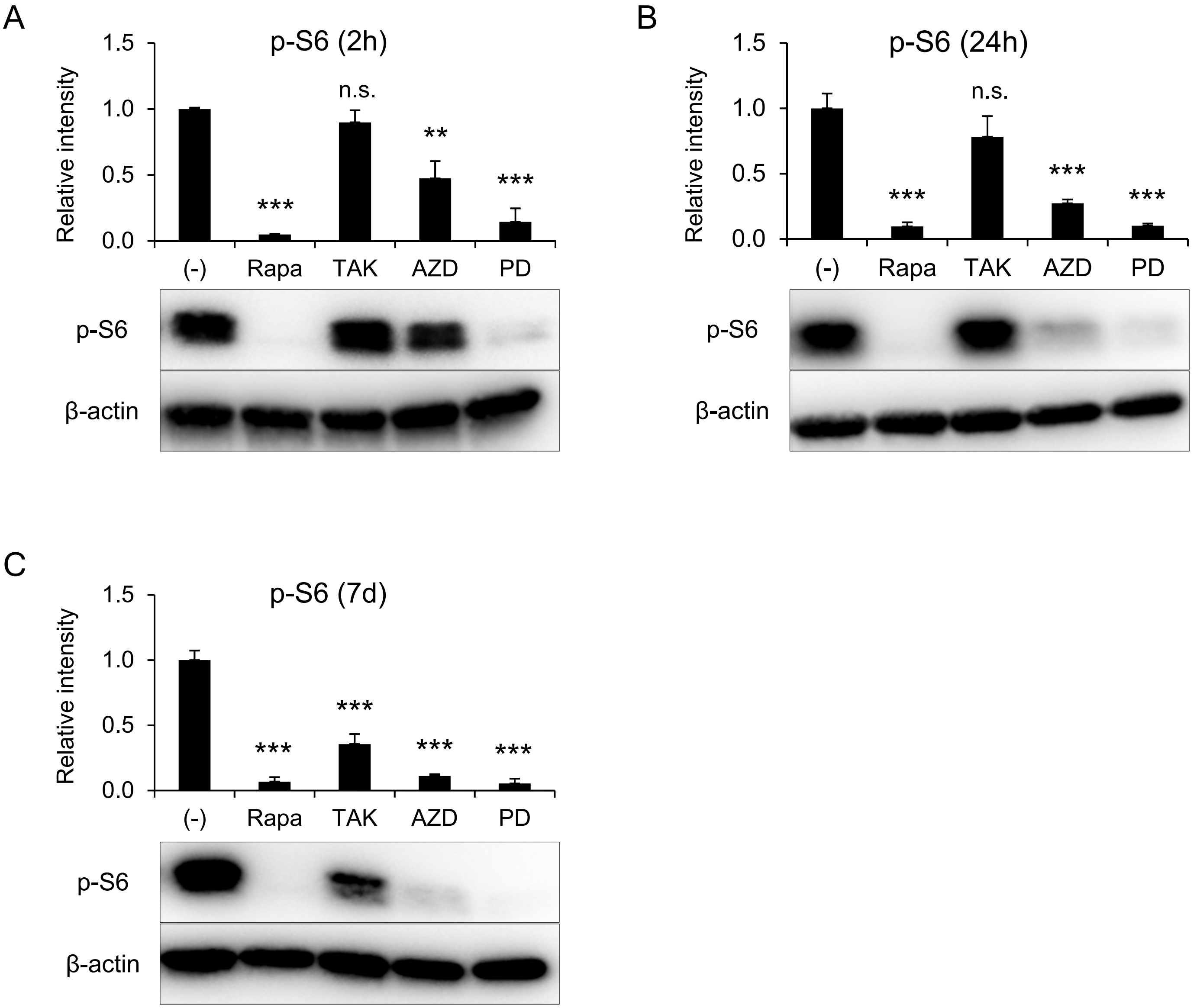

Figure S6. Distinct effects of AZD0530, PD 161570 and TAK 165 on mTOR signaling in chondrogenesis of FOP-iMSCs. Related to Figure 7. After 2 h (A), 24 h (B) or 7 days (C) of chondrogenic induction of FOP-iMSCs with Activin-A and test compounds, the cells were harvested, and p-S6 was assessed by western blotting. 10 nM rapamycin (Rapa) or 1 μM TAK 165 (TAK), AZD0530 (AZD) and PD 161570 (PD) were applied in the experiments. Results are the mean ± standard error (SE) of biological triplicate in 3 independent experiments using FOP-iPSCs (vFOP4-1). n.s., no significant difference; **, P < 0.01; ***, P < 0.001 by Dunnett's multiple comparisons *t*-test compared to the DMSO treatment control with Activin-A.

A

Experimental group	Activin-A	Compound
1	(+)	TAK 165
2	(+)	(-)
3	(+)	Lapatinib
4	(+)	CP-724714

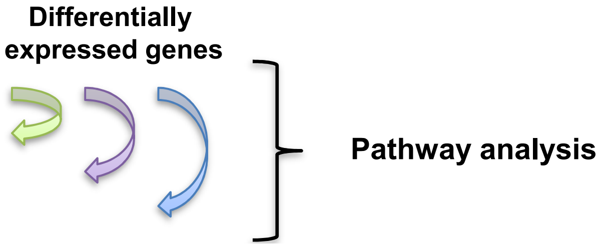

B

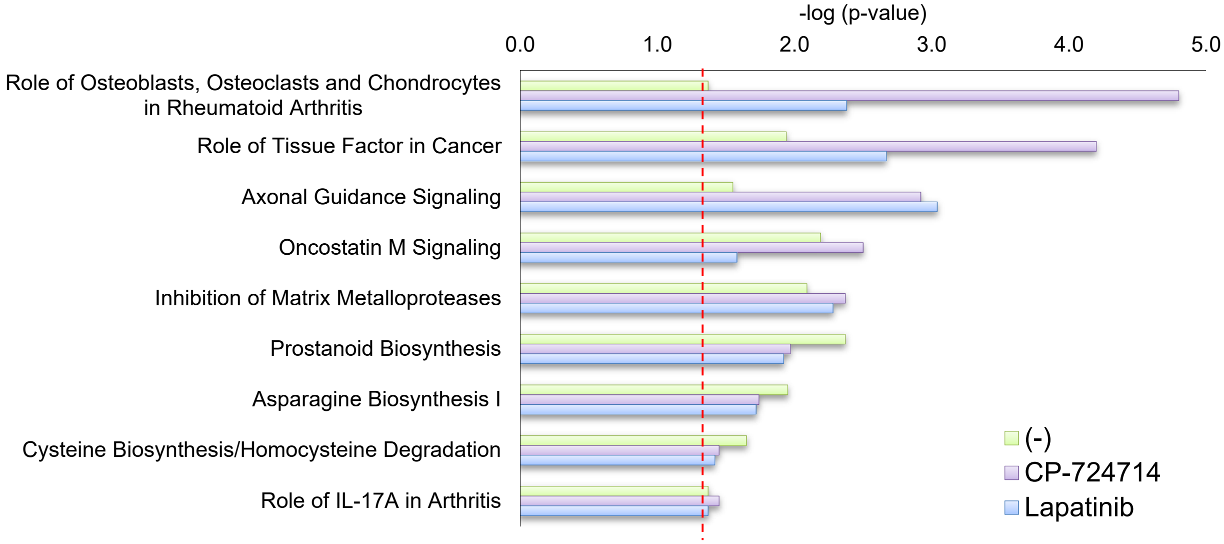

C

Symbol	Entrez Gene Name	(-)	Lapatinib	CP-724714
MMP1	matrix metallopeptidase 1	3.6	3.0	11.5
MMP13	matrix metallopeptidase 13	5.6	6.9	5.4
ITGA3	integrin subunit alpha 3	2.1	2.5	3.9
ITGB3	integrin subunit beta 3	2.8	3.6	3.3
FOS	Fos proto-oncogene, AP-1 transcription factor subunit	2.2	2.4	3.2
ADAMTS5	ADAM metallopeptidase with thrombospondin type 1 motif 5	0.5	2.1	3.1
BMP4	bone morphogenetic protein 4	0.5	0.4	5.2
DKK1	dickkopf WNT signaling pathway inhibitor 1	0.5	2.1	0.2
FRZB	frizzled-related protein	2.7	3.0	3.6
FZD3	frizzled class receptor 3	0.5	0.4	0.3
PIK3R3	phosphoinositide-3-kinase regulatory subunit 3	2.0	2.1	0.4
SPP1	secreted phosphoprotein 1	2.9	3.7	4.4
TNFRSF11B	TNF receptor superfamily member 11b	0.5	0.5	3.4
WIF1	WNT inhibitory factor 1	0.2	0.1	0.1
WNT16	Wnt family member 16	2.3	0.5	3.0

Figure S7. Pathway analysis of TAK 165 and other ERBB2 inhibitors in Activin-A-induced chondrogenesis of FOP-iMSCs. Related to Figure 7. Chondrogenic induction was performed in FOP-iMSCs with or without 100 ng/mL Activin-A, 100 nM TAK 165, 100 nM Lapatinib (selective EGFR/ERBB2 inhibitor) and 100 nM CP-724714 (selective ERBB2 inhibitor). After 7 days, cells were harvested, and microarray analysis was performed. (A) Experimental group and stimulation. (B and C) Differentially expressed genes (1.5 fold change) against the TAK 165-treated group were analyzed by Ingenuity Pathway Analysis. (B) Canonical Pathways significantly involved in all three groups. -log (p-value) > 1.3 (p = 0.05) was considered significant. (C) Expression ratio to the TAK 165-treated group of differentially expressed genes in "Role of Osteoblasts, Osteoclasts and Chondrocytes in Rheumatoid Arthritis" are shown. N = 1.
