# Supplementary material for: An mTOR Signaling Modulator Suppressed Heterotopic Ossification of Fibrodysplasia Ossificans Progressiva
Source: Stem Cell Reports. 2018 Nov 1;11(5):1106–19. doi: 10.1016/j.stemcr.2018.10.007 (PMC6235670; doi:10.1016/j.stemcr.2018.10.007)
Supplement: Document S2. Article plus Supplemental Information [file mmc2.pdf]

Stem Cell Reports
Article

ISSCR
OPEN ACCESS

An mTOR Signaling Modulator Suppressed Heterotopic Ossification of Fibrodysplasia Ossificans Progressiva

Kyosuke Hino,[1,2,10] Chengzhu Zhao,[3,10] Kazuhiko Horigome,[1,2] Megumi Nishio,[4] Yasue Okanishi,[5] Sanae Nagata,[2] Shingo Komura,[6,7] Yasuhiro Yamada,[7] Junya Toguchida,[2,4,8,9] Akira Ohta,[5] and Makoto Ikeya[3,*]

[1]iPS Cell-Based Drug Discovery, Sumitomo Dainippon Pharma Co., Ltd., Osaka 554-0022, Japan
[2]Department of Cell Growth and Differentiation, Center for iPS Cell Research and Application, Kyoto University, Kyoto 606-8507, Japan
[3]Department of Clinical Application, Center for iPS Cell Research and Application, Kyoto University, 53 Kawahara-cho, Shogoin, Sakyo-ku, Kyoto 606-8507, Japan
[4]Department of Regeneration Sciences and Engineering, Institute for Frontier Life and Medical Sciences, Kyoto University, Kyoto 606-8507, Japan
[5]Department of Fundamental Cell Technology, Center for iPS Cell Research and Application, Kyoto University, Kyoto 606-8507, Japan
[6]Department of Orthopaedic Surgery, Gifu University Graduate School of Medicine, Gifu 501-1194, Japan
[7]Department of Life Science Frontiers, Center for iPS Cell Research and Application, Kyoto University, Kyoto 606-8507, Japan
[8]Institute for Advancement of Clinical and Translational Science (iACT), Kyoto University Hospital, Kyoto 606-8507, Japan
[9]Department of Orthopaedic Surgery, Graduate School of Medicine, Kyoto University, Kyoto 606-8507, Japan
[10]Co-first author
*Correspondence: mikeya@cira.kyoto-u.ac.jp
https://doi.org/10.1016/j.stemcr.2018.10.007

SUMMARY

Fibrodysplasia ossificans progressiva (FOP) is a rare and intractable disorder characterized by extraskeletal bone formation through endochondral ossification. FOP patients harbor gain-of-function mutations in ACVR1 (FOP-ACVR1), a type I receptor for bone morphogenetic proteins. Despite numerous studies, no drugs have been approved for FOP. Here, we developed a high-throughput screening (HTS) system focused on the constitutive activation of FOP-ACVR1 by utilizing a chondrogenic ATDC5 cell line that stably expresses FOP-ACVR1. After HTS of 5,000 small-molecule compounds, we identified two hit compounds that are effective at suppressing the enhanced chondrogenesis of FOP patient-derived induced pluripotent stem cells (FOP-iPSCs) and suppressed the heterotopic ossification (HO) of multiple model mice, including FOP-ACVR1 transgenic mice and HO model mice utilizing FOP-iPSCs. Furthermore, we revealed that one of the hit compounds is an mTOR signaling modulator that indirectly inhibits mTOR signaling. Our results demonstrate that these hit compounds could contribute to future drug repositioning and the mechanistic analysis of mTOR signaling.

INTRODUCTION

Fibrodysplasia ossificans progressiva (FOP) is a rare genetic disease characterized by extraskeletal bone formation in soft tissue, including skeletal muscle, ligament, and tendon, where bone is not normally observed. Such ectopic bones are formed through endochondral ossification, a process whereby bone tissue replaces mature cartilage (Kaplan et al., 2005, 2007, 2008, 2012a; Shore et al., 2005; Shore and Kaplan, 2010; Zuscik et al., 2008). Approximately 90% of FOP patients share an R206H (617G>A) point mutation in the intracellular glycine- and serine-rich domain of ACVR1 (Shore et al., 2006), a type I receptor for bone morphogenetic proteins (BMPs) (Canalis et al., 2003; Gu et al., 1999; Hogan, 1996; Massague et al., 2000; Mishina et al., 1999; Miyazono et al., 2010; Mueller and Nickel, 2012; Piek et al., 1999; Urist, 1965; Wozney et al., 1988). This mutated ACVR1 (FOP-ACVR1) has been shown to confer ligand-independent constitutive activity and ligand-dependent hyperactivity in BMP signaling (Billings et al., 2008; Chaikuad et al., 2012; Fukuda et al., 2008). Moreover, by utilizing FOP patient-derived induced pluripotent stem cells (FOP-iPSCs) and FOP-ACVR1 conditional-on knockin mice, it has been shown that as its neofunction FOP-ACVR1 abnormally transduces BMP signaling in response to activin A, a molecule that normally transduces transforming growth factor β (TGF-β) signaling but not BMP signaling (Hatsell et al., 2015; Hino et al., 2015).

A number of studies have revealed drug candidates for FOP, including direct kinase inhibitors of the catalytic domain of BMP type I receptors, which consequently suppress the phosphorylation of the downstream effectors SMAD1/5/8 (Engers et al., 2013; Hamasaki et al., 2012; Hao et al., 2010; Mohedas et al., 2013; Sanvitale et al., 2013; Yu et al., 2008); RARγ agonists, which reduce the expression of SMAD1/5/8 by protein degradation (Chakkalakal et al., 2016; Pavey et al., 2016; Shimono et al., 2011; Sinha et al., 2016); an inhibitor of activin A signaling by an activin A-specific neutralizing antibody (Hatsell et al., 2015; Hino et al., 2015); mechanistic target of rapamycin (mTOR) inhibitors, which target enhanced chondrogenesis, hypoxic signaling, and inflammatory signaling (Agarwal et al., 2016; Hino et al., 2017); and others (Brennan

This is an open access article under the CC BY license (http://creativecommons.org/licenses/by/4.0/).

et al., 2017; Cappato et al., 2016; Convente et al., 2017; Kaplan et al., 2012b; Kitoh et al., 2013; Takahashi et al., 2012; Wang et al., 2016). Among these drug candidates, the RARγ agonist palovarotene, the anti-activin A antibody, and the mTOR inhibitor rapamycin are now under clinical trial. Although many attempts are ongoing, no drug is available for FOP, and a limited number of target molecules is reported.

For the identification of potential drug target molecules or pathways, phenotypic screenings that focus on the FOP pathology are an attractive approach but generally highly challenging to develop (Moffat et al., 2017). We previously reported phenotypic screening to modulate the enhanced chondrogenesis of FOP-iPSC-derived induced mesenchymal stromal cells (FOP-iMSCs) triggered by activin A (Hino et al., 2017). In that strategy, our concept was mainly based on the knowledge that trauma, surgery, inflammation, or viral infection often evoke episodic flare-ups that precede heterotopic ossification (HO) in FOP (Kaplan et al., 2005) and that one of the crucial initiators of HO is activin A activation (Hatsell et al., 2015; Hino et al., 2015). In contrast, another study reported a distinct feature of FOP pathology in that about half of FOP patients experienced the progression of HO without apparent flares, injury, or related events (Pignolo et al., 2016). Accordingly, we assumed that this pathology might be caused by ligand-independent constitutive activity such that FOP-ACVR1 transduces BMP signaling without ligand binding.

Featuring ligand-independent constitutive activity, here we established a phenotypic assay-based high-throughput screening (HTS) system focused on alkaline phosphatase (ALP), a well-established prehypertrophic chondrogenic marker (Zuscik et al., 2008), utilizing a chondrogenic ATDC5 cell line (Akiyama et al., 2000; Shukunami et al., 1997) that stably expresses FOP-ACVR1 (ATDC5/FOP-ACVR1). After HTS of approximately 5,000 small-molecule compounds, we identified three hit compounds: AZD0530 (also known as saracatinib), PD 161570, and TAK 165 (also known as mubritinib). These compounds suppressed the enhanced chondrogenesis in FOP-iMSCs, a critical step of HO in the FOP pathology. We subsequently showed their therapeutic effects on HO in three different *in vivo* models: a BMP-7-induced HO model, FOP model mice expressing FOP-ACVR1, and a FOP-iPSC-based HO model in which ectopic bones derived from FOP patient-derived cells are formed in mice. Mechanism-of-action studies indicated that AZD0530 and PD 161570 were inhibitors of both BMP and TGF-β signaling. On the other hand, TAK 165 was an mTOR signaling modulator that indirectly controlled mTOR signaling. These data extend the molecular basis of the HO induced in FOP patients.

RESULTS

Development of an HTS System Focused on Constitutive Activity of FOP-ACVR1

FOP-ACVR1 has been shown to render ligand-independent constitutive activity and ligand-dependent hyperactivity in BMP signaling (Billings et al., 2008; Chaikuad et al., 2012; Fukuda et al., 2008), and direct ACVR1 kinase inhibitors of the catalytic domain of BMP type I receptors are reported (Engers et al., 2013; Hamasaki et al., 2012; Hao et al., 2010; Mohedas et al., 2013; Sanvitale et al., 2013; Yu et al., 2008). Although these inhibitors are promising and effective on FOP model mice (Dey et al., 2016; Yu et al., 2008), new drug candidates that modulate FOP pathological conditions through undescribed mechanisms are also beneficial. Therefore, to screen direct BMP signaling inhibitors and FOP phenotype modulators at the same time, we focused on a chondrogenic cell line, ATDC5. ATDC5 cells are known to increase the expression of ALP by BMP stimulation in several days (Akiyama et al., 2000; Shukunami et al., 1997), and ALP activity can be detected by a chromogenic phosphatase substrate in an HTS format. Although ALP is also known to be a pluripotent marker, it is upregulated during chondrogenic induction consistently with other chondrogenic markers in ATDC5 cells (Shukunami et al., 1997), indicating that ALP is a chondrogenic marker at least in ATDC5 cells. We designed an ACVR1 expression vector utilizing the doxycycline (Dox)-inducible vector KW111 (Hayakawa et al., 2013; Woltjen et al., 2009) and generated ATDC5 cells stably expressing FOP-ACVR1 (R206H) or wild-type (WT)-ACVR1 (Figure 1A). After Dox treatment, ACVR1 expression was increased in a concentration-dependent manner (Figures 1B and S1). Expectedly, without BMP stimulation, ALP activity was increased in ATDC5 cells expressing FOP-ACVR1, but not in WT-ACVR1 (Figure 1C). This result indicates the constitutive activity of BMP signaling was triggered by FOP-ACVR1 expression. In addition to this constitutive activity, hyperactivity against BMP-4 and acquired responsiveness to activin A were observed in ATDC5-expressing FOP-ACVR1 (Figure 1D). These results indicated the validity of our assay system. DMH-1, a direct ACVR1 kinase inhibitor, suppressed the ALP activity of ATDC5 cells expressing FOP-ACVR1 without BMP stimulation in a concentration-dependent manner, also demonstrating that the constitutive activity of BMP signaling can be measured by ALP activity (Figure 1E). These results indicate that Dox-inducible ATDC5 cells enable us to screen inhibitors against the constitutive activity of FOP-ACVR1.

HTS and Follow-Up Screens Identified Seven Hit Compounds

Utilizing this HTS system, we performed a first screening ($n = 2$; test compounds = 1 μM, Figure 2A) against our

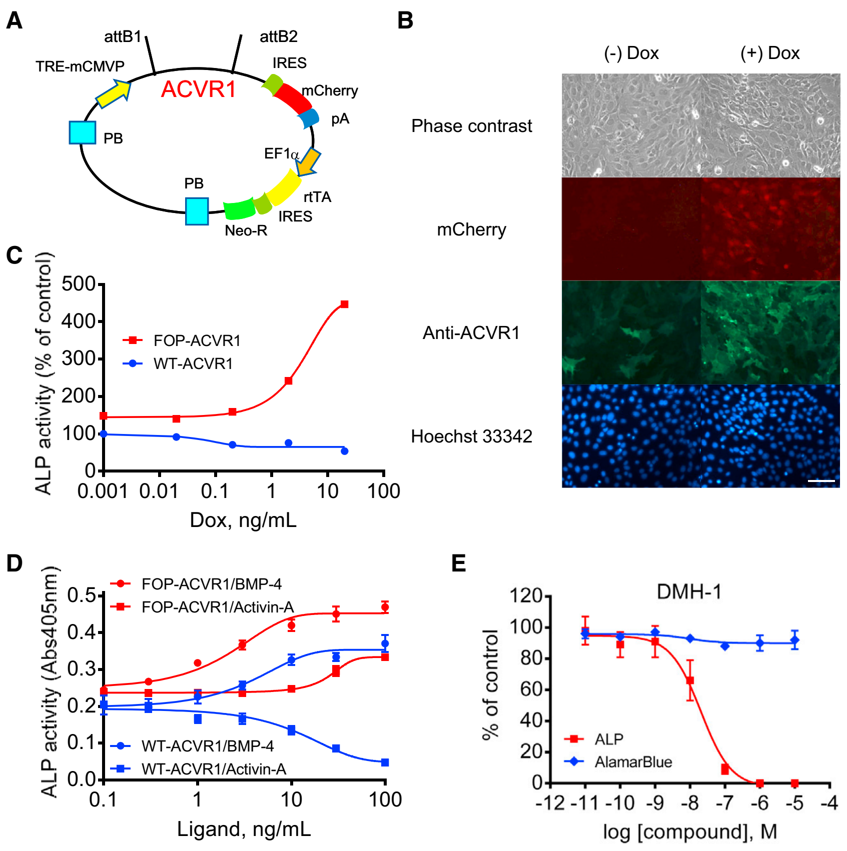

Figure 1. Construction and Validation of the Compound Screening System

(A) Vector map of the Dox-inducible ACVR1 expression vector.

(B) The expression of ACVR1 and mCherry in ATDC5/FOP-ACVR1 24 hr after 2 ng/mL Dox treatment. Scale bar, 100 μm.

(C) ALP activity of ATDC5/WT-ACVR1 or FOP-ACVR1 72 hr after Dox treatment.

(D) Concentration response curves of BMP-4 and activin A in ATDC5/WT-ACVR1 or FOP-ACVR1 72 hr after 3 ng/mL Dox and ligand treatment.

(E) DMH-1 (ACVR1 kinase inhibitor) inhibited the ALP activity but not the viability (AlamarBlue) of ATDC5/FOP-ACVR1. ALP and AlamarBlue assays were performed 72 hr after Dox and DMH-1 treatment.

Results are the mean ± SE, n = 1 (C) or biological triplicate in three independent experiments (D and E).

HTS library, which contains approximately 5,000 small-molecule compounds, most of which are marketed or bioactive (see also Supplemental Experimental Procedures). The scatterplot distribution of ALP activity and cell viability (Figures 2B and 2C), and Z′ factor and S/B ratio (Figures 2D and 2E) confirmed the validity of the HTS campaign. From the first screening, we obtained 160 hit compounds that fulfilled the criteria that more than 40% inhibition of ALP activity against DMSO control cells, less than 40% inhibition of viability and more than 20% of margin (inhibition of ALP activity [%] minus inhibition of viability [%]). A second screening was performed against the above 160 compounds (n = 2; test compounds = 0.1, 0.3, 1, 3 μM), and we identified 79 hit compounds that showed 40% inhibition of ALP activity against DMSO control cells and more than 50% of margin at any dose (Figures 2F and S2). A summary of HTS is shown in Figure 2G. Among them, RARγ agonists suppressed ALP activity, indicating the accuracy of our HTS system. To explore compounds that have potential to identify new mechanisms or contribute to future drug repositioning, we selected 14 compounds and performed a detailed concentration-dependent assay (Figure 3A). As a result, we identified seven compounds that showed stronger IC_{50} (<500 nM) and less toxicity (viability at 10 μM >50%) through our HTS campaign focused on the constitutive activity of FOP-ACVR1 (Figure 3B, red).

Further Validation of Seven Hit Compounds in FOP Patient-Derived iPSCs

To predict these seven compounds' therapeutic effects on FOP patients, we performed a FOP-iPSC-based chondrogenic assay. In this assay system, FOP-iMSCs (Fukuta et al., 2014; Hino et al., 2015, 2017; Matsumoto et al., 2015), a putative cell of origin of ectopic chondrogenesis, were treated with activin A, and the inhibitory effect of seven hit compounds was assessed at 1 μM (Figure 4A). Among them, AZD0530, PD 161570, and TAK 165 showed potent inhibition on glycosaminoglycan (GAG) production, which represents the amount of extracellular matrix secreted by chondrocytes. A detailed analysis against these three compounds revealed a concentration-dependent inhibitory effect on GAG in the chondrogenic assay of FOP-iMSCs (Figure 4B). Alcian blue staining, which stains acidic polysaccharides such as GAG in chondrocytes, also confirmed drug activity (Figure 4C). These results indicate that AZD0530, PD 161570, and TAK 165 have the potential to suppress the ectopic chondrogenesis of FOP patients.

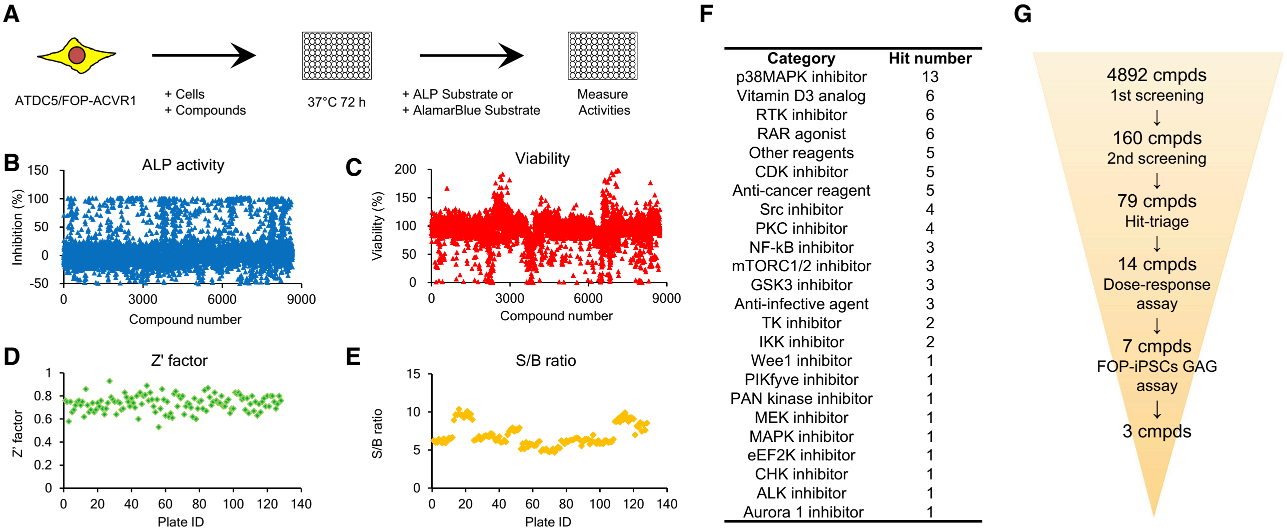

Figure 2. Schematic and Detailed Results of High-Throughput Screening

(A) Schematic of the first screening.

(B–E) Scatterplot distribution of ALP activity (B), viability (C), Z′ factor (D), and S/B ratio (E) from the first screening against 4,892 compounds.

(F) Classification of 79 hit compounds through the second screening.

(G) Results of the HTS campaign and follow-up screens.

Biological duplicates (B–F).

In Vivo Therapeutic Effects of AZD0530 and TAK 165

Next, the therapeutic effects of these drug candidates on FOP model mice were evaluated. We focused on AZD0530 and TAK 165 because they are applicable to *in vivo* experiments (Hennequin et al., 2006; Nagasawa et al., 2006). Previously, we generated FOP model mice that conditionally express hFOP-ACVR1 (R206H) by Dox administration and develop HO by muscle injury using cardiotoxin (CTX) (Hino et al., 2017). The intraperitoneal administration of AZD0530 or TAK 165 significantly suppressed the HO in these mice (Figures 5A–5C). In the CTX-injected site, we observed positive staining for safranin O (acidic proteoglycan, an extracellular matrix protein of chondrocytes), von Kossa (calcium deposition), and COL1 (bone marker) (Figure 5D, vehicle). On the other hand, mice administered AZD0530 or TAK 165 seemed to show less positive staining for von Kossa or COL1 (Figure 5D, AZD0530 and TAK 165). No apparent differences in body weight change was observed in mice administered AZD0530 or TAK 165 compared with vehicle (Figure 5E). These observations demonstrated that AZD0530 and TAK 165 are effective at suppressing HO in FOP model mice. These compounds' therapeutic effects were also confirmed in a BMP-7-induced HO model using WT mice (Figure S3). Furthermore, we validated whether AZD0530 and TAK 165 have the potential to suppress the HO of FOP patient-derived cells *in vivo*. We previously reported a human FOP-iPSC-based *in vivo* model (Hino et al., 2015, 2017). In this humanized FOP model, the transplantation of FOP-iMSCs and activin A-expressing cells into mice induces FOP patient-derived heterotopic bone *in vivo*. Notably, the administration of AZD0530 or TAK 165 significantly suppressed HO in these mice (Figures 6A–6C). Hypertrophic chondrocytes (based on safranin O and von Kossa staining) and von Kossa- and COL1-positive bone regions seemed to be fewer in mice administered AZD0530 or TAK 165 (Figure 6D). Because a large number of anti-human-specific vimentin-positive cells were observed in the AZD0530 and TAK 165-treated groups (Figure 6D), we could conclude that the therapeutic effect of these compounds was not due to the death of the human transplanted cells but rather the suppression of HO. In these experiments, neither AZD0530 nor TAK 165 administration decreased body weight (Figures 5E, 6E, and S3D), and the dosing used was comparable with that in previous studies (Hennequin et al., 2006; Nagasawa et al., 2006). TAK 165 in particular did not impair the chondrogenesis of normal chondrocytes (Figures S4A–S4C), normal skeletal development *in vivo* (Figures S4D and S4E), or wound healing *in vitro* (Figures S4F and S4G). Thus, we concluded the HO suppression was not primarily caused by toxicity, although further *in vivo* assessment might be preferable. Taken together, AZD0530 and TAK 165 are promising drug candidates since they suppressed the HO of FOP

A

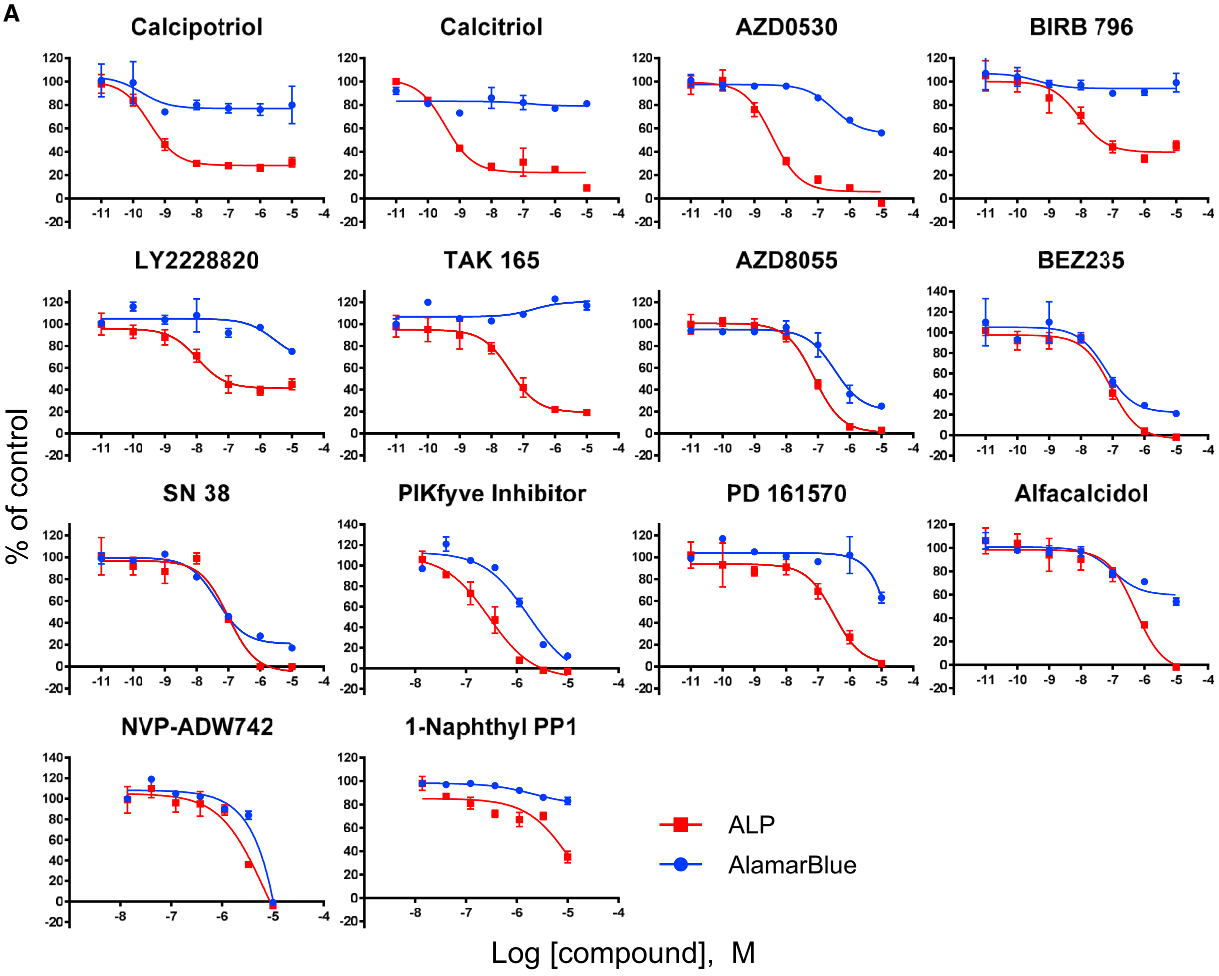

B

Name	IC50, nM	Viability (%) @10 μM	Highest stage	Putative mechanism
Calcipotriol	0.3	80	Launched	Vitamin D derivative
Calcitriol	0.4	81	Launched	Vitamin D derivative
AZD0530	4	56	Phase II	Src, Bcr-Abl inhibitor
BIRB 796	9	99	Phase III	p38 MAPK inhibitor
LY2228820	11	75	Phase II	p38 MAPK inhibitor
TAK 165	39	117	Phase I	ERBB2 inhibitor
AZD8055	77	25	Phase I	mTORC1/2 inhibitor
BEZ235	87	21	Phase II	PI3K, mTORC1/2 inhibitor
SN 38	98	17	Phase II	Topoisomerase-I inhibitor
PIKfyve Inhibitor	283	12	Preclinical	PIKfyve Inhibitor
PD 161570	317	63	Preclinical	FGFR inhibitor
Alfacalcidol	526	54	Launched	Vitamin D derivative
NVP-ADW742	6569	-1	Preclinical	IGF-1R inhibitor
1-Naphthyl PP1	12220	83	Preclinical	Src family kinase inhibitor
DMH-1	21	92	Preclinical	BMPR inhibitior

(legend on next page)

patient-derived cells *in vivo* in addition to the HO of FOP model mice.

Mechanisms of Action of AZD0530, PD 161570, and TAK 165

Finally, we analyzed the mechanisms of action of AZD0530, PD 161570, and TAK 165 on the chondrogenesis of FOP-iMSCs. Because it is known that BMP and TGF-β signaling are crucial in the chondrogenesis of FOP (Hino et al., 2015, 2017) and because our HTS system can detect BMP inhibitors, we assessed the direct effects of the three drugs on BMP and TGF-β signaling. AZD0530 and PD 161570 inhibited both BRE-Luc (BMP-specific luciferase reporter construct) and CAGA-Luc (TGF-β-responsive luciferase reporter construct) (Figures 7A and 7B). Therefore, we concluded AZD0530 and PD 161570 were BMP and TGF-β signaling dual inhibitors, and their mechanisms of action could contribute to the suppression of the chondrogenesis of FOP-iMSCs because they inhibited both pathways at similar drug concentration ranges during the chondrogenesis of FOP-iMSCs (Figure 4B). This result is in accordance with a previous study showing that AZD0530 inhibited BMP type I receptors (Lewis and Prywes, 2013). On the contrary, TAK 165 did not affect these signaling pathways. TAK 165 is an ERBB2 (also known as HER2)-selective kinase inhibitor (Anastassiadis et al., 2011; Nagasawa et al., 2006). To check the importance of ERBB2 inhibition in chondrogenesis, we performed a loss-of-function study using small interfering RNA (siRNA). Knockdown of *ERBB2* did not decrease GAG in the chondrogenesis of FOP-iMSCs (Figure 7C). Furthermore, another ERBB2-selective inhibitor (CP-724714), an ERBB1/2-selective inhibitor (lapatinib), or ERBB2-selective neutralizing antibodies (trastuzumab and pertuzumab) showed no effect on GAG in the chondrogenesis of FOP-iMSCs (Figures S5A and S5B). Given these results, the mechanism of action of TAK 165 was not through ERBB2 inhibition. We further investigated the effect of TAK 165 on TGF-β3-induced chondrogenesis in FOP-iMSCs and activin A-induced chondrogenesis in resFOP-iMSCs, in which the mutant ACVR1 was corrected to WT (Matsumoto et al., 2015) (Figures S5C and S5D). These results indicate that TAK 165 showed stronger effects on FOP cells than on normal cells. Recently, ourselves and Agarwal et al. have separately uncovered the impact of inhibiting mTOR signaling on the HO of FOP model mice and FOP-iMSCs (Agarwal et al., 2016; Hino et al., 2017). Therefore, we checked TAK 165's effect on mTOR signaling. First, to test whether TAK 165 is a direct inhibitor, we monitored the phosphorylation of S6 (p-S6), a well-known mTOR signaling surrogate marker, for 2 hr after treatment with TAK 165 in FOP-iMSCs cultured in 10% fetal bovine serum (FBS) (Figure 7D). In this condition, a strong p-S6 signal was detected. The mTOR inhibitor rapamycin decreased p-S6 levels, but TAK 165 did not. Next, we checked for indirect effects of TAK 165 on mTOR signaling in the chondrogenesis assay of FOP-iMSCs stimulated by activin A. After 24-hr stimulation with TAK 165 or CP-724714, no effects were observed on p-S6 (Figure 7E). Interestingly however, after 7 days of stimulation with TAK 165 but not CP-724714, p-S6 was dramatically decreased (Figure 7F). As expected, AZD0530 and PD 161570 significantly inhibited p-S6 levels from 2 hr after treatment (Figure S6), indicating that TAK 165 acts through a distinct mechanism. In addition, we performed an unbiased transcriptome analysis of FOP-iMSCs 7 days after inducing chondrogenesis by activin A (Figure S7). TAK 165, but not other ERBB2 inhibitors, affected genes that are involved in chondrogenesis or osteogenesis ("Role of Osteoblasts, Osteoclasts, and Chondrocytes in Rheumatoid Arthritis" in Figure S7B). These results indicate that TAK 165 indirectly modulated mTOR signaling and suppressed the chondrogenesis and HO of FOP.

DISCUSSION

In this report, we identified TAK 165 as a drug candidate for FOP. It is reported that TAK 165 is a selective inhibitor of ERBB2, a receptor tyrosine kinase often amplified or mutated in several cancers (Moasser, 2007). It is common that kinase inhibitors targeting catalytic domains show less selectivity, but interestingly TAK 165 is highly selective for ERBB2 against a panel of 300 recombinant protein kinases, presumably due to the fact that TAK 165 is an allosteric inhibitor of ERBB2 (Anastassiadis et al., 2011). Regardless of TAK 165's high selectivity to ERBB2, the suppression of chondrogenesis and HO by TAK 165 was not caused by ERBB2 inhibition but by indirect mTOR signaling inhibition (Figure 7). TAK 165 inhibited the ALP activity of constitutively activated FOP-ACVR1 in ATDC5 (Figure 3) and the enhanced chondrogenesis of FOP-iMSCs triggered by activin A (Figure 4). TAK 165 also modulated chondrogenesis-related pathways (Figure S7).

Figure 3. Detailed Dose-Response Assay Results of 14 Hit Compounds

(A) Dose-response curves of 14 hit compounds. ALP assay and AlamarBlue assay were performed using the same protocol as the HTS.

(B) IC_{50} values and viability (%) at 10 μM in the dose-response assay, highest stage, and putative mechanism of 14 hit compounds are shown. Seven compounds (red) satisfied the criteria (IC_{50} of ALP assay <500 nM and viability at 10 μM >50%).

Results are the mean ± SE, biological triplicates.

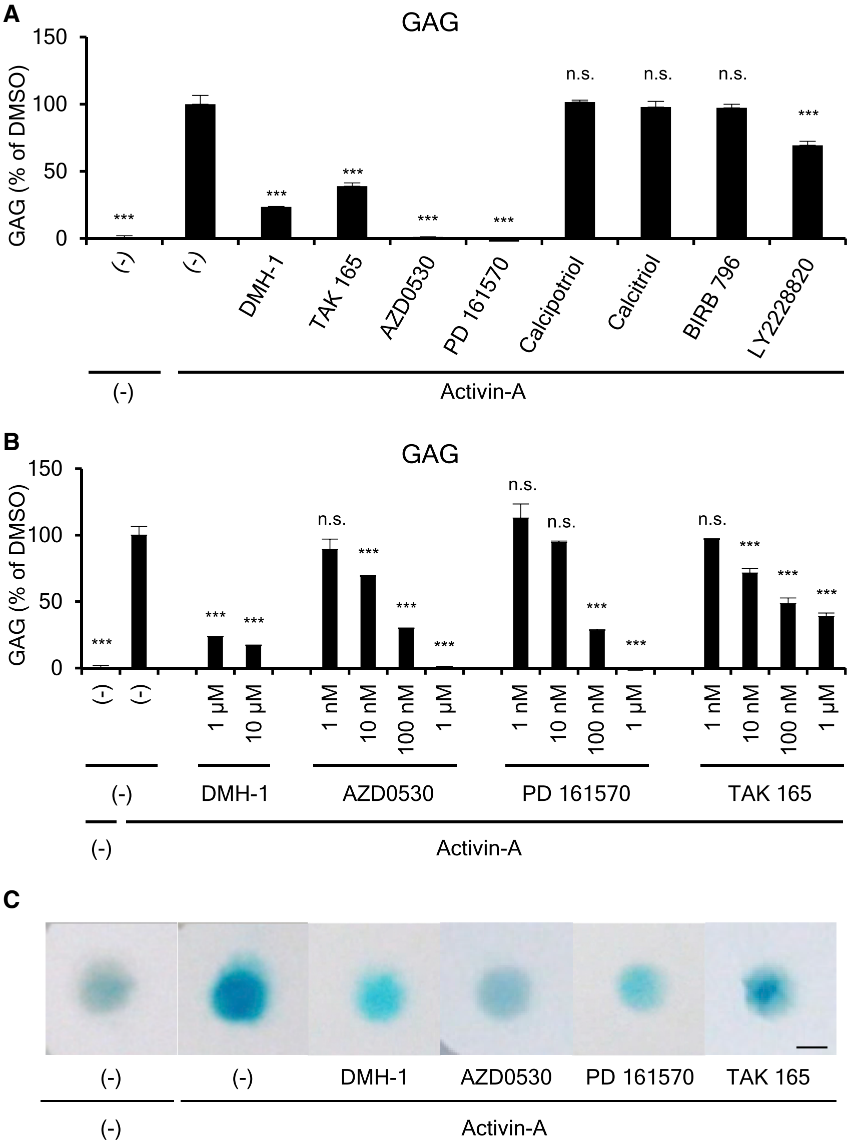

Figure 4. AZD0530, PD 161570, and TAK 165 Suppressed the Chondrogenic Induction of FOP-iMSCs

(A) The inhibitory effect of seven hit compounds on the chondrogenic induction of FOP-iMSCs. The cells were harvested 7 days after chondrogenic induction, which was performed with or without activin A and inhibitors (1 μM).

(B) AZD0530, PD 161570, and TAK 165 suppressed the chondrogenic induction of FOP-iMSCs in a dose-dependent manner.

(C) Alcian blue staining of DMH-1, AZD0530, PD 161570, and TAK 165.

Results are the mean ± SE, biological triplicates used FOP-iPSCs (vFOP4-1) (A and B). n.s., no significant difference; ***$p < 0.001$ by Dunnett's multiple comparisons t test compared with the DMSO treatment control with activin A. Scale bar, 200 μm.

These results suggested that TAK 165 affected chondrogenesis through indirect mTOR signaling modulation. Because TAK 165 showed obviously different effects on Alcian blue staining (Figure 4C), BMP and TGF-β signaling (Figures 7A and 7B), and mTOR signaling compared with other HTS hits (Figures 6D–6F and S6), TAK 165 might be useful for future concurrent treatment with other direct inhibitors. A detailed mechanism of action and the identification of direct targets of TAK 165 remain important issues awaiting future clarification.

To identify potential mechanisms that suppress the enhanced chondrogenesis of FOP, we developed an HTS system that focuses on the constitutive activity of FOP-ACVR1. Although we previously focused on activin A-triggered enhanced chondrogenesis, inspired by the recent report showing that a substantial number of FOP patients experience the progression of HO without apparent flares (Pignolo et al., 2016), we adopted the constitutive activity of FOP-ACVR1 for phenotypic screening system. We screened a library of about 5,000 small-molecule compounds and finally identified hit compounds that were effective in multiple HO model mice (Figures 5, 6, and S3). However, although effective, the effect of the hit compounds had high variability. Improving *in vivo* models will reduce variation caused by the incomplete purity of the mouse strain (Figure 5) or by the technical challenges of the transplantation assay (Figure 6). Another important issue is how to enhance the efficacy of our compounds *in vivo*. Since the hit compounds are prototypes or lead compounds, the solubility and pharmacokinetics might

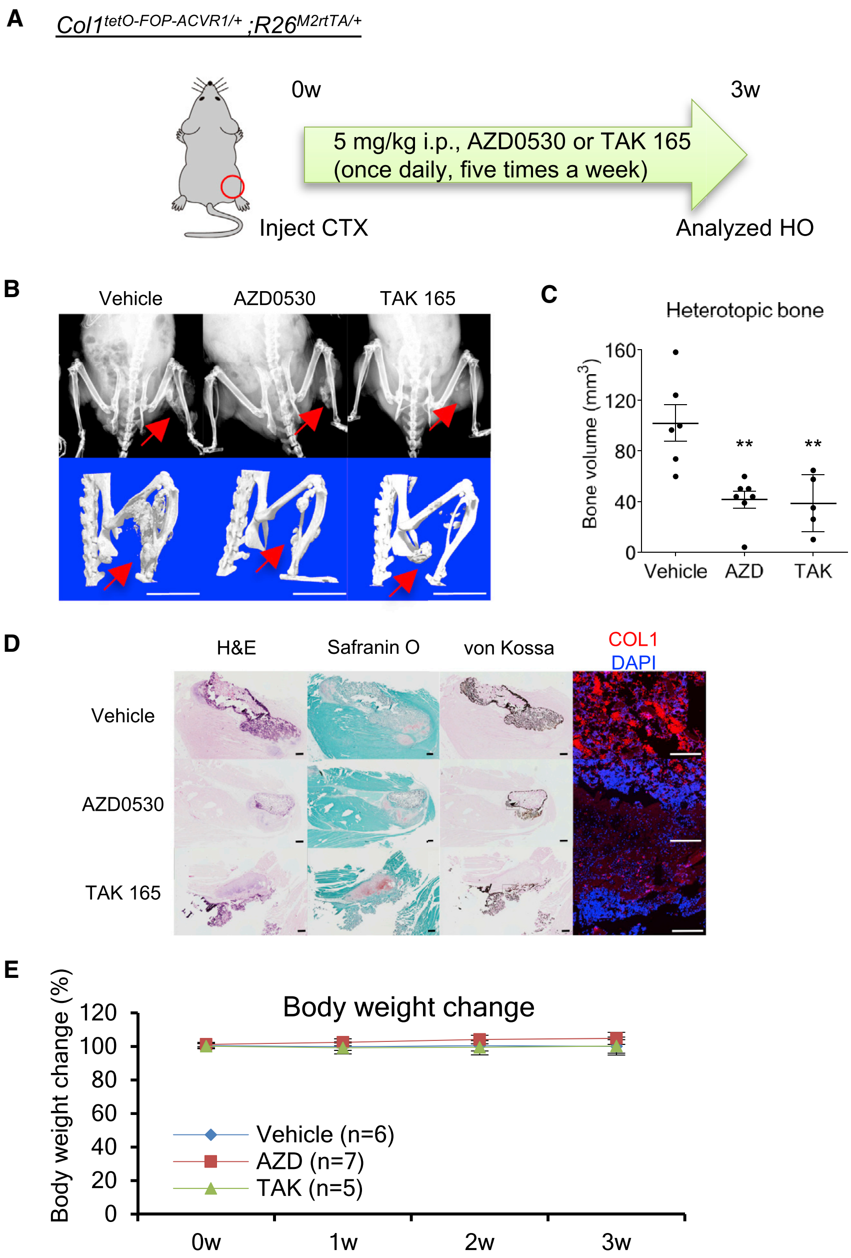

Figure 5. AZD0530 and TAK 165 Suppressed HO in FOP-ACVR1 Conditional Transgenic Mice

(A) Schematic of the *in vivo* efficacy study utilized FOP-ACVR1 conditional transgenic mice. Intraperitoneal (i.p.) administration of 5 mg/kg AZD0530 and TAK 165 (once daily, five times a week) suppressed the HO in FOP-ACVR1 (R206H) conditional transgenic mice. HO was induced by muscle injury triggered by cardiotoxin (CTX) injection and oral administration of Dox. Three weeks after CTX injection and drug administration, mice were analyzed.

(B) X-rays (upper panels) and μCT (lower panels) observations. Scale bars, 10 mm.

(C) Average heterotopic bone volume.

(D) Histological analysis of the CTX-injected region. H&E staining, safranin O staining (acidic proteoglycan), von Kossa staining (calcium), and anti-COL1 (bone) staining are shown. Scale bars, 100 μm (H&E, safranin O, and von Kossa) and 500 μm (COL1 and hVimentin).

(E) Body weight change (%) of mice administered compounds.

Results are the mean ± standard error (SE), n = 6 (vehicle), n = 7 (AZD0530), or n = 5 (TAK 165). $^{**}p < 0.01$ by Dunnett's multiple comparisons t test compared with vehicle treatment group (C). No significant differences between the AZD- or TAK-administered group compared with the vehicle group in two-way repeated-measures ANOVA followed by Dunnett's multiple comparisons t test (E).

be not well studied, hampering assessment of the maximal dose that suppresses HO.

There are two types of approaches in FOP drug discovery. The first approach is target-based and focuses on FOP-ACVR1 itself, e.g., kinase inhibition of FOP-ACVR1 or downregulation of *Acvr1* expression (Cappato et al., 2016; Engers et al., 2013; Hamasaki et al., 2012; Hao et al., 2010; Mohedas et al., 2013; Sanvitale et al., 2013; Yu et al., 2008). The second approach is phenotypic screening and focuses on HO-related phenotypes, for example, enhanced chondrogenesis, osteogenesis, and so forth (Shore and Kaplan, 2010). The former approach is quite logical and promising because it suppresses causal genes in FOP, but in general highly selective kinase inhibition is extremely challenging (Anastassiadis et al., 2011). On the other hand, phenotypic screening could highlight the most effective and/or novel mechanism that underlies FOP pathology, although the challenge here is to develop a robust system that screens compounds or gives further validation of candidates (Hino et al., 2017; Moffat et al., 2017). In this study, we performed HTS of inhibitors for ALP activity triggered by the constitutive activity of FOP-ACVR1 in the chondrogenic cell line ATDC5. Since ALP is a well-validated prehypertrophic chondrogenic marker,

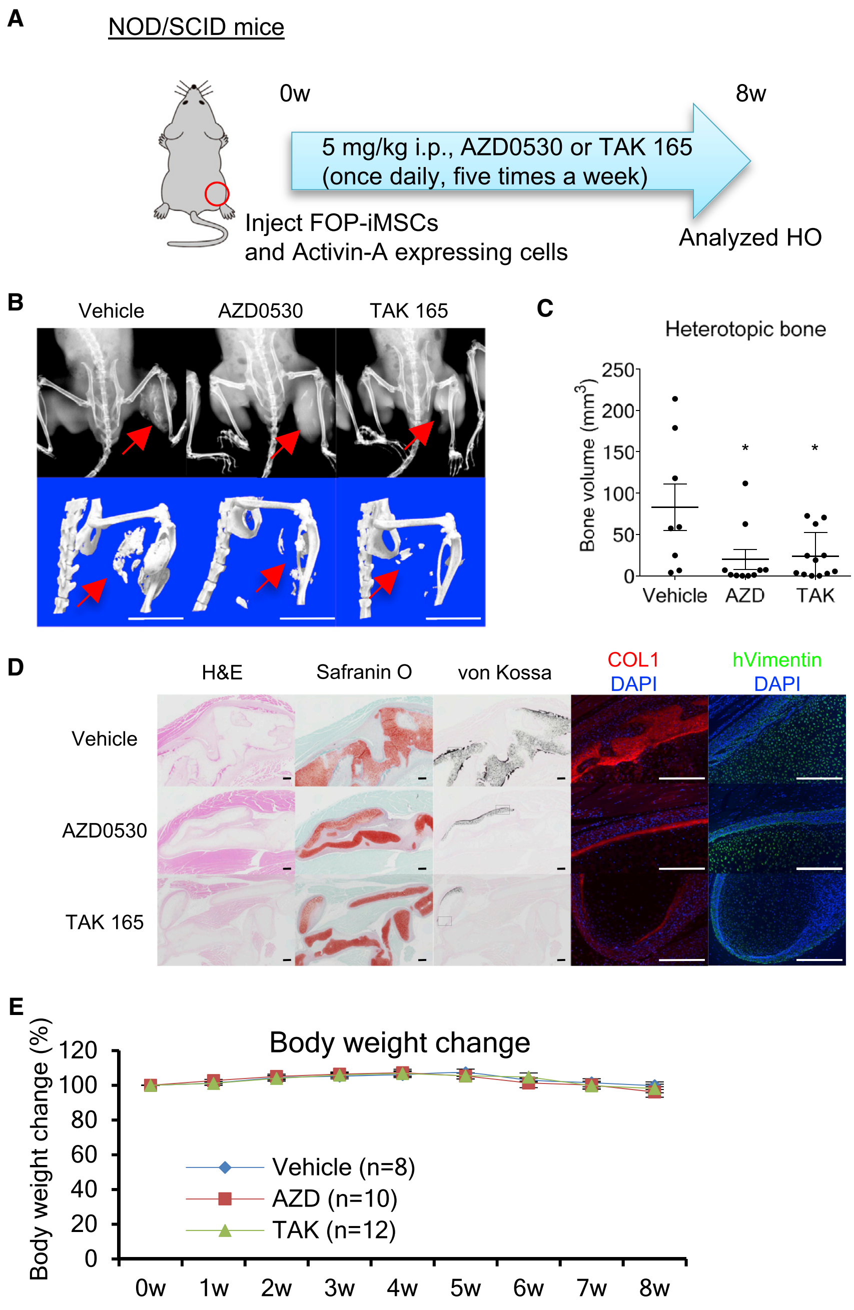

Figure 6. AZD0530 and TAK 165 Suppressed HO Derived from FOP-iMSCs *In Vivo*
(A) Schematic of the *in vivo* efficacy study utilized a human FOP-iPSC-based *in vivo* model. Intraperitoneal (i.p.) administration of 5 mg/kg AZD0530 or TAK 165 (once daily, five times a week) suppressed the HO derived from FOP-iMSCs triggered by activin A. Eight weeks after transplantation and drug administration, mice were analyzed.
(B) X-ray (upper panels) and μCT (lower panels) observations. Scale bars, 10 mm.
(C) Average heterotopic bone volume.
(D) Histological analysis of the cell-transplanted region. H&E, safranin O, von Kossa, anti-COL1, and anti-human vimentin staining are shown. Scale bars, 100 μm (H&E, safranin O, and von Kossa) and 500 μm (COL1 and hVimentin).
(E) Body weight change (%) of mice administered compounds.
Results are the mean ± SE, n = 8 (vehicle), n = 10 (AZD0530), or n = 12 (TAK165). *$p < 0.05$ by Dunnett's multiple comparisons t test compared with vehicle treatment group (C). No significant differences between the AZD- or TAK-administered group compared with the vehicle group in two-way repeated-measures ANOVA followed by Dunnett's multiple comparisons t test (E).

our HTS platform is a successful example of phenotypic screening for FOP. Consequently, we identified TAK 165, an mTOR signaling modulator that indirectly inhibits mTOR signaling, in addition to two direct ACVR1 kinase inhibitors (AZD0530 and PD 161570). Thus, phenotypic screening could contribute to understanding FOP pathophysiology.

In FOP patients, two phases, inflammation and the destruction of connective tissues (phase 1) and bone formation (phase 2), were proposed in the progression of HO (Shore and Kaplan, 2010), and each phenotype is a potential target for intervention. The suppression of phase 1 by anti-inflammatory drugs such as oral corticosteroids shows limited effects on FOP patients, but other approaches such as mast cell inhibitors might become new drug candidates (Brennan et al., 2017; Convente et al., 2017), although a future clinical trial is needed to prove the efficacy and side effects in FOP patients. Phase 2 can be further subdivided into three stages: fibroproliferation and angiogenesis (2A), chondrogenesis (2B), and osteogenesis (2C). We have

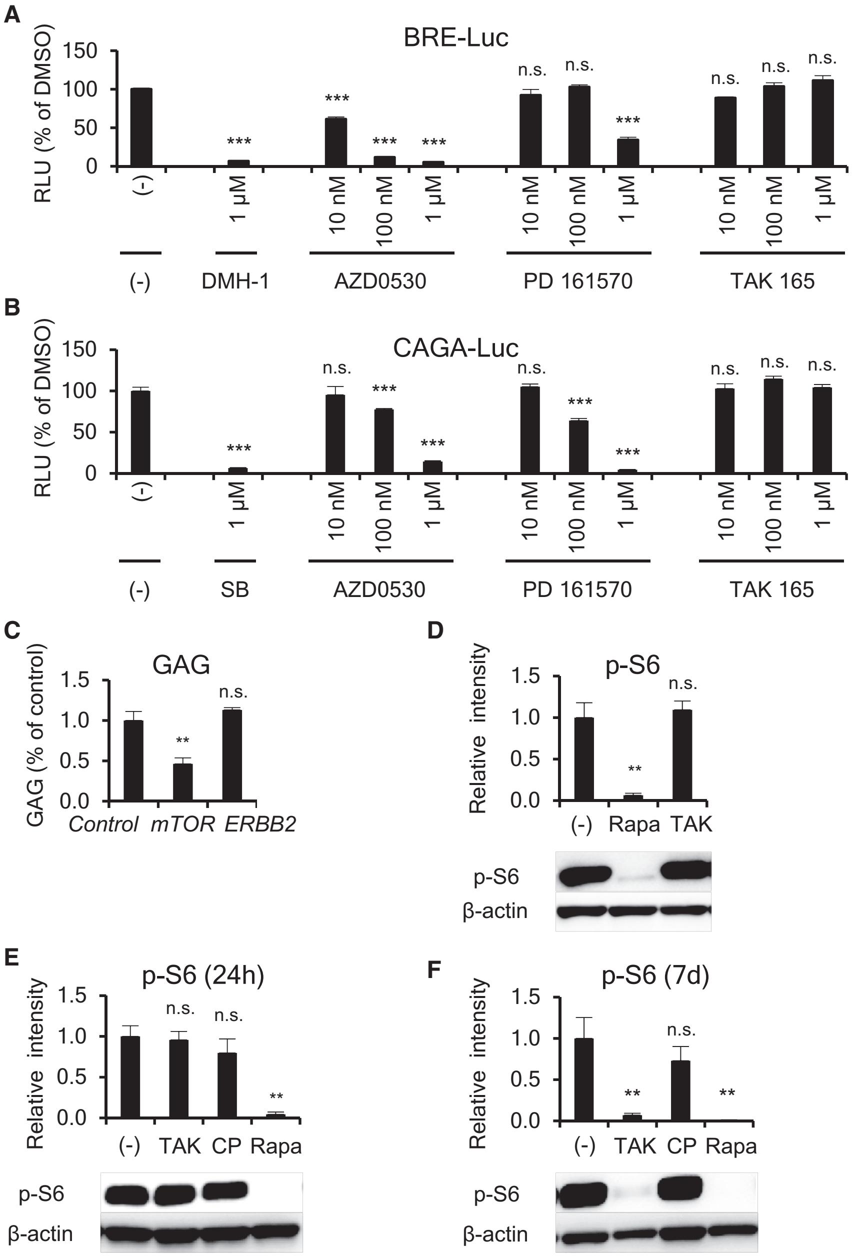

Figure 7. Mechanism of Action of AZD0530, PD 161570, and TAK 165

(A and B) AZD0530 and PD 161570, but not TAK 165, inhibited both BMP signaling (A) and TGF-β signaling (B). FOP-iMSCs transiently transfected with BRE-Luc (A) or CAGA-Luc (B) with CMV-*Renilla* were stimulated with activin A and compounds for 16 hr (A) or 3 hr (B).

(C) *ERBB2* knockdown did not reduce GAG content in the chondrogenic assay of FOP-iMSCs. One day after siRNA transfection, chondrogenic induction with activin A was initiated, and after 7 days the cells were harvested.

(D–F) TAK 165 indirectly inhibited mTOR signaling. (D) TAK 165 did not inhibit mTOR signaling directly, as assessed by western blotting of the phosphorylation of S6 (p-S6), a surrogate marker of mTORC1 activity. FOP-iMSCs cultured with 10% FBS were treated with 100 nM rapamycin (Rapa) or TAK 165 (TAK) for 2 hr, and the cells were harvested. (E and F) TAK 165 indirectly inhibited p-S6 during chondrogenic induction with activin A. After 24 hr or 7 days of chondrogenic induction of FOP-iMSCs with activin A and test compounds, the cells were harvested. 1 μM TAK, 1 μM CP (CP-724714, another selective ERBB2 inhibitor), or 10 nM Rapa were applied in the experiments.

Results are the mean ± SE of biological quadruplicates (A and B) or triplicates (C–F) using FOP-iPSCs (vFOP4-1). n.s., no significant difference; $^{**}p < 0.01$, $^{***}p < 0.001$ by Dunnett's multiple comparisons t test compared with the siRNA-transfected negative control and activin A (C) or with the DMSO treatment control and activin A (A, B, D–F).

focused on stage 2B, chondrogenesis, in both this and a previous study (Hino et al., 2017), because we assumed that the inhibition of chondrogenesis might not cause serious side effects since little or no chondrogenesis occurs in adults (Falah et al., 2010). On the other hand, as bone remodeling is a lifelong process (Maggioli and Stagi, 2017), the inhibition of stage 2C (osteogenesis) might evoke adverse effects, such as fracture or osteoporosis, regardless of any HO suppression. Although stage 2A (enhanced fibroproliferation) is often observed in the HO of FOP patients, no defined molecules have been reported for this process. Thus, a phenotypic screening focused on fibroproliferation could shed light on novel mechanisms of FOP. Three studies have identified the cell of origin of HO in FOP model mice (Agarwal et al., 2017; Dey et al., 2016; Lees-Shepard et al., 2018), and iMSCs or paraxial mesoderm-derived MSC-like cells can be induced from FOP-iPSCs (Hino et al., 2015, 2017; Matsumoto et al., 2015; Nakajima et al., 2018); therefore, these cells could be applicable to future phenotypic screenings for the inhibition of stage 2A. Combination therapy targeting multiple phases could be the best strategy for controlling the HO of FOP.

EXPERIMENTAL PROCEDURES

Full experimental procedures and associated references are available in Supplemental Experimental Procedures.

Study Approval

All experimental protocols dealing with human subjects were approved by the Ethics Committee of the Department of Medicine and Graduate School of Medicine, Kyoto University. Written informed consent was provided by each donor. All animal experiments were approved by the institutional animal committee of Kyoto University.

Chemicals Libraries

All chemical libraries were purchased from the suppliers listed in Supplemental Experimental Procedures. Almost all compounds were bioactive and/or annotated.

Cell Culture

ATDC5 cells were maintained in DMEM/F-12 (Thermo Fisher Scientific) supplemented with 5% (v/v) FBS (Nichirei). The FOP-iPSCs used in this study (previously described as vFOP4-1 [Matsumoto et al., 2013]) harbor the R206H heterozygous mutation in ACVR1, and gene-corrected resFOP-iPSCs were generated by BAC-based homologous recombination. These cells fulfilled several criteria for iPSCs including the expression of pluripotent markers, teratoma formation, normal karyotype, and morphology. Growth and gene expression profiles of the resFOP-iPSC clones were indistinguishable from the original FOP-iPSCs (Matsumoto et al., 2015); however, remarkably distinct responsiveness to activin A was observed (Hino et al., 2015).

ALP Assay

ATDC5/FOP-ACVR1 cells were plated in 96-well white plates (2,000 cells/well/40 μL, Corning) in DMEM/F-12 supplemented with 5% (v/v) FBS. Two hours after incubation at 37°C under 5% CO_2, 10 μL of test compounds (final 1 μM) was added, and the assay plates were incubated at 37°C under 5% CO_2. After 3 days of incubation, ALP activity was measured using an Amplite Colorimetric Alkaline Phosphatase Assay Kit (AAT Bioquest) according to the manufacturer's protocol.

2D Chondrogenic Induction

Chondrogenic induction was performed, and differentiation properties were assayed as previously described (Hino et al., 2015; Nasu et al., 2013; Umeda et al., 2012).

In Vivo Experiments

hFOP-ACVR1 conditional transgenic mice (Beard et al., 2006; Hino et al., 2017; Ohnishi et al., 2014; Yamada et al., 2013), BMP-7-induced HO model mice (Hino et al., 2017), and activin A-induced HO model mice transplanted with FOP-iMSCs (Hino et al., 2017) were intraperitoneally administered 5 mg/kg AZD0530, TAK 165, or rapamycin (once daily, five times a week) and analyzed as previously described (Hino et al., 2017).

Statistics

The statistical significance of all experiments was calculated by Prism 6 (GraphPad Software). p values less than 0.05 were considered statistically significant.

ACCESSION NUMBERS

Microarray data were deposited in the GEO of NCBI under the accession number GEO: GSE108771.

SUPPLEMENTAL INFORMATION

Supplemental Information includes Supplemental Experimental Procedures and seven figures and can be found with this article online at https://doi.org/10.1016/j.stemcr.2018.10.007.

AUTHOR CONTRIBUTIONS

Conceptualization and Project Administration, K. Hino and M.I.; Investigation, K. Hino, C.Z., K. Horigome, M.N., Y.O., S.N., S.K., and A.O.; Formal Analysis, K. Hino, C.Z., K. Horigome, M.N., and A.O.; Resources, K. Horigome and Y.Y.; Writing – Original Draft, K. Hino, J.T., and M.I. All authors read and approved the final manuscript.

DECLARATION OF INTERESTS

K. Hino and K. Horigome are employees of Sumitomo Dainippon Pharma Co. Ltd. M.I. and J.T. are supported by a research fund from Sumitomo Dainippon Pharma Co. Ltd. The other authors declare no competing interests.

ACKNOWLEDGMENTS

We thank Dr. K. Woltjen for providing the KW111 vector, Dr. A. Hotta for providing the PBaseII vector, S. Kihara for analysis of the wound healing assay, the Center for Anatomical, Pathological and Forensic Medical Researches, Kyoto University Graduate School of Medicine, for preparing the microscope slides, Dr. A. Ikeda for invaluable comments and discussion, Dr. P. Karagiannis for reading the manuscript, Dr. S. Yamanaka for supporting/initiating our FOP research, and members of the J.T. and M.I. laboratories for their support during this study. This work was supported by grants-in-aid for scientific research from the Japan Society for the Promotion of Science (JSPS) (#25293320, #16K15662), the Program for Intractable Diseases Research utilizing Disease-Specific iPS cells from the Japan Science and Technology Agency (JST) and the Japan Agency for Medical Research and Development (AMED), the Core Center for iPS Cell Research of the Research Center Network for Realization of Regenerative Medicine (JST/AMED), a grant from Research on Development of New Drugs (AMED), and a grant from the iPS Cell Research Fund, awarded in part to M.I. and J.T. M.I. was also supported by the Practical Research Project for Rare/Intractable Diseases and the Acceleration Program for Intractable Diseases Research utilizing Disease-Specific iPS cells from AMED. C.Z. was also supported by Grant-in-Aid for Young Scientists B from JSPS (17K15617) and the Future Development Funding Program of Kyoto University Research Coordination Alliance. A.O. was also supported by Research Project for Practical Applications of Regenerative Medicine from AMED. These funders

had no role in the study design, data collection and analysis, decision to publish, or preparation of the manuscript. We would like to thank Mr. M. Todani for drawing an illustration of graphical abstract.

Received: April 9, 2018
Revised: October 5, 2018
Accepted: October 5, 2018
Published: November 1, 2018

REFERENCES

Agarwal, S., Loder, S., Brownley, C., Cholok, D., Mangiavini, L., Li, J., Breuler, C., Sung, H.H., Li, S., Ranganathan, K., et al. (2016). Inhibition of Hif1alpha prevents both trauma-induced and genetic heterotopic ossification. Proc. Natl. Acad. Sci. U S A *113*, E338–E347.

Agarwal, S., Loder, S.J., Cholok, D., Peterson, J., Li, J., Breuler, C., Cameron Brownley, R., Hsin Sung, H., Chung, M.T., Kamiya, N., et al. (2017). Scleraxis-lineage cells contribute to ectopic bone formation in muscle and tendon. Stem Cells *35*, 705–710.

Akiyama, H., Shukunami, C., Nakamura, T., and Hiraki, Y. (2000). Differential expressions of BMP family genes during chondrogenic differentiation of mouse ATDC5 cells. Cell Struct. Funct. *25*, 195–204.

Anastassiadis, T., Deacon, S.W., Devarajan, K., Ma, H., and Peterson, J.R. (2011). Comprehensive assay of kinase catalytic activity reveals features of kinase inhibitor selectivity. Nat. Biotechnol. *29*, 1039–1045.

Beard, C., Hochedlinger, K., Plath, K., Wutz, A., and Jaenisch, R. (2006). Efficient method to generate single-copy transgenic mice by site-specific integration in embryonic stem cells. Genesis *44*, 23–28.

Billings, P.C., Fiori, J.L., Bentwood, J.L., O'Connell, M.P., Jiao, X., Nussbaum, B., Caron, R.J., Shore, E.M., and Kaplan, F.S. (2008). Dysregulated BMP signaling and enhanced osteogenic differentiation of connective tissue progenitor cells from patients with fibrodysplasia ossificans progressiva (FOP). J. Bone Miner. Res. *23*, 305–313.

Brennan, T.A., Lindborg, C.M., Bergbauer, C.R., Wang, H., Kaplan, F.S., and Pignolo, R.J. (2017). Mast cell inhibition as a therapeutic approach in fibrodysplasia ossificans progressiva (FOP). Bone *109*, 259–266.

Canalis, E., Economides, A.N., and Gazzerro, E. (2003). Bone morphogenetic proteins, their antagonists, and the skeleton. Endocr. Rev. *24*, 218–235.

Cappato, S., Tonachini, L., Giacopelli, F., Tirone, M., Galietta, L.J., Sormani, M., Giovenzana, A., Spinelli, A.E., Canciani, B., Brunelli, S., et al. (2016). High-throughput screening for modulators of ACVR1 transcription: discovery of potential therapeutics for fibrodysplasia ossificans progressiva. Dis. Model. Mech. *9*, 685–696.

Chaikuad, A., Alfano, I., Kerr, G., Sanvitale, C.E., Boergermann, J.H., Triffitt, J.T., von Delft, F., Knapp, S., Knaus, P., and Bullock, A.N. (2012). Structure of the bone morphogenetic protein receptor ALK2 and implications for fibrodysplasia ossificans progressiva. J. Biol. Chem. *287*, 36990–36998.

Chakkalakal, S.A., Uchibe, K., Convente, M.R., Zhang, D., Economides, A.N., Kaplan, F.S., Pacifici, M., Iwamoto, M., and Shore, E.M. (2016). Palovarotene inhibits heterotopic ossification and maintains limb mobility and growth in mice with the human acvr1(r206h) fibrodysplasia ossificans progressiva (fop) mutation. J. Bone Miner. Res. *31*, 1666–1675.

Convente, M.R., Chakkalakal, S.A., Yang, E., Caron, R.J., Zhang, D., Kambayashi, T., Kaplan, F.S., and Shore, E.M. (2017). Depletion of mast cells and macrophages impairs heterotopic ossification in an $acvr1^{R206H}$ mouse model of fibrodysplasia ossificans progressiva. J. Bone Miner. Res. *33*, 269–282.

Dey, D., Bagarova, J., Hatsell, S.J., Armstrong, K.A., Huang, L., Ermann, J., Vonner, A.J., Shen, Y., Mohedas, A.H., Lee, A., et al. (2016). Two tissue-resident progenitor lineages drive distinct phenotypes of heterotopic ossification. Sci. Transl. Med. *8*, 366ra163.

Engers, D.W., Frist, A.Y., Lindsley, C.W., Hong, C.C., and Hopkins, C.R. (2013). Synthesis and structure-activity relationships of a novel and selective bone morphogenetic protein receptor (BMP) inhibitor derived from the pyrazolo[1.5-a]pyrimidine scaffold of dorsomorphin: the discovery of ML347 as an ALK2 versus ALK3 selective MLPCN probe. Bioorg. Med. Chem. Lett. *23*, 3248–3252.

Falah, M., Nierenberg, G., Soudry, M., Hayden, M., and Volpin, G. (2010). Treatment of articular cartilage lesions of the knee. Int. Orthop. *34*, 621–630.

Fukuda, T., Kanomata, K., Nojima, J., Kokabu, S., Akita, M., Ikebuchi, K., Jimi, E., Komori, T., Maruki, Y., Matsuoka, M., et al. (2008). A unique mutation of ALK2, G356D, found in a patient with fibrodysplasia ossificans progressiva is a moderately activated BMP type I receptor. Biochem. Biophys. Res. Commun. *377*, 905–909.

Fukuta, M., Nakai, Y., Kirino, K., Nakagawa, M., Sekiguchi, K., Nagata, S., Matsumoto, Y., Yamamoto, T., Umeda, K., Heike, T., et al. (2014). Derivation of mesenchymal stromal cells from pluripotent stem cells through a neural crest lineage using small molecule compounds with defined media. PLoS One *9*, e112291.

Gu, Z., Reynolds, E.M., Song, J., Lei, H., Feijen, A., Yu, L., He, W., MacLaughlin, D.T., van den Eijnden-van Raaij, J., Donahoe, P.K., et al. (1999). The type I serine/threonine kinase receptor ActRIA (ALK2) is required for gastrulation of the mouse embryo. Development *126*, 2551–2561.

Hamasaki, M., Hashizume, Y., Yamada, Y., Katayama, T., Hohjoh, H., Fusaki, N., Nakashima, Y., Furuya, H., Haga, N., Takami, Y., et al. (2012). Pathogenic mutation of ALK2 inhibits induced pluripotent stem cell reprogramming and maintenance: mechanisms of reprogramming and strategy for drug identification. Stem Cells *30*, 2437–2449.

Hao, J., Ho, J.N., Lewis, J.A., Karim, K.A., Daniels, R.N., Gentry, P.R., Hopkins, C.R., Lindsley, C.W., and Hong, C.C. (2010). In vivo structure-activity relationship study of dorsomorphin analogues identifies selective VEGF and BMP inhibitors. ACS Chem. Biol. *5*, 245–253.

Hatsell, S.J., Idone, V., Wolken, D.M., Huang, L., Kim, H.J., Wang, L., Wen, X., Nannuru, K.C., Jimenez, J., Xie, L., et al. (2015). ACVR1R206H receptor mutation causes fibrodysplasia ossificans progressiva by imparting responsiveness to activin A. Sci. Transl. Med. *7*, 303ra137.

Hayakawa, K., Ikeya, M., Fukuta, M., Woltjen, K., Tamaki, S., Takahara, N., Kato, T., Jr., Sato, S., Otsuka, T., and Toguchida, J. (2013). Identification of target genes of synovial sarcoma-associated fusion oncoprotein using human pluripotent stem cells. Biochem. Biophys. Res. Commun. *432*, 713–719.

Hennequin, L.F., Allen, J., Breed, J., Curwen, J., Fennell, M., Green, T.P., Lambert-van der Brempt, C., Morgentin, R., Norman, R.A., Olivier, A., et al. (2006). N-(5-chloro-1,3-benzodioxol-4-yl)-7-[2-(4-methylpiperazin-1-yl)ethoxy]-5- (tetrahydro-2H-pyran-4-yloxy)quinazolin-4-amine, a novel, highly selective, orally available, dual-specific c-Src/Abl kinase inhibitor. J. Med. Chem. *49*, 6465–6488.

Hino, K., Horigome, K., Nishio, M., Komura, S., Nagata, S., Zhao, C., Jin, Y., Kawakami, K., Yamada, Y., Ohta, A., et al. (2017). Activin-A enhances mTOR signaling to promote aberrant chondrogenesis in fibrodysplasia ossificans progressiva. J. Clin. Invest. *127*, 3339–3352.

Hino, K., Ikeya, M., Horigome, K., Matsumoto, Y., Ebise, H., Nishio, M., Sekiguchi, K., Shibata, M., Nagata, S., Matsuda, S., et al. (2015). Neofunction of ACVR1 in fibrodysplasia ossificans progressiva. Proc. Natl. Acad. Sci. U S A *112*, 15438–15443.

Hogan, B.L. (1996). Bone morphogenetic proteins: multifunctional regulators of vertebrate development. Genes Dev. *10*, 1580–1594.

Kaplan, F., Glaser, D., Shore, E., Deirmengian, G., Gupta, R., Delai, P., Morhart, R., Smith, R., Le Merrer, M., Rogers, J., et al. (2005). The phenotype of fibrodysplasia ossificans progressiva. Clin. Rev. Bone Miner. Metab. *3*, 183–188.

Kaplan, F.S., Groppe, J., Pignolo, R.J., and Shore, E.M. (2007). Morphogen receptor genes and metamorphogenes: skeleton keys to metamorphosis. Ann. N. Y. Acad. Sci. *1116*, 113–133.

Kaplan, F.S., Le Merrer, M., Glaser, D.L., Pignolo, R.J., Goldsby, R.E., Kitterman, J.A., Groppe, J., and Shore, E.M. (2008). Fibrodysplasia ossificans progressiva. Best Pract. Res. Clin. Rheumatol. *22*, 191–205.

Kaplan, F.S., Chakkalakal, S.A., and Shore, E.M. (2012a). Fibrodysplasia ossificans progressiva: mechanisms and models of skeletal metamorphosis. Dis. Model. Mech. *5*, 756–762.

Kaplan, J., Kaplan, F.S., and Shore, E.M. (2012b). Restoration of normal BMP signaling levels and osteogenic differentiation in FOP mesenchymal progenitor cells by mutant allele-specific targeting. Gene Ther. *19*, 786–790.

Kitoh, H., Achiwa, M., Kaneko, H., Mishima, K., Matsushita, M., Kadono, I., Horowitz, J.D., Sallustio, B.C., Ohno, K., and Ishiguro, N. (2013). Perhexiline maleate in the treatment of fibrodysplasia ossificans progressiva: an open-labeled clinical trial. Orphanet J. Rare Dis. *8*, 163.

Lees-Shepard, J.B., Yamamoto, M., Biswas, A.A., Stoessel, S.J., Nicholas, S.E., Cogswell, C.A., Devarakonda, P.M., Schneider, M.J., Jr., Cummins, S.M., Legendre, N.P., et al. (2018). Activin-dependent signaling in fibro/adipogenic progenitors causes fibrodysplasia ossificans progressiva. Nat. Commun. *9*, 471.

Lewis, T.C., and Prywes, R. (2013). Serum regulation of Id1 expression by a BMP pathway and BMP responsive element. Biochim. Biophys. Acta *1829*, 1147–1159.

Maggioli, C., and Stagi, S. (2017). Bone modeling, remodeling, and skeletal health in children and adolescents: mineral accrual, assessment and treatment. Ann. Pediatr. Endocrinol. Metab. *22*, 1–5.

Massague, J., Blain, S.W., and Lo, R.S. (2000). TGFbeta signaling in growth control, cancer, and heritable disorders. Cell *103*, 295–309.

Matsumoto, Y., Hayashi, Y., Schlieve, C.R., Ikeya, M., Kim, H., Nguyen, T.D., Sami, S., Baba, S., Barruet, E., Nasu, A., et al. (2013). Induced pluripotent stem cells from patients with human fibrodysplasia ossificans progressiva show increased mineralization and cartilage formation. Orphanet J. Rare Dis. *8*, 190.

Matsumoto, Y., Ikeya, M., Hino, K., Horigome, K., Fukuta, M., Watanabe, M., Nagata, S., Yamamoto, T., Otsuka, T., and Toguchida, J. (2015). New protocol to optimize iPS cells for genome analysis of fibrodysplasia ossificans progressiva. Stem Cells *33*, 1730–1742.

Mishina, Y., Crombie, R., Bradley, A., and Behringer, R.R. (1999). Multiple roles for activin-like kinase-2 signaling during mouse embryogenesis. Dev. Biol. *213*, 314–326.

Miyazono, K., Kamiya, Y., and Morikawa, M. (2010). Bone morphogenetic protein receptors and signal transduction. J. Biochem. *147*, 35–51.

Moasser, M.M. (2007). Targeting the function of the HER2 oncogene in human cancer therapeutics. Oncogene *26*, 6577–6592.

Moffat, J.G., Vincent, F., Lee, J.A., Eder, J., and Prunotto, M. (2017). Opportunities and challenges in phenotypic drug discovery: an industry perspective. Nat. Rev. Drug Discov. *16*, 531–543.

Mohedas, A.H., Xing, X., Armstrong, K.A., Bullock, A.N., Cuny, G.D., and Yu, P.B. (2013). Development of an ALK2-biased BMP type I receptor kinase inhibitor. ACS Chem. Biol. *8*, 1291–1302.

Mueller, T.D., and Nickel, J. (2012). Promiscuity and specificity in BMP receptor activation. FEBS Lett. *586*, 1846–1859.

Nagasawa, J., Mizokami, A., Koshida, K., Yoshida, S., Naito, K., and Namiki, M. (2006). Novel HER2 selective tyrosine kinase inhibitor, TAK-165, inhibits bladder, kidney and androgen-independent prostate cancer in vitro and in vivo. Int. J. Urol. *13*, 587–592.

Nakajima, T., Shibata, M., Nishio, M., Nagata, S., Alev, C., Sakurai, H., Toguchida, J., and Ikeya, M. (2018). Modeling human somite development and fibrodysplasia ossificans progressiva with induced pluripotent stem cells. Development *145*. https://doi.org/10.1242/dev.165431.

Nasu, A., Ikeya, M., Yamamoto, T., Watanabe, A., Jin, Y., Matsumoto, Y., Hayakawa, K., Amano, N., Sato, S., Osafune, K., et al. (2013). Genetically matched human iPS cells reveal that propensity for cartilage and bone differentiation differs with clones, not cell type of origin. PLoS One *8*, e53771.

Ohnishi, K., Semi, K., Yamamoto, T., Shimizu, M., Tanaka, A., Mitsunaga, K., Okita, K., Osafune, K., Arioka, Y., Maeda, T., et al. (2014). Premature termination of reprogramming in vivo leads to cancer development through altered epigenetic regulation. Cell *156*, 663–677.

Pavey, G.J., Qureshi, A.T., Tomasino, A.M., Honnold, C.L., Bishop, D.K., Agarwal, S., Loder, S., Levi, B., Pacifici, M., Iwamoto, M., et al. (2016). Targeted stimulation of retinoic acid receptor-gamma mitigates the formation of heterotopic ossification in an established blast-related traumatic injury model. Bone *90*, 159–167.

Piek, E., Heldin, C.H., and Ten Dijke, P. (1999). Specificity, diversity, and regulation in TGF-beta superfamily signaling. FASEB J. *13*, 2105–2124.

Pignolo, R.J., Bedford-Gay, C., Liljesthrom, M., Durbin-Johnson, B.P., Shore, E.M., Rocke, D.M., and Kaplan, F.S. (2016). The natural history of flare-ups in fibrodysplasia ossificans progressiva (FOP): a comprehensive global assessment. J. Bone Miner. Res. *31*, 650–656.

Sanvitale, C.E., Kerr, G., Chaikuad, A., Ramel, M.C., Mohedas, A.H., Reichert, S., Wang, Y., Triffitt, J.T., Cuny, G.D., Yu, P.B., et al. (2013). A new class of small molecule inhibitor of BMP signaling. PLoS One *8*, e62721.

Shimono, K., Tung, W.E., Macolino, C., Chi, A.H., Didizian, J.H., Mundy, C., Chandraratna, R.A., Mishina, Y., Enomoto-Iwamoto, M., Pacifici, M., et al. (2011). Potent inhibition of heterotopic ossification by nuclear retinoic acid receptor-gamma agonists. Nat. Med. *17*, 454–460.

Shore, E., Feldman, G., Xu, M., and Kaplan, F. (2005). The genetics of fibrodysplasia ossificans progressiva. Clin. Rev. Bone Miner. Metab. *3*, 201–204.

Shore, E.M., and Kaplan, F.S. (2010). Inherited human diseases of heterotopic bone formation. Nat. Rev. Rheumatol. *6*, 518–527.

Shore, E.M., Xu, M., Feldman, G.J., Fenstermacher, D.A., Cho, T.J., Choi, I.H., Connor, J.M., Delai, P., Glaser, D.L., LeMerrer, M., et al. (2006). A recurrent mutation in the BMP type I receptor ACVR1 causes inherited and sporadic fibrodysplasia ossificans progressiva. Nat. Genet. *38*, 525–527.

Shukunami, C., Ishizeki, K., Atsumi, T., Ohta, Y., Suzuki, F., and Hiraki, Y. (1997). Cellular hypertrophy and calcification of embryonal carcinoma-derived chondrogenic cell line ATDC5 in vitro. J. Bone Miner. Res. *12*, 1174–1188.

Sinha, S., Uchibe, K., Usami, Y., Pacifici, M., and Iwamoto, M. (2016). Effectiveness and mode of action of a combination therapy for heterotopic ossification with a retinoid agonist and an anti-inflammatory agent. Bone *90*, 59–68.

Takahashi, M., Katagiri, T., Furuya, H., and Hohjoh, H. (2012). Disease-causing allele-specific silencing against the ALK2 mutants, R206H and G356D, in fibrodysplasia ossificans progressiva. Gene Ther. *19*, 781–785.

Umeda, K., Zhao, J., Simmons, P., Stanley, E., Elefanty, A., and Nakayama, N. (2012). Human chondrogenic paraxial mesoderm, directed specification and prospective isolation from pluripotent stem cells. Sci. Rep. *2*, 455.

Urist, M.R. (1965). Bone: formation by autoinduction. Science *150*, 893–899.

Wang, H., Lindborg, C., Lounev, V., Kim, J.H., McCarrick-Walmsley, R., Xu, M., Mangiavini, L., Groppe, J.C., Shore, E.M., Schipani, E., et al. (2016). Cellular hypoxia promotes heterotopic ossification by amplifying BMP signaling. J. Bone Miner. Res. *31*, 1652–1665.

Woltjen, K., Michael, I.P., Mohseni, P., Desai, R., Mileikovsky, M., Hamalainen, R., Cowling, R., Wang, W., Liu, P., Gertsenstein, M., et al. (2009). piggyBac transposition reprograms fibroblasts to induced pluripotent stem cells. Nature *458*, 766–770.

Wozney, J.M., Rosen, V., Celeste, A.J., Mitsock, L.M., Whitters, M.J., Kriz, R.W., Hewick, R.M., and Wang, E.A. (1988). Novel regulators of bone formation: molecular clones and activities. Science *242*, 1528–1534.

Yamada, K., Ohno, T., Aoki, H., Semi, K., Watanabe, A., Moritake, H., Shiozawa, S., Kunisada, T., Kobayashi, Y., Toguchida, J., et al. (2013). EWS/ATF1 expression induces sarcomas from neural crest-derived cells in mice. J. Clin. Invest. *123*, 600–610.

Yu, P.B., Deng, D.Y., Lai, C.S., Hong, C.C., Cuny, G.D., Bouxsein, M.L., Hong, D.W., McManus, P.M., Katagiri, T., Sachidanandan, C., et al. (2008). BMP type I receptor inhibition reduces heterotopic [corrected] ossification. Nat. Med. *14*, 1363–1369.

Zuscik, M.J., Hilton, M.J., Zhang, X., Chen, D., and O'Keefe, R.J. (2008). Regulation of chondrogenesis and chondrocyte differentiation by stress. J. Clin. Invest. *118*, 429–438.

Stem Cell Reports, Volume *11*

Supplemental Information

An mTOR Signaling Modulator Suppressed Heterotopic Ossification of Fibrodysplasia Ossificans Progressiva

Kyosuke Hino, Chengzhu Zhao, Kazuhiko Horigome, Megumi Nishio, Yasue Okanishi, Sanae Nagata, Shingo Komura, Yasuhiro Yamada, Junya Toguchida, Akira Ohta, and Makoto Ikeya

SUPPLEMENTAL EXPERIMENTAL PROCEDURES

Study approval. All experimental protocols dealing with human subjects were approved by the Ethics Committee of the Department of Medicine and Graduate School of Medicine, Kyoto University. Written informed consent was provided by each donor. All animal experiments were approved by the institutional animal committee of Kyoto University.

Cell culture. ATDC5 cells were maintained in DMEM/F-12 (Thermo Fisher Scientific) supplemented with 5% (v/v) FBS (Nichirei). iPSCs were maintained in primate ES cell medium (ReproCELL) supplemented with 4 ng/mL recombinant human FGF2 (Wako Pure Chemical). To induce induced neural crest cells (iNCCs), mTeSR1 medium (STEMCELL Technology) was used for the feeder-free culture of iPSCs. The induction and maintenance of iNCCs and iMSCs derived from iPSCs were previously described (Fukuta et al., 2014; Matsumoto et al., 2015). Briefly, iNCCs were induced in chemically defined medium (CDM) supplemented with 10 µM SB-431542 and 1 µM CHIR99021 for 7 days. iNCCs were maintained in CDM supplemented with 10 µM SB-431542, 20 ng/mL FGF2 and 20 ng/mL recombinant human EGF (R&D Systems) for up to 20 passages. iMSCs were induced and maintained in αMEM (Thermo Fisher Scientific) supplemented with 10% (v/v) FBS (Nichirei), 5 ng/mL FGF2 and 0.5% penicillin and streptomycin (Thermo Fisher Scientific). The FOP-iPSCs used in this study (previously described as vFOP4-1 (Matsumoto et al., 2013)) harbor the R206H heterozygous mutation in ACVR1, and gene-corrected resFOP-iPSCs were generated by BAC-based homologous recombination. These cells fulfilled several criteria for iPSCs including the expression of pluripotent markers, teratoma formation, normal karyotype and morphology. Growth and gene expression profiles of the resFOP-iPSC clones were indistinguishable from the original FOP-iPSCs (Matsumoto et al., 2015), however, remarkably distinct responsiveness to Activin-A was observed (Hino et al., 2015). C3H10T1/2 (murine multipotent mesenchymal cells)-expressing Dox-inducible hINHBA (C3H-DoxOn-hINHBA) was maintained in DMEM（Nacalai Tesque) supplemented with 10% FBS and 1 mM Na-pyruvate (Thermo Fisher Scientific) and used for an Activin-A-induced HO model transplanted with FOP-iMSCs as previously reported (Hino et al., 2015). In the wound healing assay, healthy control iPSC (414C2)-derived iMSCs were used (Okita et al., 2011).

Reagents. Activin-A, BMP-4, BMP-7 and TGF-β3 were purchased from R&D Systems. Rapamycin was purchased from MedChemexpress. SB-431542 was purchased from Sigma-Aldrich. TAK 165, PD 161570, CP-724714 and Lapatinib were purchased from Selleck Chemicals. DMH-1 was purchased from Tocris Bioscience. AZD0530 was purchased from ANgene. Trastuzumab and Pertuzumab were purchased from BioVision or Creative-Biolabs, respectively. Activin-A, BMP-7 and TGF-β3 were dissolved according to the manufacturer's protocols and used at 100 ng/mL (Activin-A and BMP-7) or 10 ng/mL (BMP-4 and TGF-β3) unless otherwise noted.

Chemicals Libraries. All chemical libraries were purchased from the suppliers listed. Almost all compounds were bioactive and/or annotated.

Library Name	Supplier	Number
Microsource International Drugs	MicroSource Discovery Systems	238
Microsource US Drugs		1020
Enzo FDA	Enzo Life Sciences	636
Enzo ICCB		474
Enzo kinase inhibitors		76
LOPAC1280	Sigma-Aldrich	1280
Sigma Pfizer		74
Myriascreen	TimTec	72
EMD Kinase Inhibitors	Merck Millipore	244
Selleck kinase inhibitors	Selleck Chemicals	141
Tocrs Mini Selected for CiRA	Tocris Bioscience	637
Total		4892

Generation of ATDC5 stably expressing ACVR1. WT- or FOP-ACVR1 was inserted into doxycycline (Dox)-inducible vector KW111 (Woltjen et al., 2009), which enables us to easily produce stably expressing cell lines utilizing the *piggyBac* (PB) transposon system from the cabbage looper moth *Trichoplusia ni* (Ding et al., 2005) (KW111/WT-ACVR1 or KW111/FOP-ACVR1). KW111/WT-ACVR1 or KW111/FOP-ACVR1 and PBaseII plasmid (PB transposase expression vector) (Matsui et al., 2014) were co-transfected into ATDC5 cells by FuGeneHD® (Promega) according to the manufacturer's protocol, and the neomycin-resistant population (500 µg/mL) was selected. Further selection was performed by selecting a mCherry high positive population (< 5%) after six hours of Dox treatment, sorting it with fluorescent-activated cell sorting (FACS) using AriaII (BD) according to the manufacturer's protocol, and designating it as ATDC5/WT-ACVR1 or ATDC5/FOP-ACVR1. KW111 included PB, active transposable PB elements; TRE-mCMVP, Tetracycline Response Element and a minimal CMV promoter; rtTA, reverse tetracycline-controlled transactivator. PBaseII was an expression plasmid vector containing the PB transposase cDNA with optimized codon usage to human under the control of the CAG promoter (Matsui et al., 2014).

HTS campaign and follow-up screens. ATDC5/FOP-ACVR1 cells were plated in 96-well white plates (2,000 cells/well/40 µL, Corning) in DMEM/F-12 supplemented with 5% (v/v) FBS. Two hours after incubation at 37 °C under 5% CO_2, 10 µL of test compounds (final 1 µM) was added, and assay plates were incubated at 37 °C under 5% CO_2. After 3 days incubation, Alkaline Phosphatase activity (ALP) or AlamarBlue activity was measured using Amplite™ Colorimetric Alkaline Phosphatase Assay Kit (AAT Bioquest) or alamarBlue® Cell Viability Reagent (Thermo Fisher Scientific) according to the manufacturer's protocol, respectively. The absorbance at 400 nm (Abs for ALP) or Relative Fluorescent Unit of Ex560/Em590 nm (RFU for AlamarBlue) was measured on POWERSCAN4 (DS Pharma Biomedical) or EnVision® Multilabel Reader (PerkinElmer). Inhibitory effects of the screened compounds are given as percent inhibition, which was calculated using the following equation: (1-([Abs of compound] - [Abs of Min])/([Abs of Max] - [Abs of Min]))× 100, where [Abs of Max] and [Abs of Min] are the mean Abs of DMSO control and 1 µM DMH-1, respectively. The Z' factor, which is widely used as a measure of the assay quality of each plate, was calculated using the following equation: Z' factor = 1 - 3 × ([SD of Max] + [SD of Min])/([Abs of Max] - [Abs of Min]) (SI Appendix, Fig. S3A). The average Z' factor in the HTS assay was 0.74, indicating accuracy and reliability of the HTS campaign (general criteria of HTS > 0.50) (Zhang et al., 1999). The S/B (signal-to-background) ratio was calculated using the following equation: ([Abs of Max]/[Abs of Min]) (SI Appendix, Fig. S3B). The average S/B ratio in the HTS assay was 6.9, also indicating accuracy and reliability of the HTS campaign (general criteria of HTS > 3.0).

Using ATDC5/FOP-ACVR1 cells, we performed a first screening (n=2; test compounds = 1 µM, Fig. 2A and B) against 4,892 small molecule compounds. In the first screening, 160 hit compounds satisfied the criteria more than 40% inhibition of ALP activity against DMSO control cells, less than 40% inhibition of viability, and margin (Inhibition of ALP activity (%) - Inhibition of viability (%)) more than 20%. A second screening was performed against the above 160 compounds (n=2; test compounds = 0.1, 0.3, 1, 3 µM), and 79 hit compounds satisfied the criteria 40% inhibition of ALP activity against DMSO control cells and margin more than 50% at any dose (SI Appendix, Fig. S4 and S5). To explore compounds which have the potential to identify new mechanisms or contribute to future drug repositioning, we selected 14 compounds, and detailed concentration-dependent assays were performed (Fig. 2E and SI Appendix, Fig. S6). As a result, we identified 7 compounds which showed stronger IC50 (< 500 nM) and less toxicity (Viability @ 10 µM > 50%) through the HTS campaign that focused on the constitutive activity of FOP-ACVR1 (Fig. 2E, red).

2D chondrogenic induction. Chondrogenic induction was performed, and differentiation properties were assayed as previously described (Hino et al., 2015; Nasu et al., 2013; Umeda et al., 2012). Briefly, iMSCs (1.5 × 10^5) were cultured in fibronectin-coated 24-well plates (BD Biosciences) using the chondrogenic medium with 100 ng/mL Activin-A and inhibitors until day 7 unless otherwise noted. Gene-specific siRNAs were purchased from Thermo Fisher Scientific (Silencer® Select Pre-designed siRNA). For the transient expression of siRNA, Lipofectamine® RNAiMAX Reagent (Thermo Fisher Scientific) was used according to the manufacturer's instructions. Knockdown efficiencies (n=1) of MTOR and ERBB2 16 h after transfection in FOP-iMSCs were 23.8% and 21.1%, respectively.

siRNA sequences.

Gene	ID	Sense	Antisense
MTOR	s604	GGAGCCUUGUUGAUCCUUAtt	UAAGGAUCAACAAGGCUCCat
ERBB2	s611	GCUCAUCGCUCACAACCAAtt	UUGGUUGUGAGCGAUGAGCac

Cardiotoxin-induced HO model in human FOP-ACVR1 conditional transgenic mice. Female hFOP-ACVR1 conditional transgenic mice (Beard et al., 2006; Hino et al., 2017; Ohnishi et al., 2014; Yamada et al., 2013) (f5-6 offspring of chimeric mice, age- and body weight-matched between groups) were used between 16 and 20 weeks of age. Mice were administered 2 mg/mL Dox in their drinking water supplemented with 10 mg/mL sucrose to induce FOP-ACVR1. Cardiotoxin (9.1 µg/mouse, Latoxan) was injected into the right gastrocnemius muscle to initiate skeletal muscle injury and subsequent heterotopic bone formation (Chakkalakal et al., 2012). Test compounds (16% DMSO in 0.5 w/v% Methyl Cellulose 400) were intraperitoneally administered once a day, five times a week. Mice were analyzed three weeks after injection. For X-ray images, mice were anesthetized with isoflurane (5% for induction, 2-3% for maintenance, Abbvie), immobilized and X-rayed using µFX-1000 (Fujifilm) or DX-50 (Faxitron Bioptics). µCT images were scanned using X-ray CT systems (inspeXio SMX-100CT, Shimadzu) and analyzed with TRI/3D-BON software (Ratoc System Engineering) according to the manufacturer's instructions. Four weeks after injection, the injected sites were harvested, fixed with 4% paraformaldehyde for 24 h, embedded in paraffin, and sectioned and stained with HE, von Kossa, Safranin O and anti-Collagen I antibody as previously described (Hino et al., 2015; Umeda et al., 2012).

BMP-7-induced HO model mice. BMP-7 (2 µg/mouse) was injected into the right gastrocnemius muscle of C57BL/6NJcl mice (6-8 weeks, male, CLEA Japan), and compounds were administered once a day, five times a week (intraperitoneal administration). Mice were analyzed 2 weeks after injection.

Activin-A-induced HO model transplanted with FOP-iMSCs. FOP- (right leg) and resFOP-iMSCs (left leg) (4×10^6 cells, respectively) were transplanted into the gastrocnemius muscle of NOD/ShiJic-scid Jcl (NOD/SCID) mice (8 weeks, male, CLEA Japan) with C3H-DoxOn-hINHBA (5×10^5 cells), which can continuously expose Activin-A to the transplanted iMSCs in vivo (Hino et al., 2015). In the Dox-induced group, 1 mg/mL Dox (Sigma-Aldrich) was administered via drinking water with 10 mg/mL sucrose (Nacalai Tesque) for two weeks after transplantation. Compounds were intraperitoneally administered once a day, five times a week. Eight weeks after transplantation, the transplanted cells were harvested, fixed with 4% paraformaldehyde for 24 h, embedded in paraffin, and sectioned and stained with HE, von Kossa, Safranin O, human specific anti-Vimentin antibody and Collagen I antibody as previously described (Hino et al., 2015; Umeda et al., 2012; Yamashita et al., 2015).

Histological analysis of growth plates chondrocytes. Knee joints were obtained from BMP-7-injected and test compounds-administrated C57BL/6NJcl mice after 2 weeks treatment and fixed, paraffin-embedded, and sectioned and stained with Safranin O. The thickness of the proliferative and hypertrophic zones of the growth plate were measured using ImageJ software on images obtained using a Keyence microscope (Keyence America). The proliferative zone was defined as the region with flat chondrocytes stacked in longitudinal columns, and the hypertrophic zone as the region where chondrocytes are enlarged in size and form clusters.

Normal bone µCT. The left legs of mice were obtained from BMP-7-injected and test-compounds administrated C57BL/6NJcl mice after 2 weeks treatment. The region of interest (ROI) included the femur-tibia (entire femur in addition to the part of tibia above tibia/fibular junction).

Wound healing assay. Healthy control iPSC (414C2)-derived iMSCs (5×10^4) were cultured in 24-well plates using αMEM supplemented with 10% (v/v) FBS, 5 ng/mL FGF2 and 0.5% penicillin and streptomycin at 37 °C for 72 h to form a confluent monolayer. Gaps were created by scratching the plates with a sterile pipette tip (1000 µL). The cells were then washed with PBS to remove the detached cells and replaced with medium containing 1% (v/v) FBS in the presence of DMSO (control) or test compounds (10 nM Rapamycin or 1 µM TAK165). The cell culture plates were transferred to a Biostation CT (Nikon Corp., Tokyo, Japan) programmed to take photographs at t = 0 h and t = 24 h. The migrated area was determined by subtracting the wound area at

time point t = 24 h from t = 0 h, and relative cell migration was expressed as the ratio of the absolute cell migrations between the experimental and control groups by using CL-Quant software (Nikon Corp.).

Quantitative PCR analysis. Total RNA was purified with RNeasy Kit (Qiagen) and treated with DNase-one Kit (Qiagen) to remove genomic DNA. Total RNA (0.3 µg) was reverse transcribed for single-stranded cDNA using random primers and Superscript III reverse transcriptase (Thermo Fisher Scientific) according to the manufacturer's instructions. Quantitative PCR was performed with Thunderbird SYBR qPCR Mix (TOYOBO) and analyzed with the StepOne real-time PCR system (Applied Biosystems). All data (relative expression) were corrected by β-actin.

Primers for RT-qPCR.

Gene	Forward	Reverse
ACTB	CACCATTGGCAATGAGCGGTTC	AGGTCTTTGCGGATGTCCACGT
MTOR	GACGAGAGATCATCCGCCAG	ACAAGGGACCGCACCATAAG
ERBB2	GCTCCTCCTCGCCCTCTT	TGAGTTCCAGGTTTCCCTGC

Microarray experiments. 2D chondrogenic induction was performed in FOP-iMSCs stimulated with or without 100 ng/mL Activin-A, TAK 165, CP-724714 and Lapatinib. Seven days after incubation, mRNA was extracted. RNA was reverse transcribed, biotin-labeled and hybridized to GeneChip Human Gene 1.0 ST Expression Array, which was subsequently washed and scanned according to the manufacturer's instructions (Affymetrix). Raw CEL files were imported into GeneSpring GX 12.6.1 software (Agilent Technologies), and expression values were calculated with the RMA16 algorithm. Pathway analysis was performed by Ingenuity pathway analysis (Qiagen).

Western blotting. SDS-PAGE and blotting with whole-cell lysates were performed by standard procedures. Protein bands were detected with ECL Prime Western Blotting Detection Reagent (GE Healthcare) and visualized using BIO-RAD Molecular Imager® Chemi-Doc™ XRS+ with Image Lab™ software (Bio-Rad). All data (relative intensity) were corrected by β-actin.

Antibodies for western blotting and immunostaining.

	Name	Company	Cat. No	Concentration
1st antibody	Human Activin RIA/ALK-2 Antibody	R&D Systems)	AF637	1:100
	Phospho-S6 Ribosomal Protein (Ser235/236) Antibody	Cell Signaling Technology	#2211	1:1000
	Monoclonal Anti-b-Actin-Peroxidase clone AC-15	SIGMA-ALDRICH	A3854	1:200000
	Anti-human Vimentin antibody	Abcam	ab16700	1:40
	Collagen I Antibody	Novus Biologicals	NB600-408	1:100
2nd antibody	Anti-rabbit IgG, HRP-linked Antibody	Cell Signaling Technology	#7074	1:10000
	Goat anti-Rabbit IgG (H+L) Secondary Antibody, Alexa Fluor® 488 conjugate	Invitrogen	A-11008	1:100
	Goat anti-Rabbit IgG (H+L) Secondary Antibody, Alexa Fluor® 555 conjugate	Invitrogen	A-21428	1:500

GAG value. The GAG content was quantified in pellets with the Blyscan Glycosaminoglycan Assay Kit (Biocolor). The DNA content was quantified using PicoGreen dsDNA Quantitation Kit (Thermo Fisher Scientific).

Immunohistochemistry. Paraffin-embedded sections were deparaffinized. For human specific anti-Vimentin antibody, antigen retrieval was performed by autoclave (105 °C, 10 min). Samples were blocked with Blocking One (Nacalai Tesque) for 60 min and then incubated with human specific anti-Vimentin antibody (Abcam) or

anti-Collagen I antibody (Novus Biologicals) diluted in Can Get Signal® immunostain solution B (TOYOBO) for 16-18 h at 4 °C. Next, samples were washed several times in 0.2% tween20 (Sigma-Aldrich) in PBS and incubated with Goat anti-Rabbit IgG (H+L) secondary antibody, Alexa Fluor® 488 or 555 conjugate (Thermo Fisher Scientific) diluted in Can Get Signal® immunostain solution B for one hour at room temperature. DAPI (10 µg/mL) was used to counterstain nuclei. Samples were observed by BZ-9000E (KEYENCE).

Statistics. The statistical significance of all experiments was calculated by Prism 6 (GraphPad Software). P values less than 0.05 were considered statistically significant.

SUPPLEMENTAL REFERENCES

Beard, C., Hochedlinger, K., Plath, K., Wutz, A., and Jaenisch, R. (2006). Efficient method to generate single-copy transgenic mice by site-specific integration in embryonic stem cells. Genesis *44*, 23-28.

Chakkalakal, S.A., Zhang, D., Culbert, A.L., Convente, M.R., Caron, R.J., Wright, A.C., Maidment, A.D., Kaplan, F.S., and Shore, E.M. (2012). An Acvr1 R206H knock-in mouse has fibrodysplasia ossificans progressiva. J Bone Miner Res *27*, 1746-1756.

Ding, S., Wu, X., Li, G., Han, M., Zhuang, Y., and Xu, T. (2005). Efficient transposition of the piggyBac (PB) transposon in mammalian cells and mice. Cell *122*, 473-483.

Fukuta, M., Nakai, Y., Kirino, K., Nakagawa, M., Sekiguchi, K., Nagata, S., Matsumoto, Y., Yamamoto, T., Umeda, K., Heike, T., *et al.* (2014). Derivation of Mesenchymal Stromal Cells from Pluripotent Stem Cells through a Neural Crest Lineage using Small Molecule Compounds with Defined Media. PloS one *9*, e112291.

Hino, K., Horigome, K., Nishio, M., Komura, S., Nagata, S., Zhao, C., Jin, Y., Kawakami, K., Yamada, Y., Ohta, A., *et al.* (2017). Activin-A enhances mTOR signaling to promote aberrant chondrogenesis in fibrodysplasia ossificans progressiva. J Clin Invest *127*, 3339-3352.

Hino, K., Ikeya, M., Horigome, K., Matsumoto, Y., Ebise, H., Nishio, M., Sekiguchi, K., Shibata, M., Nagata, S., Matsuda, S., *et al.* (2015). Neofunction of ACVR1 in fibrodysplasia ossificans progressiva. Proceedings of the National Academy of Sciences of the United States of America *112*, 15438-15443.

Matsui, H., Fujimoto, N., Sasakawa, N., Ohinata, Y., Shima, M., Yamanaka, S., Sugimoto, M., and Hotta, A. (2014). Delivery of full-length factor VIII using a piggyBac transposon vector to correct a mouse model of hemophilia A. PloS one *9*, e104957.

Matsumoto, Y., Hayashi, Y., Schlieve, C.R., Ikeya, M., Kim, H., Nguyen, T.D., Sami, S., Baba, S., Barruet, E., Nasu, A., *et al.* (2013). Induced pluripotent stem cells from patients with human fibrodysplasia ossificans progressiva show increased mineralization and cartilage formation. Orphanet J Rare Dis *8*, 190.

Matsumoto, Y., Ikeya, M., Hino, K., Horigome, K., Fukuta, M., Watanabe, M., Nagata, S., Yamamoto, T., Otsuka, T., and Toguchida, J. (2015). New Protocol to Optimize iPS Cells for Genome Analysis of Fibrodysplasia Ossificans Progressiva. Stem Cells *33*, 1730-1742.

Nasu, A., Ikeya, M., Yamamoto, T., Watanabe, A., Jin, Y., Matsumoto, Y., Hayakawa, K., Amano, N., Sato, S., Osafune, K., *et al.* (2013). Genetically matched human iPS cells reveal that propensity for cartilage and bone differentiation differs with clones, not cell type of origin. PloS one *8*, e53771.

Ohnishi, K., Semi, K., Yamamoto, T., Shimizu, M., Tanaka, A., Mitsunaga, K., Okita, K., Osafune, K., Arioka, Y., Maeda, T., *et al.* (2014). Premature termination of reprogramming in vivo leads to cancer development through altered epigenetic regulation. Cell *156*, 663-677.

Okita, K., Matsumura, Y., Sato, Y., Okada, A., Morizane, A., Okamoto, S., Hong, H., Nakagawa, M., Tanabe, K., Tezuka, K., *et al.* (2011). A more efficient method to generate integration-free human iPS cells. Nature methods *8*, 409-412.

Umeda, K., Zhao, J., Simmons, P., Stanley, E., Elefanty, A., and Nakayama, N. (2012). Human chondrogenic paraxial mesoderm, directed specification and prospective isolation from pluripotent stem cells. Scientific reports *2*, 455.

Woltjen, K., Michael, I.P., Mohseni, P., Desai, R., Mileikovsky, M., Hamalainen, R., Cowling, R., Wang, W., Liu, P., Gertsenstein, M., *et al.* (2009). piggyBac transposition reprograms fibroblasts to induced pluripotent stem cells. Nature *458*, 766-770.
Yamada, K., Ohno, T., Aoki, H., Semi, K., Watanabe, A., Moritake, H., Shiozawa, S., Kunisada, T., Kobayashi, Y., Toguchida, J., *et al.* (2013). EWS/ATF1 expression induces sarcomas from neural crest-derived cells in mice. J Clin Invest *123*, 600-610.
Yamashita, A., Morioka, M., Yahara, Y., Okada, M., Kobayashi, T., Kuriyama, S., Matsuda, S., and Tsumaki, N. (2015). Generation of scaffoldless hyaline cartilaginous tissue from human iPSCs. Stem Cell Reports *4*, 404-418.
Zhang, J.H., Chung, T.D., and Oldenburg, K.R. (1999). A Simple Statistical Parameter for Use in Evaluation and Validation of High Throughput Screening Assays. Journal of biomolecular screening *4*, 67-73.

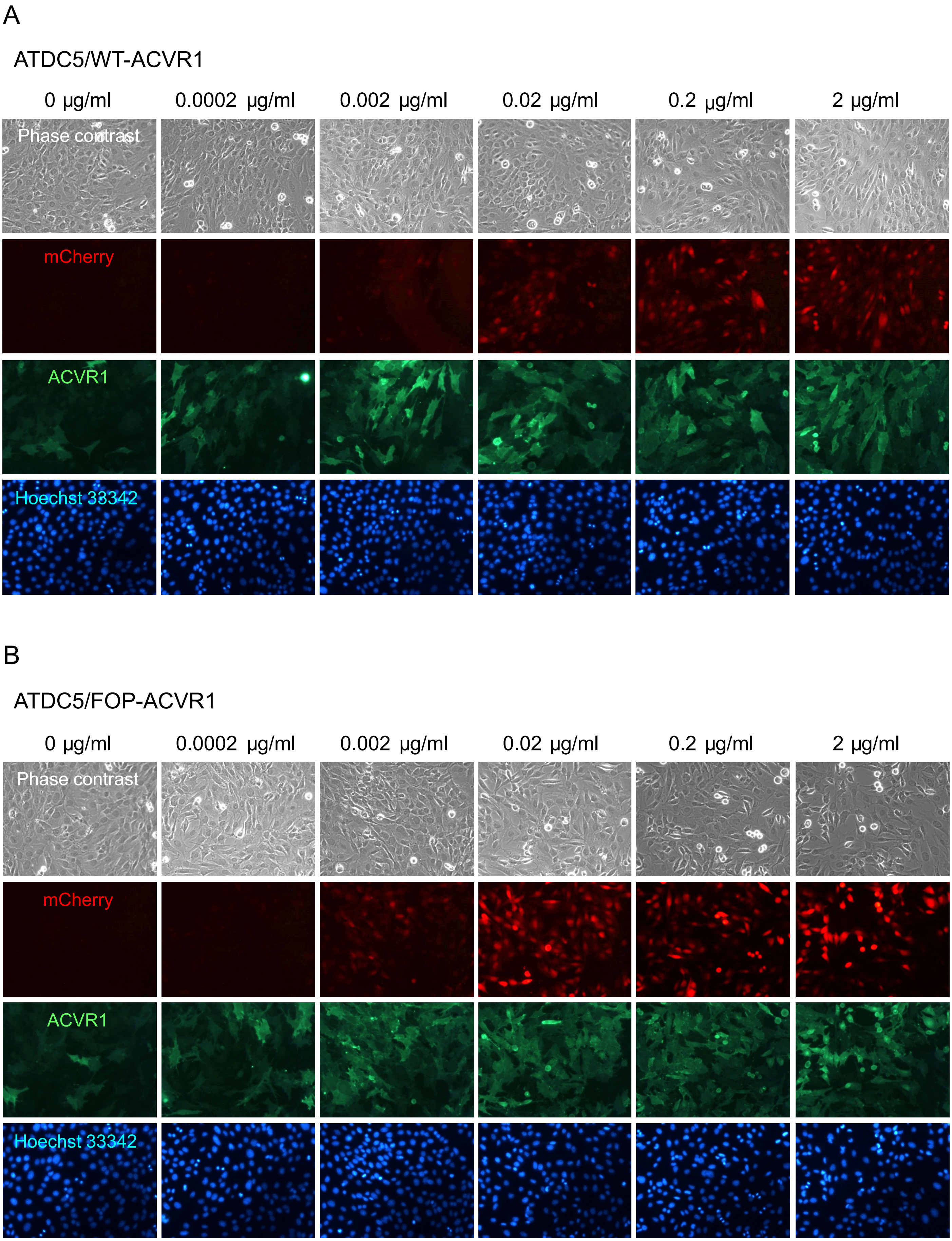

Figure S1. Expression of ACVR1 and mCherry in ATDC5/WT-ACVR1 or ATDC5/FOP-ACVR1. Related to Figure 1. 24 h after Dox treatment (0-2 µg/mL), cells were fixed and stained. Panels of DOX 0 and 0.002 µg/mL condition are same as Figure 1B (B). Scale bars, 100 µm.

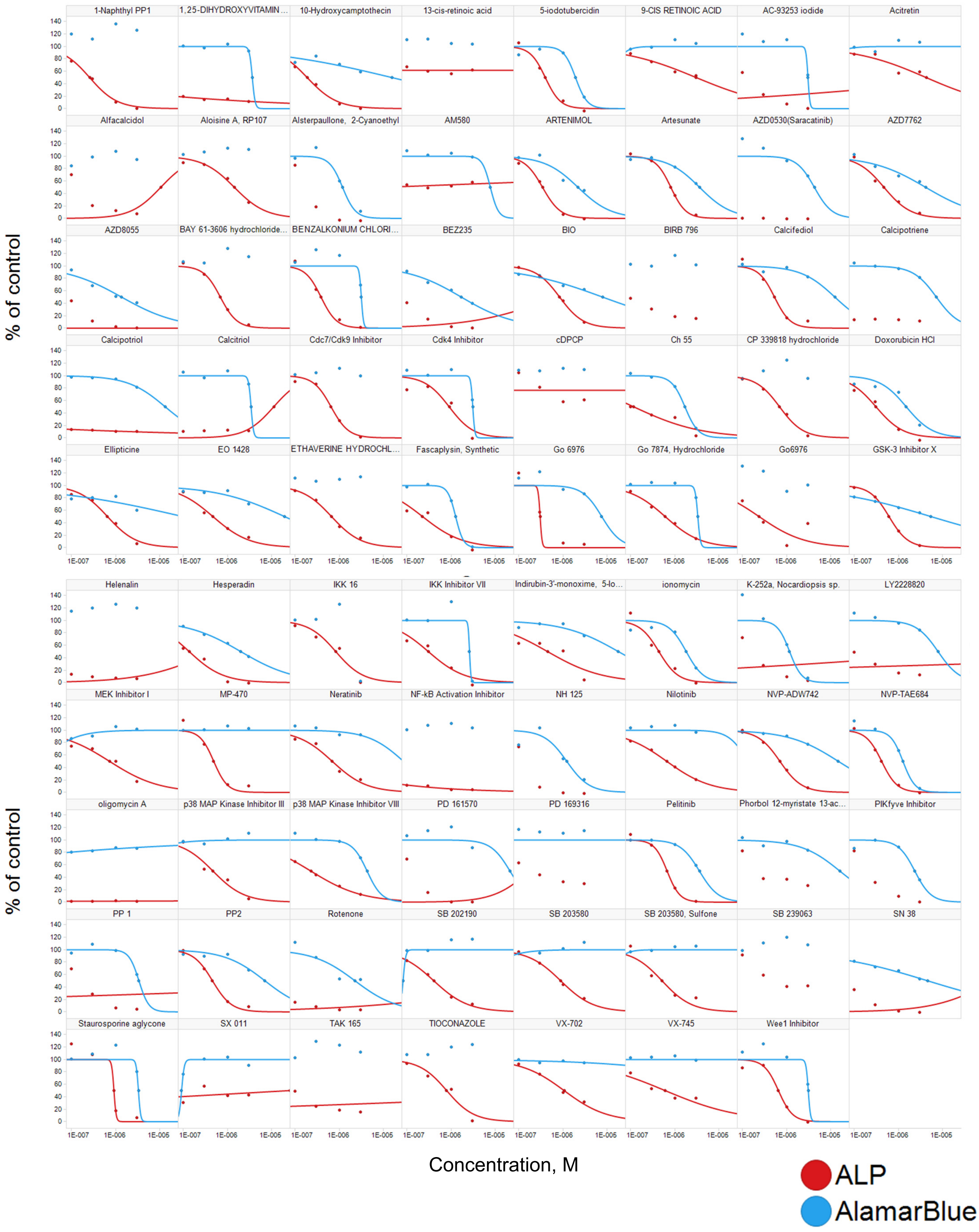

Figure S2. Dose-dependent assay results of 79 hit compounds. Related to Figure 2. ALP assay and AlamarBlue assay were performed using the same protocol as the HTS. Results are the mean of biological duplicate. 0.1, 0.3, 1, 3 µM of test compounds were assessed. Listed compounds showed more than 40% inhibition of ALP activity against DMSO control cells and margin more than 50% (inhibition of ALP activity (%) - inhibition of viability (%)) at any dose.

A

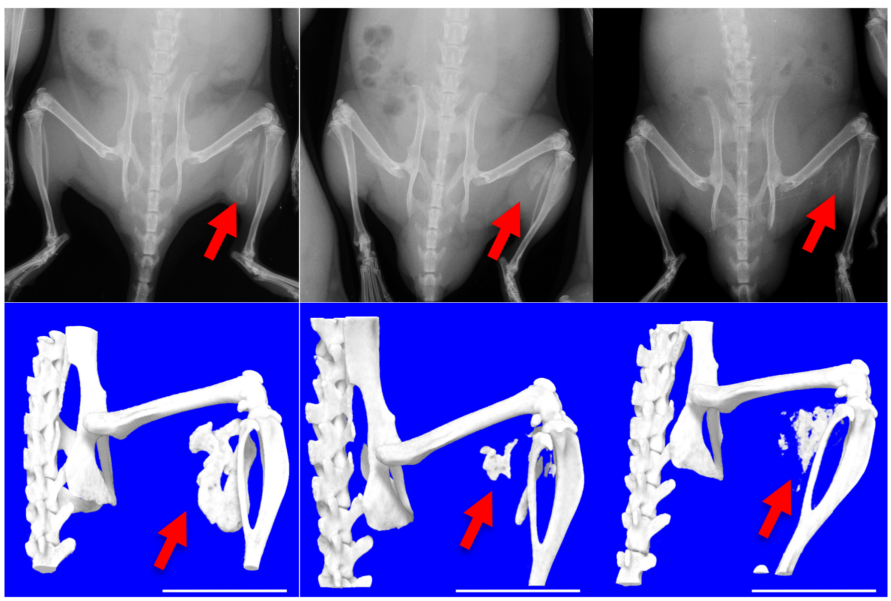

B

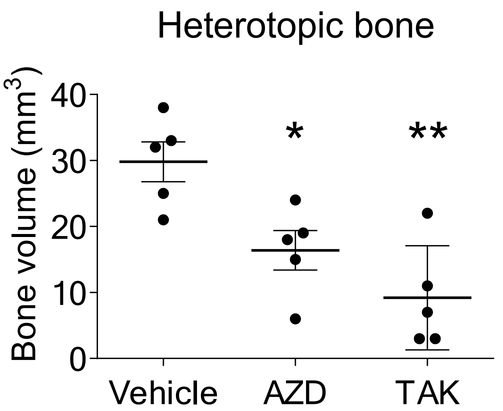

C

Compound	# of HO (> 20 mm^3)
Vehicle	5/5
AZD0530	1/5
TAK 165	1/5

D

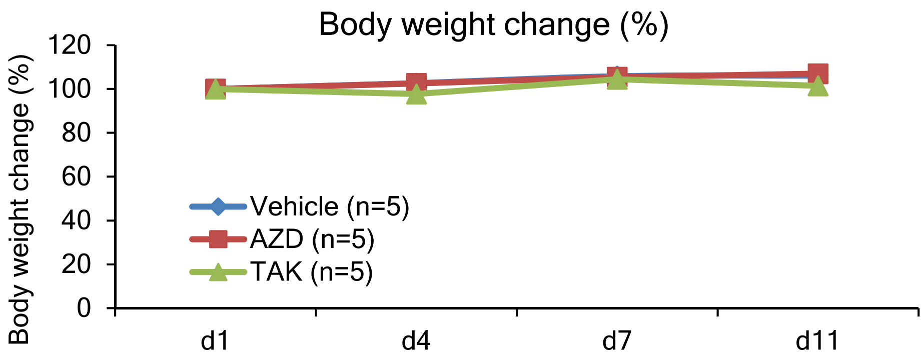

Figure S3. AZD0530 and TAK 165 suppressed BMP-7-induced HO. Related to Figure 5. BMP-7 was injected into the right gastrocnemius muscle of male C57BL/6 mice (6-8 weeks), who were administered drugs once daily 5 times a week intraperitoneally for 2 weeks thereafter. (A) X-rays (upper panels) and µCT (lower panels) observations. (B) Average heterotopic bone volume. (C) The number of mice harboring HO (> 20 mm^3 bone volume). (D) Body weight change of BMP-7-injected male C57BL/6 mice. Scale bars, 10 mm (A). Results are the mean ± standard error (SE). N = 5. *, $P < 0.05$; **, $P < 0.01$ by Dunnett’s multiple comparisons *t*-test compared to the vehicle treatment group (B). No significant differences between the AZD- or TAK- administered group compared to the vehicle group in two-way repeated measures ANOVA followed by Dunnett's multiple comparisons *t*-test (D).

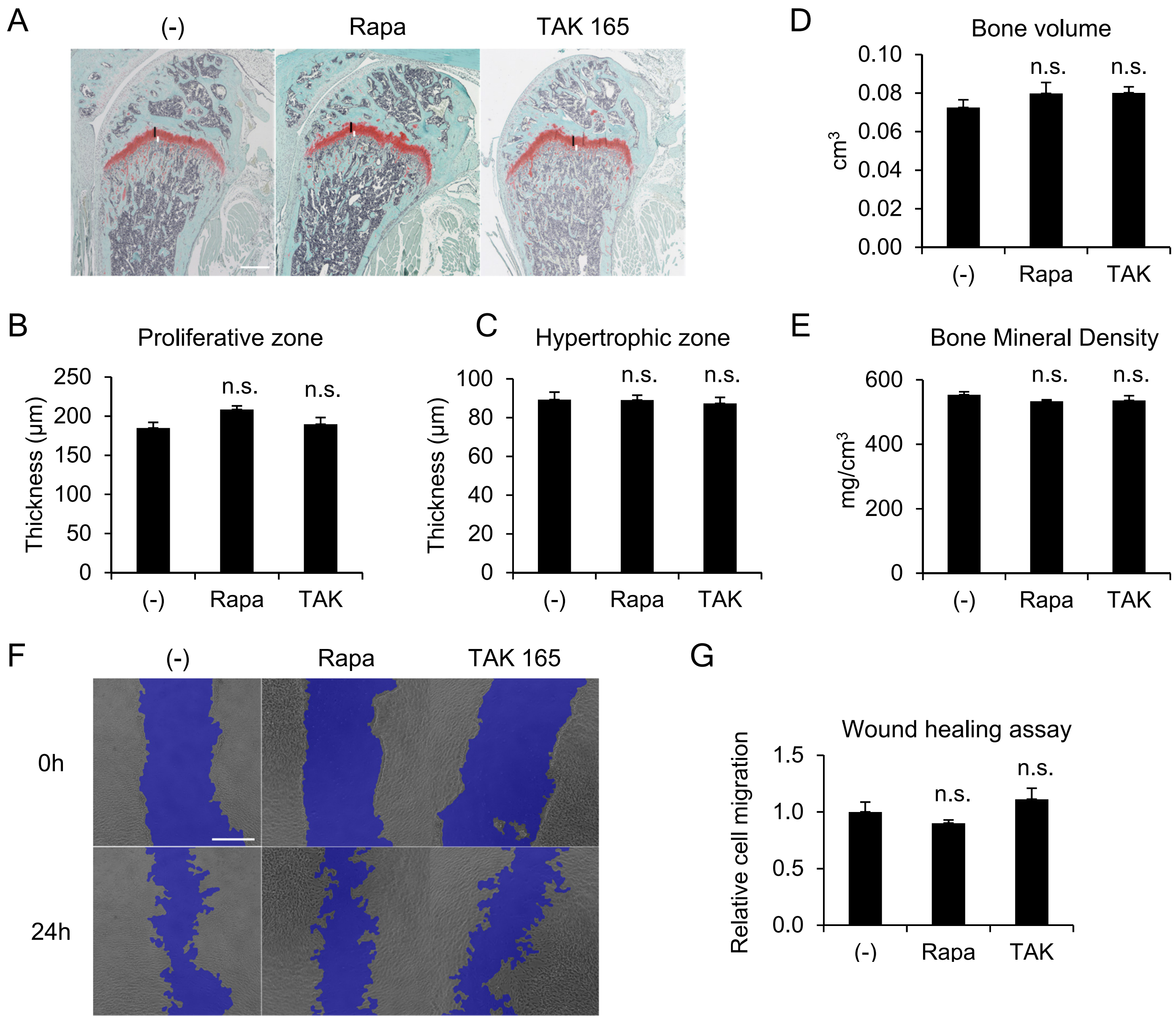

Figure S4. Tests of side effects of direct and indirect mTOR inhibitors. Related to Figure 5 and 6. (A-E) BMP-7 was injected into the right gastrocnemius muscle of male C57BL/6 mice (6-8 weeks), who were administered 5 mg/kg of compounds once daily 5 times a week intraperitoneally for 2 weeks thereafter. (A-C) Histological analysis of growth plate chondrocytes. Representative histological sections of the growth plate of tibiae from compounds-administered mice (A). Quantification of the thickness of the proliferative (B) and hypertrophic (C) zones is shown. Vertical lines indicate proliferative (black) and hypertrophic (white) zones. (C and D) Normal bone μCT of the femur-tibia of left legs from compounds-administered mice (F and G) Wound healing assay using iMSCs from healthy donor-derived iPSCs (414C2). iMSCs migrated to cover the scratched cell-free area. (E) Representative pictures and (F) relative migration values of iMSCs cultured in the medium with DMSO or test compounds (10 nM rapamycin or 1 μM TAK165). Scale bars, 0.5 mm (A and F). Results are the mean ± standard error (SE), n = 5 (Vehicle and TAK 165) or n = 4 (rapamycin) (A-E), or biological triplicate in 3 independent experiments (F and G). n.s., no significant difference by Dunnett's multiple comparisons *t*-test compared to the vehicle treatment group (B-E) or DMSO treatment control group (G).

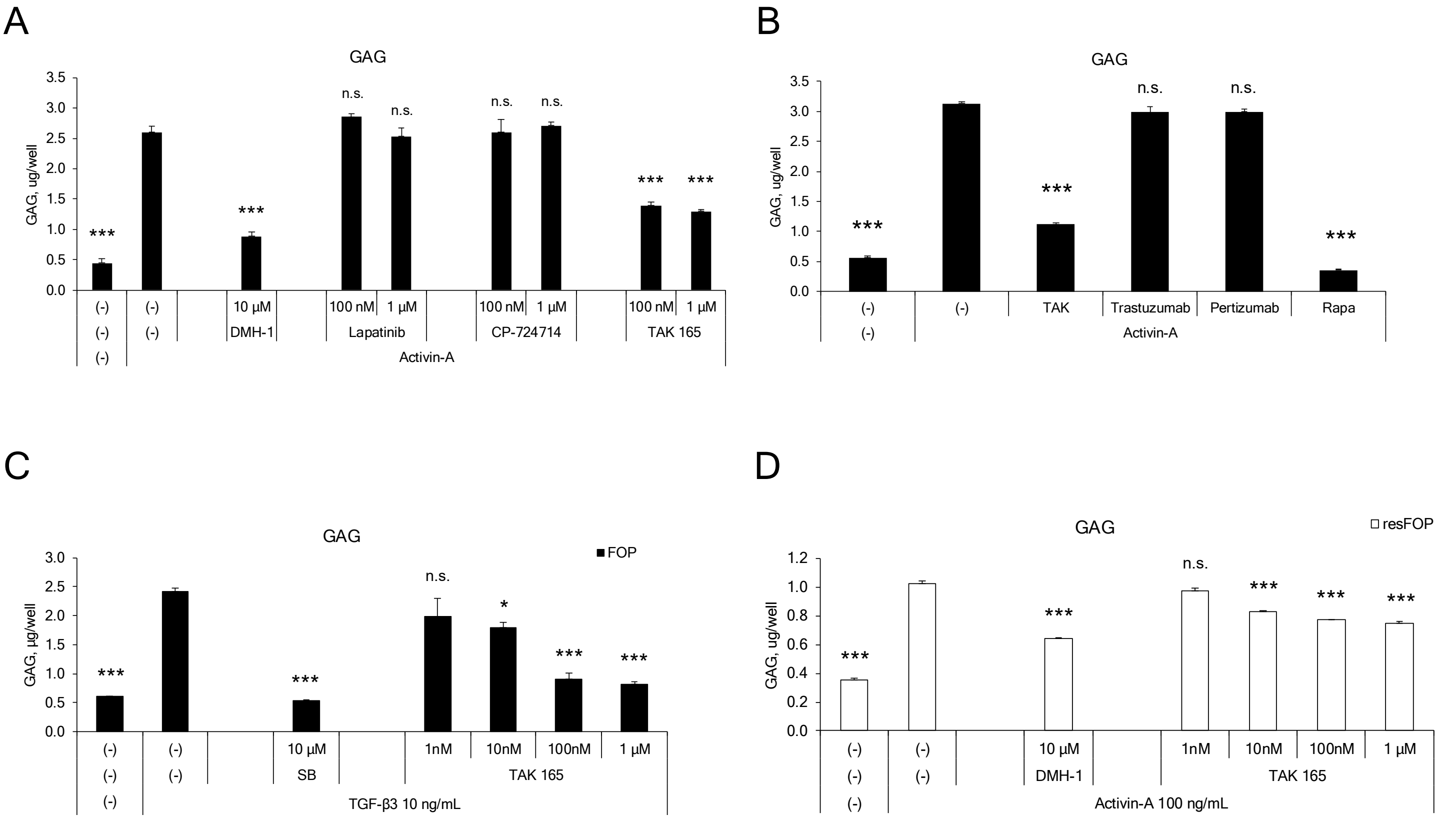

Figure S5. Analysis of the mechanism of TAK 165 action. Related to Figure 7. (A and B) ERBB2 inhibition did not suppress the chondrogenic induction of FOP-iMSCs. (A) Lapatinib (selective EGFR/ERBB2 inhibitor) and CP-724714 (selective ERBB2 inhibitor) or (B) 1 μg/mL Trastuzumab and 1 μg/mL Pertizumab (neutralizing antibodies against ERBB2) did not suppress the chondrogenesis of FOP-iMSCs. FOP-iMSCs were harvested 7 days after chondrogenic induction, which was performed with or without Activin-A, ERBB2 inhibitors or antibodies. TAK, 1 μM TAK 165; Rapa, 20 nM rapamycin. (C) TAK 165 suppressed the chondrogenesis of FOP-iMSCs stimulated with TGF-β3. (D) TAK 165 suppressed the chondrogenesis of resFOP-iMSCs stimulated with Activin-A. resFOP- or FOP-iMSCs were harvested 7 days after chondrogenic induction, which was performed with or without Activin-A or TGF-β3. Results are the mean ± standard error (SE) of biological triplicate using FOP-iPSCs (vFOP4-1) or its isogenic control iPSCs (resFOP-iPSCs). n.s., no significant difference; *, P < 0.05; ***, P < 0.001 by Dunnett's multiple comparisons *t*-test compared to the DMSO treatment control with Activin-A (A-C) or TGF-β3 (D).

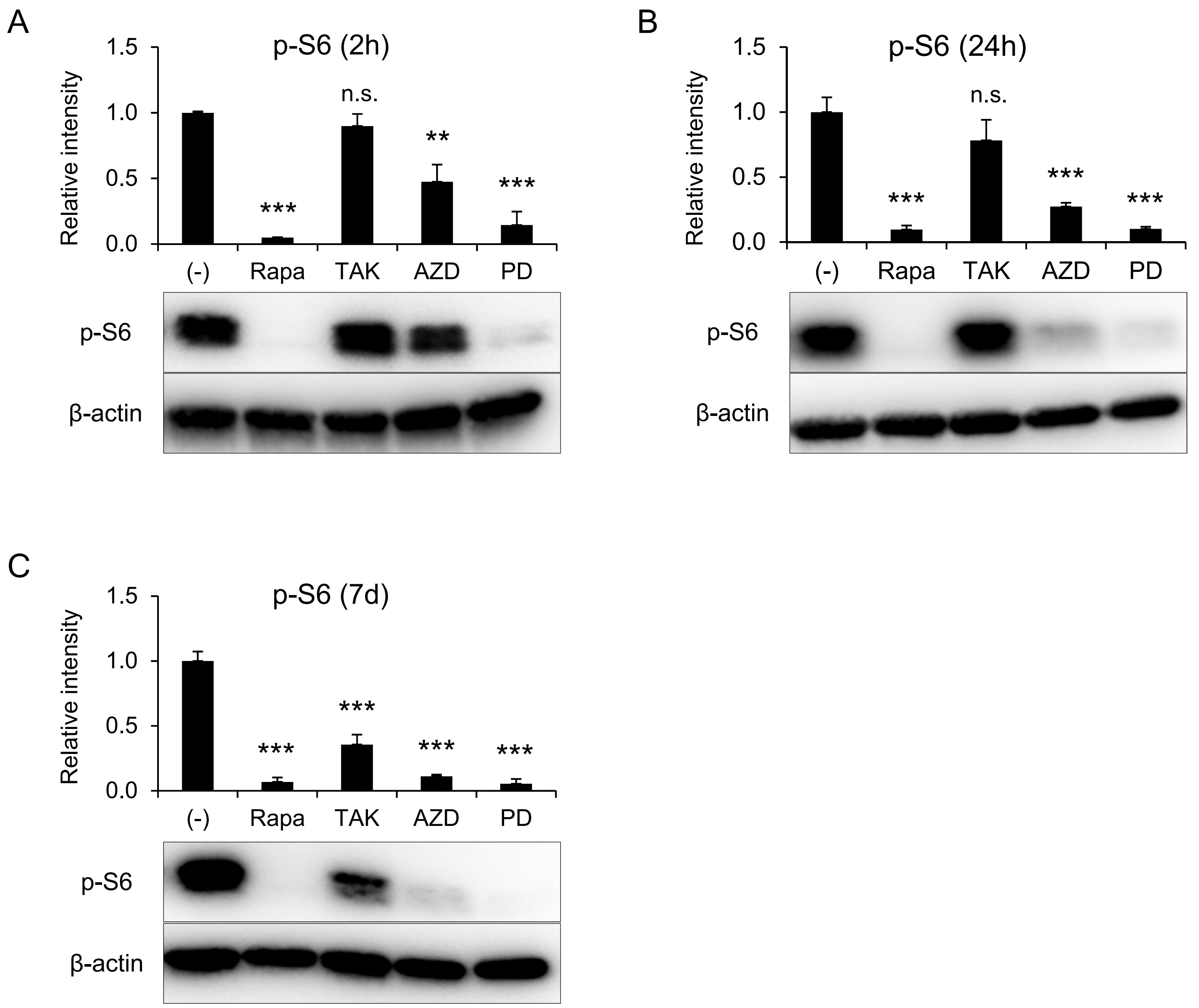

Figure S6. Distinct effects of AZD0530, PD 161570 and TAK 165 on mTOR signaling in chondrogenesis of FOP-iMSCs. Related to Figure 7. After 2 h (A), 24 h (B) or 7 days (C) of chondrogenic induction of FOP-iMSCs with Activin-A and test compounds, the cells were harvested, and p-S6 was assessed by western blotting. 10 nM rapamycin (Rapa) or 1 µM TAK 165 (TAK), AZD0530 (AZD) and PD 161570 (PD) were applied in the experiments. Results are the mean ± standard error (SE) of biological triplicate in 3 independent experiments using FOP-iPSCs (vFOP4-1). n.s., no significant difference; **, P < 0.01; ***, P < 0.001 by Dunnett's multiple comparisons *t*-test compared to the DMSO treatment control with Activin-A.

A

Experimental group	Activin-A	Compound
1	(+)	TAK 165
2	(+)	(-)
3	(+)	Lapatinib
4	(+)	CP-724714

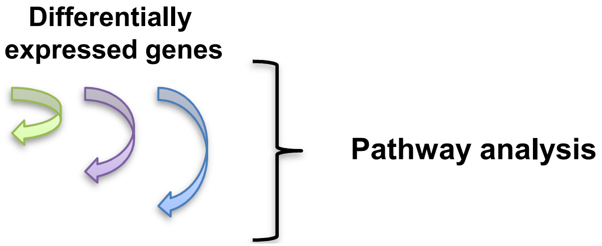

B

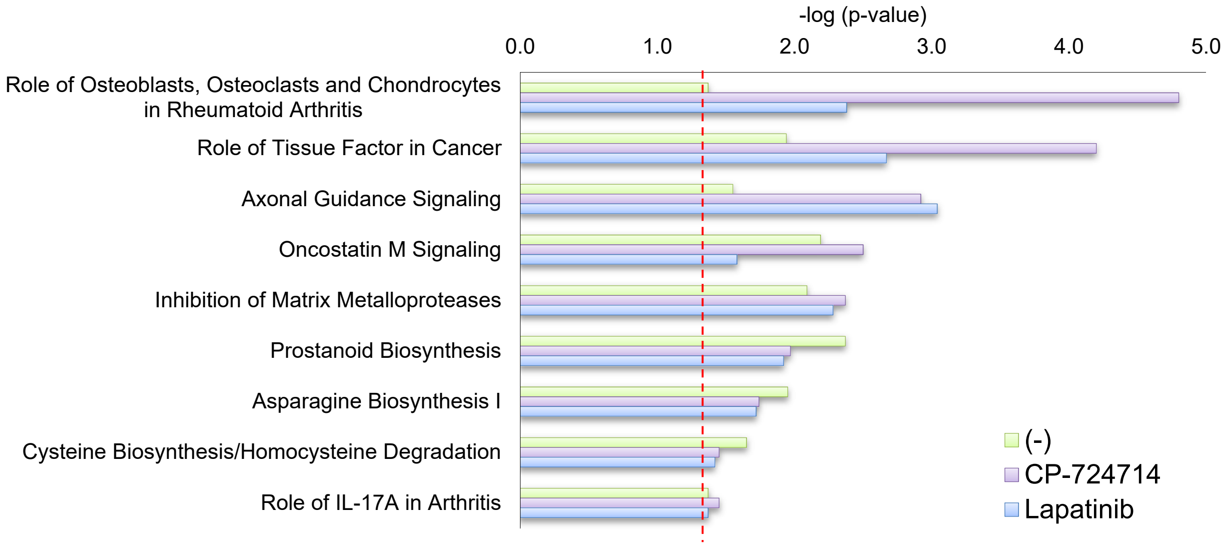

C

Symbol	Entrez Gene Name	(-)	Lapatinib	CP-724714
MMP1	matrix metallopeptidase 1	3.6	3.0	11.5
MMP13	matrix metallopeptidase 13	5.6	6.9	5.4
ITGA3	integrin subunit alpha 3	2.1	2.5	3.9
ITGB3	integrin subunit beta 3	2.8	3.6	3.3
FOS	Fos proto-oncogene, AP-1 transcription factor subunit	2.2	2.4	3.2
ADAMTS5	ADAM metallopeptidase with thrombospondin type 1 motif 5	0.5	2.1	3.1
BMP4	bone morphogenetic protein 4	0.5	0.4	5.2
DKK1	dickkopf WNT signaling pathway inhibitor 1	0.5	2.1	0.2
FRZB	frizzled-related protein	2.7	3.0	3.6
FZD3	frizzled class receptor 3	0.5	0.4	0.3
PIK3R3	phosphoinositide-3-kinase regulatory subunit 3	2.0	2.1	0.4
SPP1	secreted phosphoprotein 1	2.9	3.7	4.4
TNFRSF11B	TNF receptor superfamily member 11b	0.5	0.5	3.4
WIF1	WNT inhibitory factor 1	0.2	0.1	0.1
WNT16	Wnt family member 16	2.3	0.5	3.0

Figure S7. Pathway analysis of TAK 165 and other ERBB2 inhibitors in Activin-A-induced chondrogenesis of FOP-iMSCs. Related to Figure 7. Chondrogenic induction was performed in FOP-iMSCs with or without 100 ng/mL Activin-A, 100 nM TAK 165, 100 nM Lapatinib (selective EGFR/ERBB2 inhibitor) and 100 nM CP-724714 (selective ERBB2 inhibitor). After 7 days, cells were harvested, and microarray analysis was performed. (A) Experimental group and stimulation. (B and C) Differentially expressed genes (1.5 fold change) against the TAK 165-treated group were analyzed by Ingenuity Pathway Analysis. (B) Canonical Pathways significantly involved in all three groups. -log (p-value) > 1.3 (p = 0.05) was considered significant. (C) Expression ratio to the TAK 165-treated group of differentially expressed genes in “Role of Osteoblasts, Osteoclasts and Chondrocytes in Rheumatoid Arthritis” are shown. N = 1.
